# CD161^+^ Tconv and CD161^+^ Treg Share a Transcriptional and Functional Phenotype despite Limited Overlap in TCRβ Repertoire

**DOI:** 10.3389/fimmu.2017.00103

**Published:** 2017-03-06

**Authors:** Chantal L. Duurland, Chrysothemis C. Brown, Ryan F. L. O’Shaughnessy, Lucy R. Wedderburn

**Affiliations:** ^1^Infection, Inflammation and Rheumatology Section, Infection, Immunity and Inflammation Programme, UCL Great Ormond Street Institute of Child Health, University College London (UCL), London, UK; ^2^Immunobiology Section, Infection, Immunity and Inflammation Programme, UCL Great Ormond Street Institute of Child Health, University College London (UCL), London, UK; ^3^Arthritis Research UK Centre for Adolescent Rheumatology, UCL Great Ormond Street Institute of Child Health, University College London (UCL), London, UK; ^4^UK National Institute for Health Research (NIHR) GOSH Biomedical Research Centre, London, UK

**Keywords:** CD161, conventional T cells (Tconv), regulatory T cells (Treg), transcriptome, juvenile idiopathic arthritis (JIA), tissue homing, T cell receptor (TCR), retinoic acid

## Abstract

Human regulatory T cells (Treg) are important in immune regulation, but can also show plasticity in specific settings. CD161 is a lectin-like receptor and its expression identifies an effector-like Treg population. Here, we determined how CD161^+^ Treg relate to CD161^+^ conventional T cells (Tconv). Transcriptional profiling identified a shared transcriptional signature between CD161^+^ Tconv and CD161^+^ Treg, which is associated with T helper (Th)1 and Th17 cells, and tissue homing, including high expression of gut-homing receptors. Upon retinoic acid (RA) exposure, CD161^+^ T cells were more enriched for CCR9^+^ and integrin α4^+^β7^+^ cells than CD161^−^ T cells. In addition, CD161^+^ Tconv and CD161^+^ Treg were enriched at the inflamed site in autoimmune arthritis, and both CD161^+^ and CD161^−^ Treg from the inflamed site were suppressive *in vitro*. CD161^+^ T cells from the site of autoimmune arthritis showed a diminished gut-homing phenotype and blunted response to RA suggesting prior imprinting by RA in the gut or at peripheral sites rather than during synovial inflammation. TCRβ repertoires of CD161^+^ and CD161^−^ Tconv and Treg from blood showed limited overlap whereas there was clear overlap between CD161^+^ and CD161^−^ Tconv, and CD161^+^ and CD161^−^ Treg from the inflamed site suggesting that the inflamed environment may alter CD161 levels, potentially contributing to disease pathogenesis.

## Introduction

Human regulatory T cells (Treg) expressing high levels of CD25 (1), low levels of CD127 (2, 3), and the master transcription factor Foxp3 (4–6) are key to immune regulation. The importance of Treg is clearly illustrated by the onset of severe multi-organ autoimmune diseases in absence of functional Treg (7, 8). Treg are generally considered to lack the ability to produce pro-inflammatory cytokines (6). However, recent *ex vivo* and *in vitro* studies have demonstrated that a proportion of Treg from healthy individuals is able to produce pro-inflammatory cytokines associated with T helper (Th) lineages such as interferon (IFN)-γ and interleukin (IL)-17 (9–15). Analysis of cytokine-producing Treg in autoimmune diseases indicated an enrichment of IFNγ^+^Foxp3^+^ Treg in patients with type 1 diabetes (16) or multiple sclerosis (17), IL17^+^Foxp3^+^CD4^+^ T cells in patients with ulcerative colitis (18), Crohn’s disease (19) or psoriasis (20), and IFNγ- and IL-17-producing Treg in patients with autoimmune hepatitis (21) compared to healthy individuals. This suggests that at sites of inflammation, cytokine-producing Treg might actively promote inflammation instead of dampening it.

These effector-like characteristics of Treg raise questions about the role of these cells in health and disease. We have recently identified CD161 as a marker to identify a Treg population capable of producing pro-inflammatory cytokines. CD161^+^ Treg are suppressive in *in vitro* suppression assays and have a predominantly demethylated Treg-specific demethylated region (TSDR) (14). CD161, the human ortholog of murine natural killer receptor protein 1A (NKRP1A), is a lectin-like receptor initially identified as a marker for NK (T) cells (22, 23), but is also expressed on CD8^+^ T cells (24, 25), Th17 cells (26, 27), and innate lymphoid cells (ILC) (28). In addition, Th17 cells expressing CD161 can convert to Th1 cells under pro-inflammatory conditions and thereby retain CD161 expression (29, 30) suggesting that CD161 may mark cells capable of T cell plasticity in inflammatory conditions. Despite the effector-like phenotype of CD161^+^ Treg, it is unknown how these cells relate to CD161^+^ T effector cells.

In this study, we aimed to define the transcriptional and protein signatures, and TCRβ repertoire of CD161^+^ Treg and CD161^+^ conventional T cells (Tconv). CD161^+^ Treg and CD161^+^ Tconv shared transcriptional and protein signatures and expressed high levels of cell surface proteins associated with gut homing. However, the TCRβ repertoire of these cells showed limited overlap. Intriguingly, at the site of inflammation in patients with autoimmune arthritis, the TCRβ repertoire of CD161^+^ and CD161^−^ Tconv, and CD161^+^ and CD161^−^ Treg showed a considerable amount of overlap suggesting that CD161 expression can be altered in autoimmune conditions.

## Materials and Methods

### Human Samples

Peripheral blood (PB) samples from healthy adult and child volunteers or patients with juvenile idiopathic arthritis (JIA) and synovial fluid (SF) samples from JIA patients were obtained with full written informed consent and age appropriate assent as approved by the London—Bloomsbury Research Ethics Committee (ref 95RU04) in accordance with the Declaration of Helsinki. JIA patients were diagnosed according to internationally agreed criteria (31). PB and SF mononuclear cells (PBMC and SFMC) were prepared by density gradient centrifugation. Before processing, SF samples were treated with Hyaluronidase (10 U/ml; Sigma-Aldrich) for 30 min at 37°C.

### Cell Culture

Cells were cultured in RPMI1640-containing l-glutamine supplemented with penicillin (100 U/ml), streptomycin (100 µg/ml), and 10% FCS (all Thermo Fisher Scientific) at 37°C and 5% CO_2_. To assess cytokine production, cells were cultured with Phorbol Myristate Acetate (PMA) (50 ng/ml), Ionomycin (500 ng/ml) and Brefeldin A (5 µg/ml) (all Sigma-Aldrich) for 4 h, or recombinant human IL-12 (50 ng/ml; Pepro-Tech EC Ltd.), IL-18 (50 ng/ml; Bio-Techne) and Brefeldin A (5 µg/ml; last 4 h only) for 24 h. Cell cycle profile was analyzed after 4 days of culture in presence of plate-bound αCD3 (1 µg/ml; clone UCHT1, R&D Systems) and αCD28 (5 µg/ml; clone CD28.2, BD Pharmingen) antibodies. For cultures with all-trans retinoic acid (ATRA; Sigma-Aldrich), cells were cultured in serum free medium (Thermo Fisher Scientific) in absence or presence of plate-bound αCD3 (1 µg/ml) and αCD28 (5 µg/ml), and ATRA at concentrations indicated for 4 days (ATRA alone), or 48 h and then rested for 48 h (ATRA + TCR signal) before analysis.

### Flow Cytometry

Flow cytometry was performed by standard methods using directly conjugated monoclonal antibodies (Table S1 in Supplementary Material) against specific human cell surface or intracellular proteins. Dead cells were excluded by staining with a live/dead dye. Intracellular proteins were stained using Foxp3 staining kit (eBioscience). Cell cycle profile was analyzed using FxCycle Violet (Thermo Fisher Scientific). Data were acquired on LSRII flow cytometer (BD Biosciences) and analyzed using FlowJo software version 10.1 (Tree Star Inc.).

### Cell Sorting

PBMC from adult healthy controls or JIA patients, or SFMC from JIA patients were enriched for CD4^+^ T cells using EasySep Human CD4^+^ T Cell Enrichment kit (Stemcell Technologies) and stained for CD4, CD127, CD25, and CD161 using the monoclonal antibodies defined in Table S1 in Supplementary Material, before adding a live/dead dye. Cells were sorted on FACS Aria III (BD Biosciences) for the following live cell populations: CD161^+^ Tconv: CD4^+^CD127^+^CD25^−^CD161^+^, CD161^−^ Tconv: CD4^+^CD127^+^CD25^−^CD161^−^, CD161^+^ Treg: CD4^+^CD127^low^CD25^hi^CD161^+^, CD161^−^ Treg: CD4^+^CD127^low^CD25^hi^CD161^−^. A small aliquot of each sorted cell populations was stained for Foxp3 to assess sort purity.

### Treg Suppression Assay

CD161^+^ and CD161^−^ Treg and total Tconv were sorted from JIA SF. To assess suppression of proliferation, sorted Tconv were labeled with CellTrace Violet (Thermo Fisher Scientific) according to manufacturer’s instructions. Labeled Tconv were cultured at a constant number of 5 × 10^4^ cells per well, either alone (1:0) or with sorted CD161^+^ or CD161^−^ Treg at a 1:1 and, where cell numbers permitted, 2:1 ratio. Cells were cultured on a 96 well V-bottom plate pre-coated with plate-bound αCD3 (1 µg/ml) and αCD28 (5 µg/ml) antibodies. Final cell concentration was kept at 1 × 10^6^ cells per ml. Cells were cultured for 4 or 5 days at 37°C and 5% CO_2_ before analysis by flow cytometry. To determine suppression of proliferation, the % divided function in FlowJo version 7.6.5 (Tree Star Inc.) was used and % divided for Tconv alone was set at 0% suppression.

### RNA Extraction and RNAseq

RNA was extracted from sorted populations using Picopure kit (Thermo Fisher Scientific), and the cDNA library was prepared using TruSeq RNA sample preparation kit (Illumina). Samples were run on HiSeq 2500 (Illumina), rapid run mode 50PE with average read depth of 30 million reads. After sequencing, reads were mapped to the human genome (human NCBI genome build 37.2) and read count data for each transcript were obtained using the scripts provided by the Dexseq package. FPKM values were created using the normalization tools included in the Deseq2 package followed by subsequent analysis using GeneSpring 13.0 (Agilent Technologies) to determine differentially expressed (DE) genes using *P* < 0.05 (determined using unpaired *t*-test without correction for multiple testing) and fold change ≥1.5. One CD161^+^ Treg sample was excluded from analysis, because of low cDNA yield. Transcriptome data have been deposited in GEO under accession number GSE86452. Principal component analysis (PCA) of the dataset was performed using FactoMineR (32) in RStudio (version 3.2.2). Heatmaps were generated using GENE-E.^1^ Pathway analysis was performed using QIAGEN’s Ingenuity^®^ Pathway Analysis (IPA^®^, QIAGEN Redwood City^2^).

### TCRB Sequencing and Analysis

Genomic DNA (gDNA) was extracted as described (33) from purified CD161^+^ and CD161^−^ Tconv and Treg. 100–400 ng gDNA was processed by Adaptive Biotechnologies (Seattle, WA, USA) using the ImmunoSEQ human TCRB platform combining multiplex PCR with high throughput sequencing and a sophisticated bioinformatics pipeline for TCRB CDR3 analysis (34, 35). Data were analyzed using ImmunoSEQ Analyser (version 3.0) using only productive rearrangements at nucleotide level.

### Statistical Analysis

Statistical analysis of flow cytometry data was performed using Prism 5.03 for Windows (Graphpad). Where used, black lines or bars in summary graphs represent median and where used error bars represent interquartile range. Wilcoxon matched-pairs signed test was used to analyze differences between CD161^−^ and CD161^+^ cell populations. Kruskal–Wallis test with Dunn’s multiple comparison tests were used to analyze differences between three or more groups. *P*-values below 0.05 were considered significant and are shown in graphs as *P < 0.05, **P < 0.01, and ***P < 0.001.

## Results

### CD161^+^ Treg Produce Pro-inflammatory Cytokines, despite Classical Treg Phenotype

**Figure 1:**
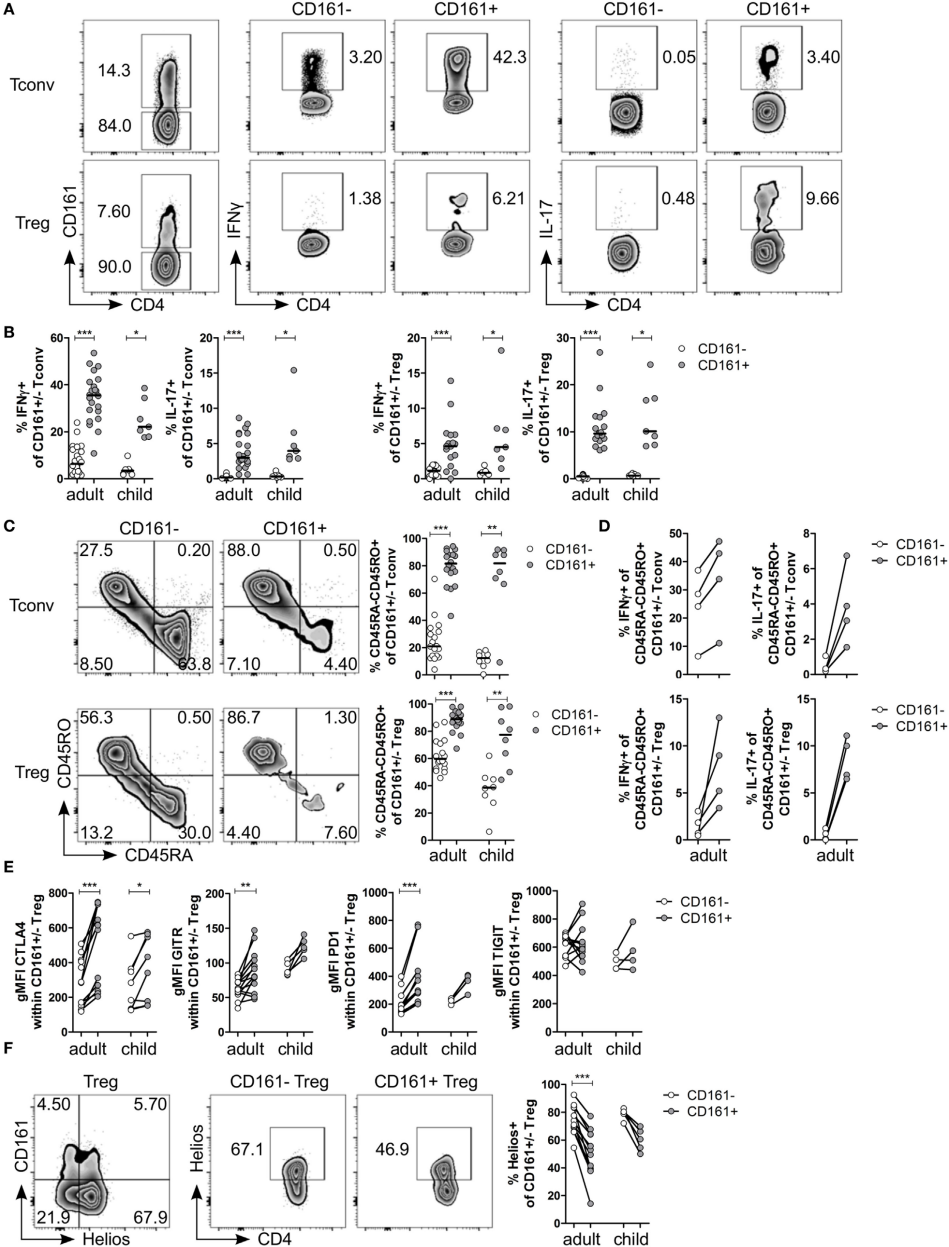
**CD161^+^ regulatory T cells (Treg) produce pro-inflammatory cytokines and express markers typically associated with a Treg phenotype**. CD127^+^CD25^−^ conventional T cells (Tconv) and CD127^low^CD25^hi^Foxp3^+^ (Treg) (gated as in Figure S1 in Supplementary Material) from healthy control peripheral blood were analyzed by flow cytometry. **(A)** Left plots show gating of CD161^+^ and CD161^−^ Tconv and Treg. Representative plots depicting interferon (IFN)-γ^+^ and interleukin (IL)-17^+^ cells within CD161^−^ and CD161^+^ Tconv and Treg populations. **(B)** Summary graphs showing percentage IFNγ^+^ and IL-17^+^ within CD161^−^ (○) and CD161^+^ (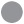) Tconv and Treg from healthy adults (*n* = 17–22) and children (*n* = 7). **(C)** Representative plots and summary graphs showing percentage memory cells, defined as CD45RA^−^CD45RO^+^ cells, within CD161^−^ (○) and CD161^+^ (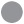) Tconv and Treg in healthy adults (*n* = 18) and children (*n* = 8). **(D)** Summary graphs showing percentage IFNγ^+^ and IL-17^+^ within CD45RA^−^CD45RO^+^ CD161^−^ (○) and CD161^+^ (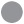) Tconv and Treg from healthy adults (*n* = 4). **(E)** Protein expression of CTLA4, GITR, PD1, and TIGIT within CD161^−^ (○) and CD161^+^ (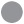) Treg from healthy adults (*n* = 12–13) and children (*n* = 4–8). **(F)** Co-staining of CD161 and Helios, and Helios^+^ cells within CD161^−^ and CD161^+^ Treg. Summary graph showing percentage Helios^+^ within CD161^−^ (○) and CD161^+^ (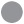) Treg from healthy adults (*n* = 12) and children (*n* = 5). Statistical significance: *P < 0.05, **P < 0.01, ***P < 0.001.

Analysis of Tconv and Treg expressing CD161 for cytokine production confirmed that CD161^+^ Tconv and CD161^+^ Treg from peripheral blood produced significantly higher levels of IFNγ and IL-17 compared to CD161^−^ counterparts (Figures 1A,B; Figure S1 in Supplementary Material: gating strategy). Despite the significantly increased proportions of memory cells (defined as CD45RA^−^CD45RO^+^ cells) in both CD161^+^ Tconv and CD161^+^ Treg (Figure 1C), comparison of cytokine production within memory CD161^+^ Tconv and Treg revealed a non-significant trend towards more IFNγ and IL-17 production by memory CD161^+^ Tconv and memory CD161^+^ Treg compared to their CD161^−^ counterparts within the four samples analyzed (Figure 1D). These data suggest that differences in cytokine production between CD161^+^ and CD161^−^ cells might not solely be attributed to differences in memory phenotype.

Despite the pro-inflammatory phenotype typically associated with effector T cells, CD161^+^ Treg from healthy controls also exhibited a classical Treg phenotype. CD161^+^ Treg expressed significantly higher levels of CTLA4, GITR, and PD1 compared to CD161^−^ Treg in blood of healthy adults (Figure 1E; Figures S2A–C in Supplementary Material). Within blood of healthy children, only CTLA4 protein expression was significantly higher within CD161^+^ Treg compared to CD161^−^ Treg. We observed no significant difference in protein expression of TIGIT (Figure 1E; Figure S2D in Supplementary Material), a marker identifying a suppressive Treg population that also shares features with effector T cells (36, 37). Interestingly, CD161^+^ Tconv also expressed higher levels of CTLA4, GITR, PD1, and TIGIT compared to CD161^−^ Tconv (Figures S2A–D in Supplementary Material). Given that there is evidence that Helios^low/−^ Treg have the capacity to make cytokines (38, 39), we analyzed Helios expression within CD161^+^ and CD161^−^ Treg. Although CD161^+^ and Helios^low/−^ cells were not entirely overlapping populations, we observed significantly fewer Helios^+^ cells within CD161^+^ Treg compared to CD161^−^ Treg within blood of healthy adults (Figure 1F). This is in accordance with the described cytokine-producing phenotype of Helios^low/−^ Treg and CD161^+^ Treg.

### Transcriptome Analysis Reveals a Shared Transcriptional Signature between CD161^+^ Tconv and CD161^+^ Treg

**Figure 2:**
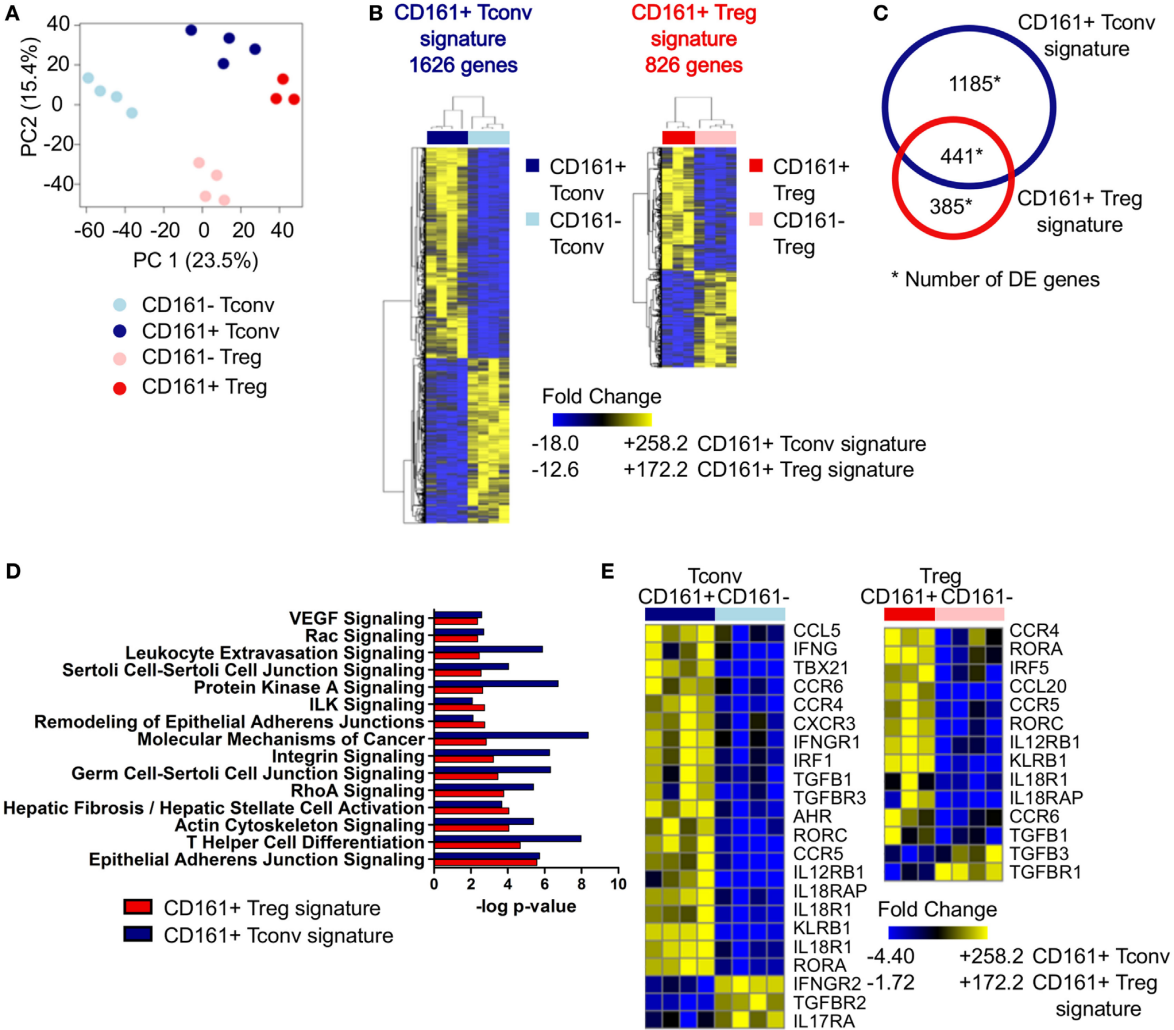

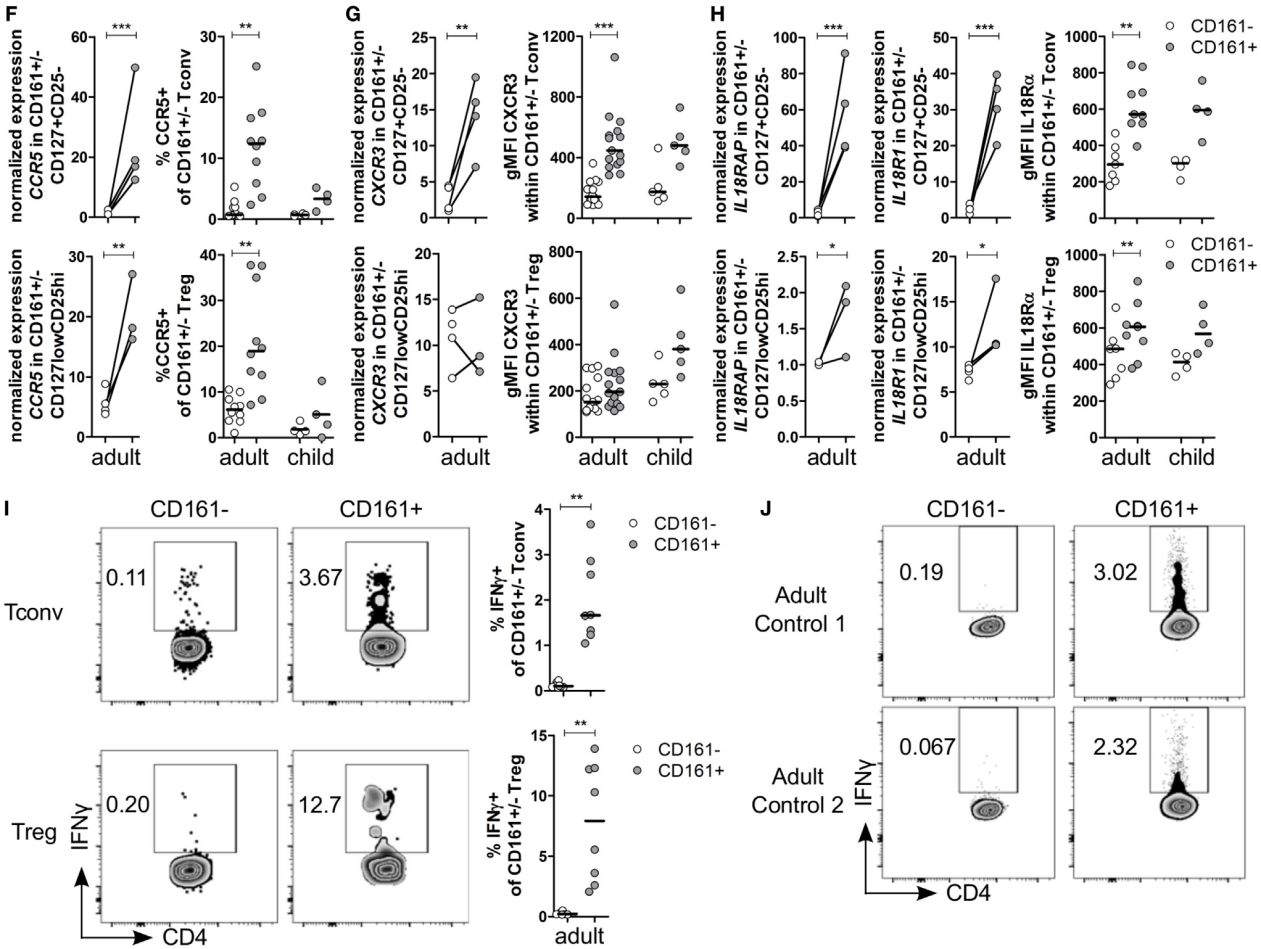
**Shared canonical signature between CD161^+^ conventional T cells (Tconv) and CD161^+^ regulatory T cells (Treg) reveals a T helper (Th) phenotype of CD161^+^ T cells**. Healthy adult peripheral blood mononuclear cells (PBMC) (*n* = 4) were sorted into CD4^+^CD127^+^CD25^−^CD161^+^ (CD161^+^ Tconv) or CD161^−^ (CD161^−^ Tconv), and CD4^+^CD127^low^CD25^hi^CD161^+^ (CD161^+^ Treg) or CD161^−^ (CD161^−^ Treg) for RNAseq analysis. **(A)** Principal component analysis (PCA) of data from all samples before applying filters to assess differentially expressed (DE) genes. One CD161^+^ Treg sample was excluded from analysis due to low cDNA yield. **(B)** Heat maps of DE genes comparing CD161^+^ and CD161^−^ Tconv (CD161^+^ Tconv signature) (1626 DE genes, left), and CD161^+^ and CD161^−^ Treg (CD161^+^ Treg signature) (826 DE genes, right). **(C)** Venn diagram representing overlap in transcriptional signatures of CD161^+^ Tconv and CD161^+^ Treg. **(D)** Canonical pathways altered in both CD161^+^ Tconv (blue bars) and CD161^+^ Treg (red bars) signatures identified using ingenuity pathway analysis with −log *P*-value >2 (equals *P*-value <0.01). **(E)** DE genes associated with Th1 and Th17 in CD161^+^ Tconv and CD161^+^ Treg compared to CD161^−^ counterparts. **(F)** Analysis of CCR5 expression; normalized expression values (left, from RNAseq) and percentage CCR5^+^ cells (right, flow cytometry) within CD161^−^ (○) and CD161^+^ (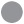) Tconv and Treg in healthy adult (*n* = 10) and child (*n* = 4) PBMC. **(G,H)** Analysis of CXCR3 **(G)** and IL18R **(H)** expression; normalized expression values (left, from RNAseq) and protein expression (right, flow cytometry) within CD161^−^ (○) and CD161^+^ (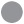) Tconv and Treg in healthy adult (*n* = 9–15) and child (*n* = 4–5) PBMC. **(I,J)** PBMC **(I)** or sorted CD161^+^ and CD161^−^ Tconv **(J)** from healthy adults were cultured in the presence of 50 ng/ml IL-18 and IL-12 for 24 h with Brefeldin A for last 4 h of culture. **(I)** Representative plots and summary graphs showing percentage IFNγ^+^ within CD161^−^ (○) and CD161^+^ (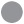) Tconv and Treg (*n* = 8). **(J)** Representative plots showing IFNγ^+^ cells within sorted CD161^+^ and CD161^−^ Tconv from 2 healthy adult controls. Statistical significance: *P < 0.05, **P < 0.01, ***P < 0.001.

To investigate the effector-like phenotype of CD161^+^ T cells, CD161^+^ and CD161^−^ Tconv and Treg were sorted (Figures S3A,B in Supplementary Material) and their transcriptome was analyzed. PCA revealed that CD161^+^ and CD161^−^ T cells represent distinct populations within Tconv or Treg lineages. In addition, samples clustered by cell population before applying filters to examine DE genes (Figure 2A). Analysis of CD161^+^ and CD161^−^ Tconv indicated 1626 DE genes (CD161^+^ Tconv signature; Table S2 in Supplementary Material), and analysis of CD161^+^ and CD161^−^ Treg indicated 826 DE genes (CD161^+^ Treg signature; Table S3 in Supplementary Material) (Figure 2B). Comparison of CD161^+^ Tconv and CD161^+^ Treg signatures revealed a shared transcriptional profile between CD161^+^ T cells (Figure 2C). Pathway analysis of CD161^+^ Tconv and CD161^+^ Treg signatures (Tables S4 and S5 in Supplementary Material, respectively) showed that similar pathways were altered in both signatures (Figure 2D). Among the over-represented pathways were the T helper differentiation pathway and pathways associated with cell migration, including epithelial adherens juction, actin cytoskeleton signaling, integrin signaling, and rho signaling, suggesting that CD161^+^ T cells might be more migratory than their CD161^−^ counterparts.

### CD161^+^ Tconv and CD161^+^ Treg Exhibit a T Helper Phenotype and Can Respond in a TCR-Independent Manner

Many differentially expressed genes were part of the T helper differentiation pathway including those specifically associated with Th1 and Th17 cells. CD161^+^ Tconv showed significantly higher expression of *TBX21* and *IRF5* (Th1 transcription factors), and *RORC* and *RORA* (Th17 transcription factors) were significantly higher in both CD161^+^ Tconv and CD161^+^ Treg compared to their CD161^−^ counterparts. *AHR* expression was only significantly higher in CD161^+^ Tconv compared to CD161^−^ Tconv (Figure 2E; Figure S4A in Supplementary Material). Interestingly, *GATA3* expression was also higher in CD161^+^ Tconv and CD161^+^ Treg, and *BCL6* was higher in CD161^+^ Tconv, but not CD161^+^ Treg, compared to CD161^−^ populations (Figure S4A in Supplementary Material). CD161^+^ Tconv showed significantly higher expression of *IFNG* compared to CD161^−^ Tconv (Figure 2E; Figure S4B in Supplementary Material), but expression levels of other cytokine transcripts, including *IL4, IL10, IL13, IL21*, and *IL22*, showed no significant differences in expression between CD161^+^ and CD161^−^ populations (data not shown).

Furthermore, expression of chemokine receptors associated with migration of Th cells, including *CCR6* (Th17) (Figure 2E; Figure S6A in Supplementary Material), *CCR5*, and *CXCR3* (both Th1), was higher in CD161^+^ Tconv and CD161^+^ Treg (Figures 2E–G). We and others have previously demonstrated high levels of CCR6, RORC, and T-bet in CD161^+^ T populations (14, 26, 29). Here, we report an increase in percentage CCR5^+^ cells in both CD161^+^ Tconv and CD161^+^ Treg (Figure 2F) and higher protein expression of CXCR3 (Figure 2G) in CD161^+^ Tconv compared to CD161^−^ counterparts.

CD161^+^ Tconv and CD161^+^ Treg also expressed higher levels of *IL12RB1, IL18RAP, IL18R1*, and IL-18Rα protein (Figures 2E and H). These receptors have been previously reported to be expressed on innate-like lymphocytes such as mucosal-associated invariant T (MAIT) cells (40) and ILC (28), and mediate rapid effector functions in response to IL-12 and IL-18. Therefore, we investigated the responsiveness of CD161^+^ Tconv and CD161^+^ Treg to IL-12 and IL-18 by culturing PBMC from healthy adults in presence of IL-12 and IL-18, but without a TCR signal, for 24 h. Both CD161^+^ Tconv and CD161^+^ Treg produced more IFNγ in response to IL-12 and IL-18 compared to CD161^−^ cells (Figure 2I). In addition, sorted CD161^+^ and CD161^−^ Tconv were cultured alone in IL-12 and IL-18 in absence of a TCR signal. Only CD161^+^ Tconv produced IFNγ in response to IL-12 and IL-18 (Figure 2J) suggesting that CD161^+^ T cells may have innate-like characteristics and can respond in a TCR-independent manner.

### CD161^+^ T Cells Have a Proliferative Phenotype

**Figure 3:**
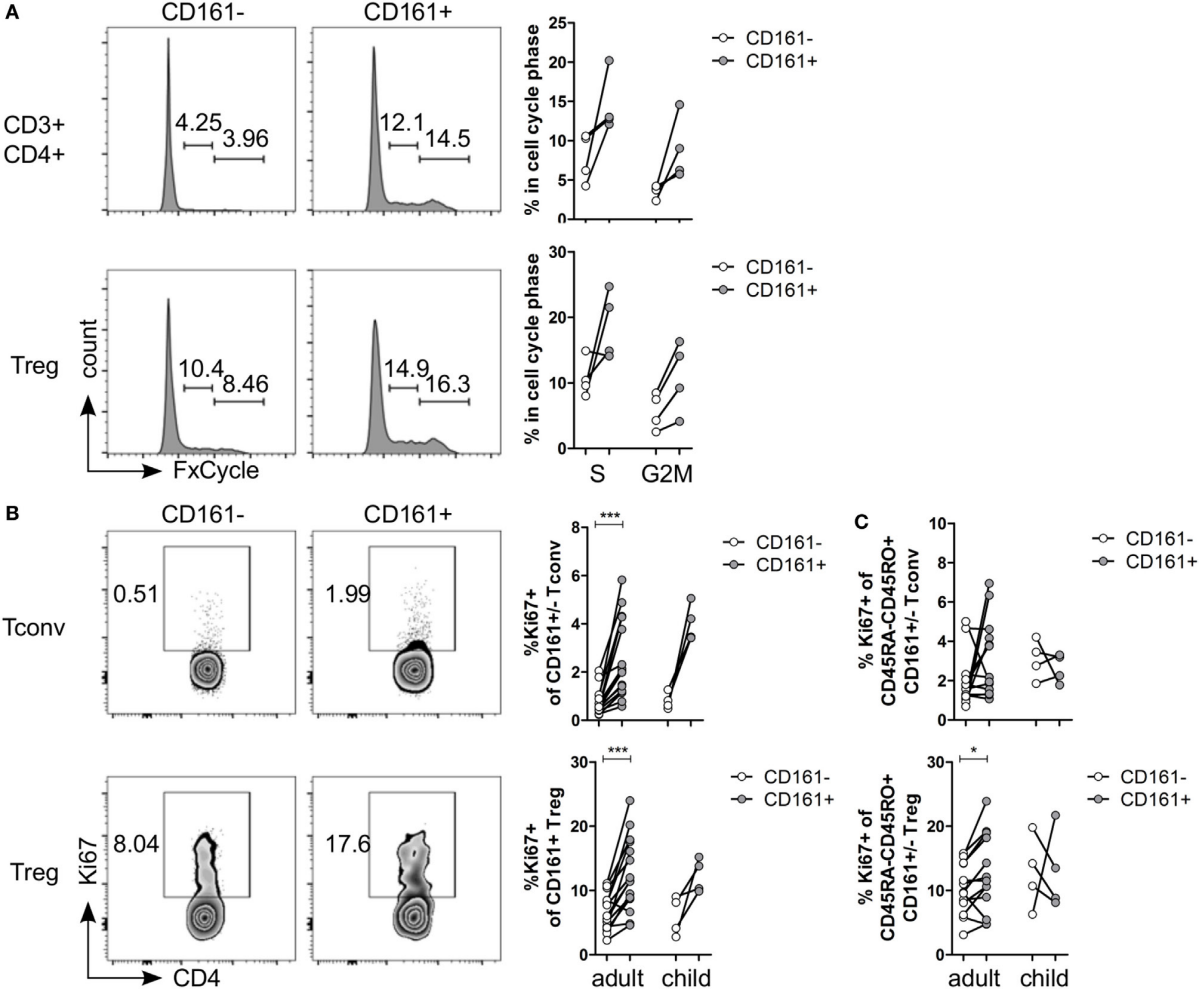
**CD161^+^ T cells have a proliferative phenotype**. **(A)** Healthy adult peripheral blood mononuclear cells (PBMC) were stimulated with 1 µg/ml αCD3 + 5 µg/ml αCD28 for 4 days and analyzed for cell cycle profile by flow cytometry using FxCycle Violet. Representative histograms and summary graphs indicating percentage of cells in S and G2M phase within CD161^−^ (○) and CD161^+^ (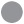) CD3^+^CD4^+^ T cells and Treg (*n* = 4). **(B)** PBMC from healthy adults and children were analyzed *ex vivo* for expression of Ki67. Representative plots and summary graphs showing percentage Ki67^+^ cells within CD161^−^ (○) and CD161^+^ (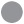) Tconv and Treg from healthy adults (*n* = 16) and children (*n* = 4). **(C)** Percentage Ki67^+^ within memory cells, defined as CD45RA^−^CD45RO^+^ cells, gated on CD161^−^ (○) and CD161^+^ (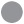) Tconv and Treg in healthy adults (*n* = 14) and children (*n* = 4). Statistical significance: *P < 0.05, ***P < 0.001.

Several functional pathways that were over-represented in CD161^+^ Treg were associated with cell cycle progression (Table S5 in Supplementary Material). *In vitro* analysis of cell cycle profile showed a non-significant trend towards more CD161^+^ T cells in S and G2M phases compared to CD161^−^ T cells (Figure 3A). Consistent with this observation, the percentage of Ki67^+^ cells within CD161^+^ Tconv and CD161^+^ Treg was significantly higher compared to CD161^−^ cells within blood of healthy adults, but this trend did not reach statistical significance in the four healthy children analyzed (Figure 3B). Together, these data suggest that CD161^+^ T cells have a high proliferative turnover, which indicates that these cells may have recently encountered their cognate antigen.

The proliferative phenotype of CD161^+^ Treg could be confounded by the high proportion of memory cells within CD161^+^ Treg (Figure 1C) as CD45RO-expressing CD4^+^CD25^hi^Foxp3^+^ cells are highly proliferative (41, 42). Analysis of Ki67^+^ cells within memory cells still showed significantly more Ki67^+^ cells within memory CD161^+^ Treg, but not within memory CD161^+^ Tconv, compared to CD161^−^ counterparts in healthy adults (Figure 3C) indicating that the high proliferative state of CD161^+^ Treg is not accounted for solely by its memory phenotype.

### CD161^+^ T Cells Express Markers Associated with Tissue Homing

**Figure 4:**
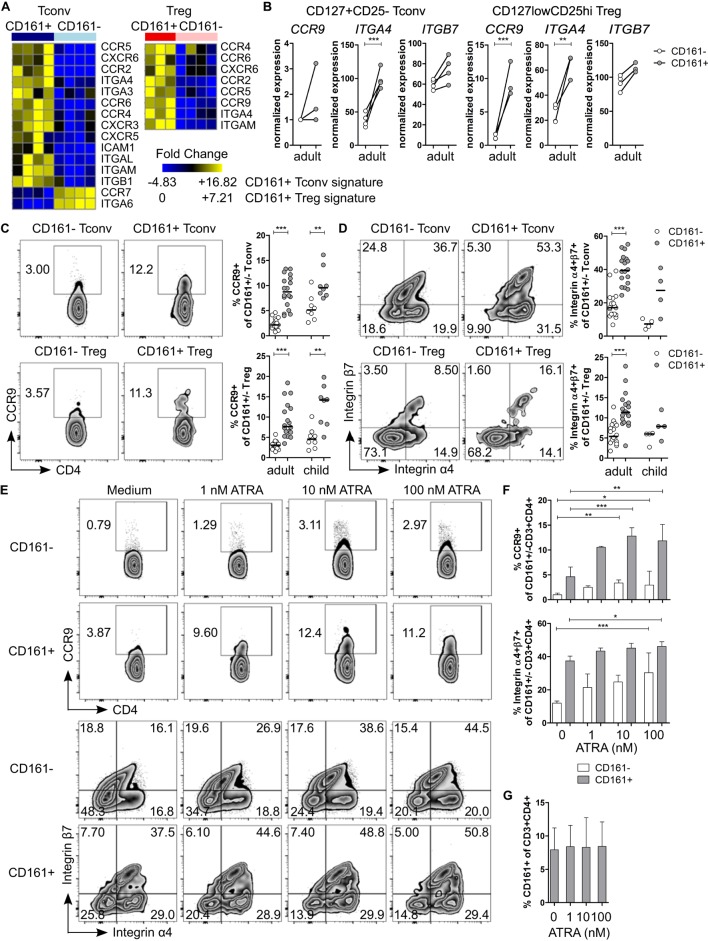

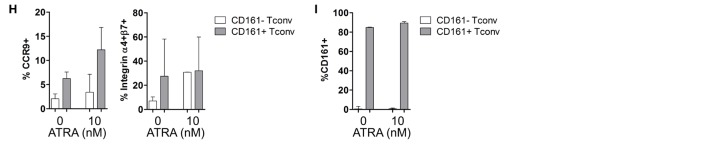
**CD161^+^ T cells express markers associated with gut homing and respond to all-trans retinoic acid (ATRA)**. **(A)** Heat maps showing DE genes encoding chemokine receptors and integrins in CD161^+^ conventional T cells (Tconv) and CD161^+^ regulatory T cells (Treg) compared to their CD161^−^ counterparts. **(B)** Normalized expression values for *CCR9, ITGA4*, and *ITGB7* within CD161^−^ (○) and CD161^+^ (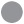) Tconv and Treg from RNAseq. **(C,D)** Representative plots and summary graphs showing percentage CCR9^+^ **(C)** and integrin α4^+^β7^+^ **(D)** cells by flow cytometry within CD161^−^ (○) and CD161^+^ (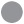) Tconv and Treg in healthy adults (*n* = 18) and children (*n* = 4–8). **(E–G)** Healthy adult peripheral blood mononuclear cells (PBMC) were cultured in medium, 1, 10, or 100 nM ATRA for 4 days as indicated and analyzed for expression of CCR9, integrin α4β7 and CD161 (*n* = 6). **(E)** Representative plots showing percentage CCR9^+^ and integrin α4^+^β7^+^ cells within CD161^+^ and CD161^−^ CD3^+^CD4^+^ T cells at culture conditions indicated. **(F)** Summary graphs showing percentage CCR9^+^ and integrin α4^+^β7^+^ cells within CD161^−^ (○) and CD161^+^ (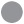) CD3^+^CD4^+^ T cells at culture conditions indicated. **(G)** Percentage CD161^+^ cells within CD3^+^CD4^+^ cells at end of culture. **(H,I)** Purified CD161^+^ and CD161^−^ Tconv from healthy adults were cultured with ATRA as described before (*n* = 3). **(H)** Summary graphs showing percentage CCR9^+^ and integrin α4^+^β7^+^ cells within sorted CD161^−^ (○) and CD161^+^ (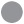) Tconv at culture conditions indicated. **(I)** Percentage CD161^+^ cells of CD3^+^CD4^+^ cells within sorted CD161^−^ (○) and CD161^+^ (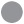) Tconv at end of culture. Statistical significance: *P < 0.05, **P < 0.01, ***P < 0.001.

Pathway analysis suggested that CD161^+^ Tconv and CD161^+^ Treg might be more migratory than CD161^−^ populations. Therefore, we considered their ability to migrate between different tissues. Interestingly, we observed that transcripts encoding the gut-homing receptors *CCR9* and *ITGA4* (integrin α4β7 is associated with gut homing) (43, 44) were upregulated in CD161^+^ Tconv and CD161^+^ Treg (Figures 4A,B). Analysis of CCR9 and integrin α4β7 showed significantly higher percentages of CCR9^+^ (Figure 4C) and integrin α4^+^β7^+^ (Figure 4D) cells within CD161^+^ Tconv and CD161^+^ Treg compared to CD161^−^ populations in blood from healthy adults. In healthy children (*n* = 4-8), percentage CCR9^+^ cells was significantly higher within CD161^+^ Tconv and CD161^+^ Treg (Figure 4C) compared to CD161^−^ cells, whereas the trend towards higher percentage integrin α4^+^β7^+^ cells within CD161^+^ Tconv and CD161^+^ Treg was not significant (Figure 4D).

Retinoic acid (RA), the active form of vitamin A, plays an important role in T cell-mediated immune responses and is known to induce gut homing of lymphocytes (45). Since both *CCR9* and *ITGA4* are direct targets of RA (46, 47), we hypothesized that CD161^+^ T cells express higher levels of CCR9 and integrin α4β7 because they have been exposed to a RA signal during T cell differentiation, whereas CD161^−^ T cells have not been previously exposed. To determine whether CD161^−^ T cells can upregulate CCR9 and integrin α4β7, PBMC from healthy adults were activated in presence of all-trans retinoic acid (ATRA), the predominant biological form of RA. Activation of PBMC in presence of ATRA and a TCR signal did not significantly alter percentage CCR9^+^ cells, whereas percentage integrin α4^+^β7^+^ cells was significantly higher within both CD161^+^ and CD161^−^ CD3^+^CD4^+^ cells, but no difference was found between CD161^+^ and CD161^−^ cells (Figures S5A,B in Supplementary Material). Previous reports suggested that ATRA-induced CCR9 expression on T cells requires an initial TCR signal (46). To address the requirement of a TCR signal, PBMC were exposed to ATRA alone. Both CD161^+^ and CD161^−^ CD3^+^CD4^+^ cells significantly upregulated CCR9 and integrin α4β7, although CD161^+^ cells contained significantly more CCR9^+^ and integrin α4^+^β7^+^ cells than CD161^−^ cells (*P* < 0.05; significant difference between CD161^+^ and CD161^−^ cells is not shown in Figure 4F) (Figures 4E,F). One explanation for the upregulation of CCR9 and integrin α4β7 on CD161^−^ CD3^+^CD4^+^ cells after exposure to ATRA could be that ATRA alters CD161 protein expression itself. However, we did not observe significant changes in percentage CD161^+^ CD3^+^CD4^+^ cells in response to ATRA + TCR (Figure S5C in Supplementary Material) or ATRA alone (Figure 4G). To exclude any potential effects of other cell populations within PBMC, sorted CD161^+^ and CD161^−^ Tconv from healthy adults were exposed to ATRA with or without TCR stimulation. We observed similar results for expression of CCR9 and integrin α4β7 when cells were exposed to ATRA + TCR (Figure S5D in Supplementary Material) or ATRA alone (Figure 4H) compared to PBMC cultures. In addition, exposure to ATRA did not alter CD161 protein expression within sorted cell populations (Figure S5E in Supplementary Material: ATRA + TCR; Figure 4I: ATRA alone).

We also analyzed expression of chemokine receptors and integrins, which mediate homing to other tissues within the RNAseq dataset. Indeed, both CD161^+^ Tconv and CD161^+^ Treg expressed significantly higher levels of *CCR2, CCR4, CCR5, CCR6*, and *CXCR6* (Figures 2E,F and 4A; Figure S6A in Supplementary Material). Furthermore, CD161^+^ Tconv expressed significantly lower levels of *CCR7*, but significantly higher levels of *CXCR3* and *CXCR5* (Figures 2E,G and 4A; Figure S6A in Supplementary Material). In addition, CD161^+^ Tconv expressed significantly higher levels of *ITGA3, ITGA6, ITGAL, ITGAM, ITGB1*, and *ICAM*, whereas CD161^+^ Treg only expressed significantly higher levels of *ITGAM* (Figure S6B in Supplementary Material). Expression of CCR4, CCR5, CXCR3, CXCR6, and integrin α4β1 has previously been associated with T cell homing to specific tissues including lung, skin, liver, and heart (48, 49).

### Limited Overlap in TCRβ Repertoire between CD161^+^ and CD161^−^ Tconv and Treg

**Figure 5:**
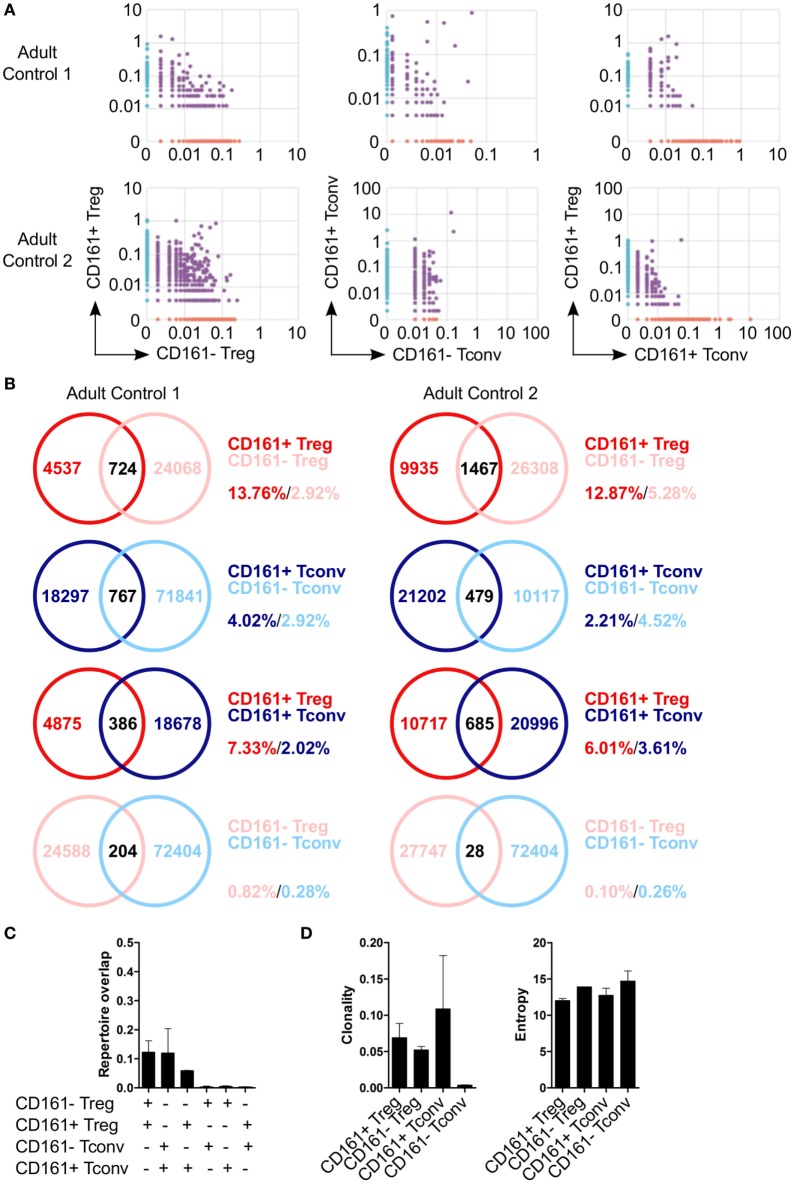
**Limited overlap in TCRβ repertoire between CD161^+^ and CD161^−^ conventional T cells (Tconv) and regulatory T cells (Treg) from peripheral blood**. Sorted CD161^+^ and CD161^−^ Tconv and Treg from two healthy adults were analyzed for TCRβ repertoire by TCRB CDR3 VDJ sequence analysis. **(A)** Pair-wise comparison plots showing average productive frequency of shared (purple) and unique clones (on *x*- or *y*-axis) at nucleotide level between the different cell populations as indicated. **(B)** Venn diagrams showing number of shared and unique TCR sequences between the cell populations as indicated. Frequencies indicate percentage of unique sequences shared between the two repertoires divided by total sequences for that cell population. **(C)** Summary graph depicting repertoire overlap between different cell populations and was calculated as followed: shared sequences by samples A and B divided by total sequences in samples A and B. **(D)** Summary graphs for clonality and entropy (Shannon entropy) of the sorted cell populations.

Because of the shared transcriptional signatures between CD161^+^ Tconv and CD161^+^ Treg, we investigated whether CD161^+^ Tconv and CD161^+^ Treg originated from the same precursor cell. Analysis of TCRβ repertoire showed very little overlap between CD161^+^ and CD161^−^ Tconv, and CD161^+^ and CD161^−^ Treg. In addition, there was limited overlap between CD161^+^ Tconv and CD161^+^ Treg indicating that these populations most likely do not originate from the same precursor cell (Figures 5A–C). Clonality of CD161^+^ Tconv, CD161^+^ Treg, and CD161^−^ Treg was similar to the median clonality (0.075) of the adult TCR repertoire in blood, whereas clonality for CD161^−^ Tconv was much lower. The Shannon entropy score (normalized measure of diversity of the rearrangements) was similar for all populations (Figure 5D).

### Enrichment of CD161^+^ T Cells at the Inflamed Site, but CD161+ T Cells from the Inflamed Site Show Lower Expression of Gut-Homing Markers

**Figure 6:**
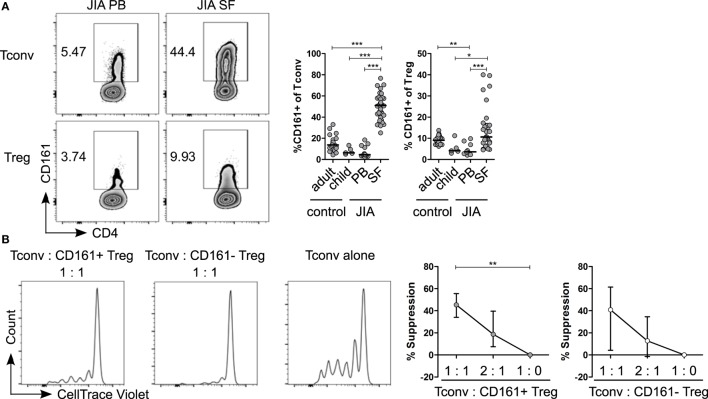
**CD161^+^ regulatory T cells (Treg) from the inflamed site are suppressive in an *in vitro* suppression assay**. **(A)** Representative plots showing percentage CD161^+^ cells within conventional T cells (Tconv) and Treg from paired juvenile idiopathic arthritis (JIA) peripheral blood (PB) and synovial fluid (SF). Summary graphs showing percentage CD161^+^ cells within Tconv and Treg in healthy adults (*n* = 25) and children (*n* = 5), JIA PB (*n* = 13) and JIA SF (*n* = 27). **(B)** CD161^+^ and CD161^−^ Treg were sorted from JIA synovial fluid mononuclear cells (SFMC) and cocultured with Tconv labeled with CellTrace Violet at Tconv:Treg ratio of 1:0, 1:1, and 2:1 in presence of plate-bound αCD3 (1 µg/ml) and αCD28 (5 µg/ml) for 4 or 5 days. Representative histograms showing CellTrace Violet dilution within CD4^+^ T cells in presence of CD161^+^ (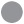) or CD161^−^ (○) at 1:1 ratio or Tconv alone (1:0). Summary graphs showing percentage suppression by CD161^+^ and CD161^−^ Treg at 1:0 (*n* = 5), 1:1 (*n* = 5), and 2:1 ratio (*n* = 4). Statistical significance: *P < 0.05, **P < 0.01, ***P < 0.001.

Given the pro-inflammatory phenotype of CD161^+^ T cells, we sought to address the relevance of these cells in inflammation. We analyzed the frequency of CD161^+^ Tconv and CD161^+^ Treg in childhood autoimmune arthritis, JIA, and found that CD161^+^ Tconv and CD161^+^ Treg were significantly enriched in JIA SF compared to JIA and HC PB (Figure 6A). The cytokine-producing phenotype of CD161^+^ T cells was also maintained in JIA PB and SF (Figures S7A,B in Supplementary Material). Functionally, both CD161^+^ and CD161^−^ Treg from JIA SF were able to suppress proliferation of Tconv cells in an *in vitro* suppression assay. There was no significant difference in suppressive capacity between the CD161^+^ and CD161^−^ Treg populations (Figure 6B).

**Figure 7:**
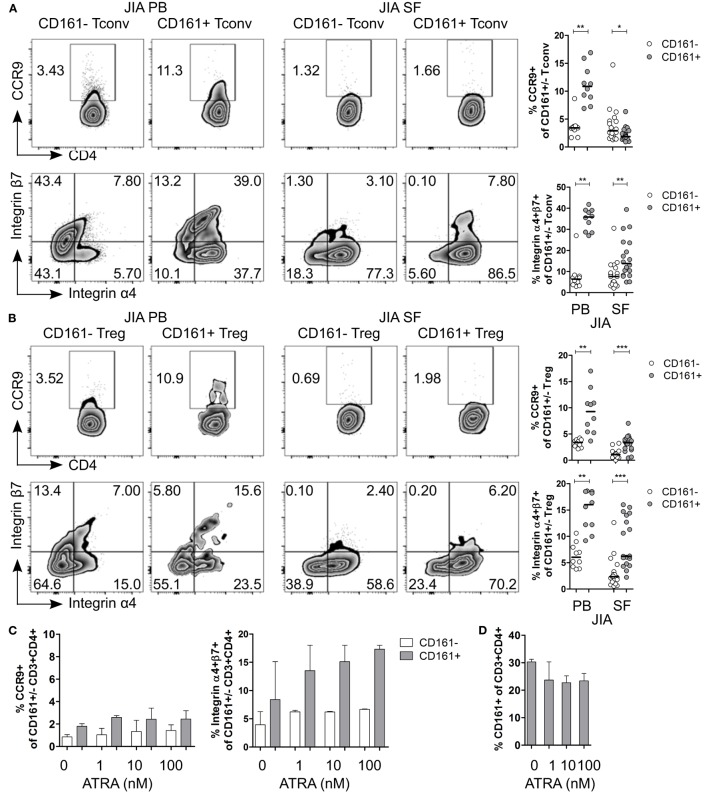
**CD161^+^ conventional T cells (Tconv) and CD161^+^ regulatory T cells (Treg) from the inflamed site show lower expression of gut-homing receptors**. **(A,B)** Representative plots and summary graphs showing percentage CCR9^+^ and integrin α4^+^β7^+^ cells within CD161^−^ (○) and CD161^+^ (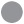) Tconv **(A)** and Treg **(B)** in juvenile idiopathic arthritis (JIA) peripheral blood (PB) (*n* = 10) and JIA synovial fluid (SF) (*n* = 18). **(C,D)** Synovial fluid mononuclear cells (SFMC) were cultured in presence of ATRA as described before (*n* = 3). **(C)** Representative plots and summary graphs showing percentage CCR9^+^ and integrin α4^+^β7^+^ cells within CD161^−^ (○) and CD161^+^ (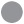) CD3^+^CD4^+^ T cells at culture conditions indicated. **(D)** Percentage CD161^+^ cells within CD3^+^CD4^+^ T cells at end of culture. Statistical significance: *P < 0.05, **P < 0.01, ***P < 0.001.

Given the observed expression of CCR9 and α4β7 on CD161^+^ Tconv and CD161^+^ Treg in blood, and the suggested link between arthritis and gut inflammation (50–57), we next examined the expression of these receptors on CD161^+^ Tconv and CD161^+^ Treg from patients with autoimmune arthritis. *Ex vivo* analysis showed significantly higher percentages of CCR9^+^ and integrin α4^+^β7^+^ cells within CD161^+^ Tconv (Figure 7A) and CD161^+^ Treg (Figure 7B) compared to their CD161^−^ counterparts in blood of JIA patients. However, in SF, CD161^+^ Tconv contained significantly fewer CCR9^+^ cells compared to CD161^−^ Tconv, and percentage CCR9^+^ cells within both CD161^+^ Tconv and CD161^+^ Treg was reduced in JIA SF compared to JIA PB (Figures 7A,B). The proportion of integrin α4^+^β7^+^ cells in CD161^+^ Tconv and CD161^+^ Treg compared to CD161^−^ populations was still significantly higher in JIA SF (Figures 7A,B), although, the proportions of integrin α4^+^β7^+^ cells within both CD161^+^ Tconv (Figure 7A) and CD161^+^ Treg (Figure 7B) were also lower in JIA SF compared to JIA PB. To determine whether cells from SF can upregulate expression of CCR9 and integrin α4β7, SFMC from JIA patients were exposed to ATRA in presence or absence of a TCR signal. We observed no change in percentage CCR9^+^ cells within CD161^+^ and CD161^−^ CD3^+^CD4^+^ cells upon activation of SFMC in presence of ATRA plus a TCR signal, whereas percentage integrin α4^+^β7^+^ was increased within both CD161^+^ and CD161^−^ CD3^+^CD4^+^ cells (Figure S5F in Supplementary Material). Percentage CD161^+^ CD3^+^CD4^+^ cells did not significantly change in response to ATRA + TCR (Figure S5G in Supplementary Material). Exposure to ATRA alone also did not increase percentage CCR9^+^ cells within CD161^+^ and CD161^−^ CD3^+^CD4^+^ cells. Percentage integrin α4^+^β7^+^ cells within CD161^+^ CD3^+^CD4^+^ cells might be slightly increased with addition of ATRA, whereas there was no change in percentage integrin α4^+^β7^+^ cells within CD161^−^ CD3^+^CD4^+^ cells (Figure 7C). There was no significant change in percentage CD161^+^ CD3^+^CD4^+^ cells during ATRA treatment (Figure 7D). RA can be synthesized in response to inflammation (58–61), but the lower levels of CCR9^+^ and integrin α4^+^β7^+^ cells in JIA SF suggest prior imprinting by RA in the gut or at peripheral sites rather than during synovial inflammation.

### Overlap in TCRβ Repertoire between CD161^+^ and CD161^−^ Tconv and Treg from the Inflamed Site

**Figure 8:**
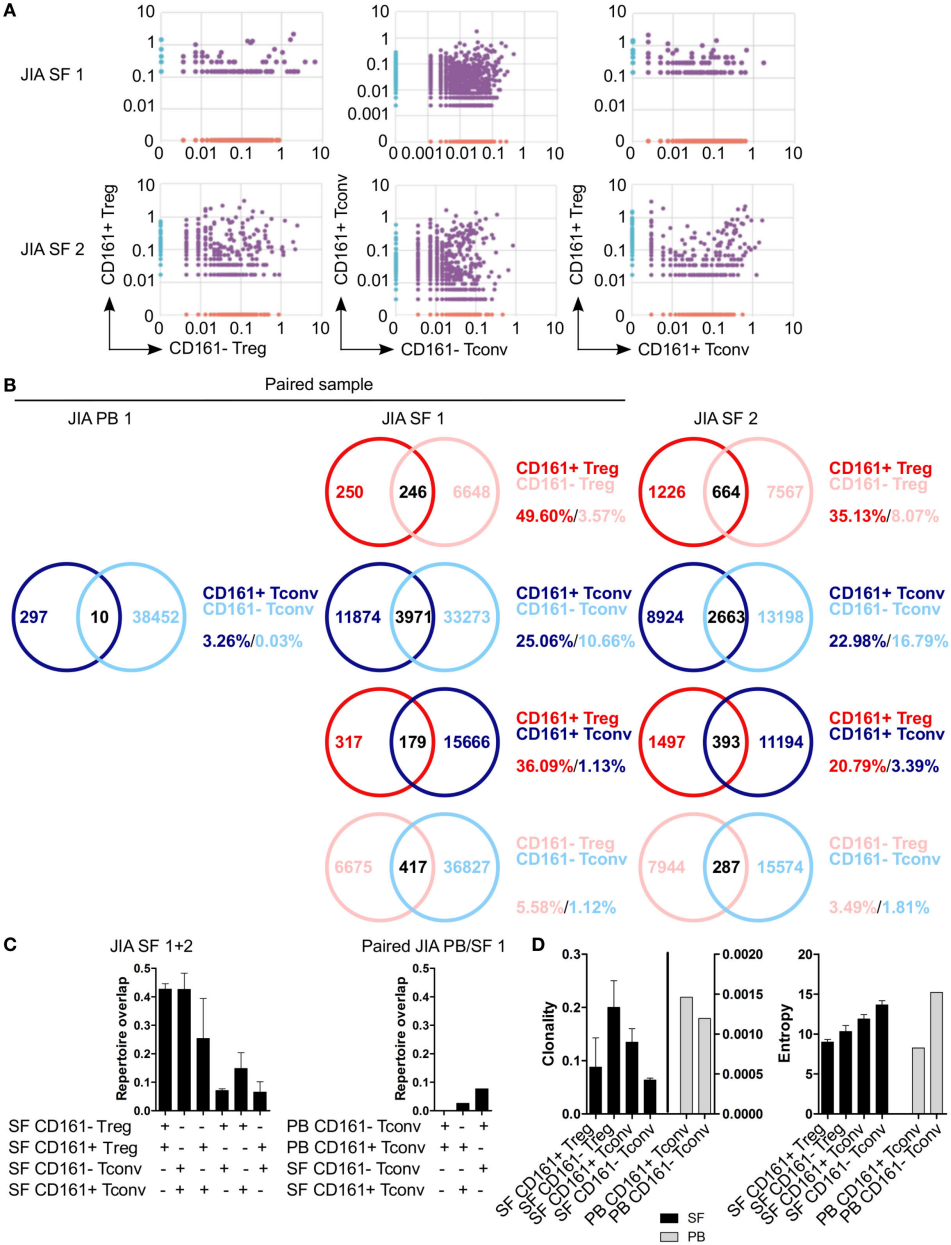
**CD161^+^ and CD161^−^ conventional T cells (Tconv) and regulatory T cells (Treg) from the inflamed site show sharing of TCRβ repertoires**. Purified CD161^+^ and CD161^−^ Tconv and Treg from juvenile idiopathic arthritis (JIA) synovial fluid (SF) (*n* = 2) and CD161^+^ and CD161^−^ Tconv from JIA peripheral blood (PB) (*n* = 1) were analyzed for TCRβ repertoire. Sample JIA PB/SF 1 is a paired sample whereas JIA SF 2 is not. **(A)** Pair-wise comparison plots showing average productive frequency of shared (purple) and unique clones (on *x*- or *y*-axis) between the different cell populations from JIA SF as indicated. **(B)** Venn diagrams showing number of shared and unique TCR sequences between the cell populations from JIA PB/SF as indicated. Frequencies indicate percentage of unique sequences shared between the two repertoires divided by total sequences for that cell population. **(C)** Summary graphs depicting repertoire overlap (calculated as followed: shared sequences by samples A and B divided by total sequences in samples A and B) between indicated cell populations from JIA SF only (left, *n* = 2), and CD161^−^ and CD161^+^ Tconv from paired JIA PB/SF 1 (right, *n* = 1). **(D)** Summary graphs for clonality and entropy (Shannon entropy) for cell populations from JIA SF (black bars, *n* = 2) and JIA PB (gray bars, *n* = 1).

To determine whether CD161^+^ and CD161^−^ Tconv and Treg from the site of inflammation in JIA patients also represent distinct cell populations as observed in blood, we analyzed their TCRβ repertoire. Interestingly, there was considerable overlap in TCRβ repertoire between both CD161^+^ and CD161^−^ Tconv, and CD161^+^ and CD161^−^ Treg from JIA SF. In addition, there was some degree of overlap (~20%) between CD161^+^ Tconv and CD161^+^ Treg from JIA SF (Figures 8A–C). PB TCRβ repertoire analysis of CD161^+^ and CD161^−^ Tconv from one JIA patient showed very limited overlap (Figures 8B,C). Furthermore, we observed limited overlap in TCRβ repertoire between CD161^+^ Tconv or CD161^−^ Tconv between paired PB and SF (Figure 8C). However, it should be noted that the number of sequences obtained for CD161^+^ and CD161^−^ Tconv from JIA PB was much lower compared to JIA SF. Cell populations from JIA SF had a higher clonality score compared to cell populations from JIA PB, whereas Shannon entropy scores were similar (Figure 8D). The overlap in TCRβ repertoire between CD161^+^ and CD161^−^ Tconv and Treg from the site of inflammation contradicts the limited overlap observed in blood from controls and suggests that in an inflamed environment CD161 expression might be more labile.

## Discussion

In this study, we identified a shared transcriptional signature between CD161^+^ Tconv and CD161^+^ Treg from blood despite limited overlap in TCRβ repertoire. In addition, we reported that CD161^+^ T cells showed higher expression of tissue-homing receptors, including the gut-homing markers CCR9 and integrin α4β7; whereas expression of these markers was lower on CD161^+^ Tconv and CD161^+^ Treg from the site of inflammation in autoimmune arthritis. Furthermore, we demonstrated increased overlap in TCRβ repertoire between CD161^+^ and CD161^−^ Tconv, and CD161^+^ and CD161^−^ Treg from the inflamed site indicating that CD161 expression might be labile in inflammatory conditions.

Both CD161^+^ Tconv and CD161^+^ Treg produced cytokines and expressed higher levels of transcription factors and chemokine receptors associated with Th1 and Th17 cells compared to CD161^−^ cells. Furthermore, only CD161^+^ Tconv and CD161^+^ Treg produced IFNγ in response to IL-12 and IL-18. This might have implications for JIA as higher levels of IL-12 (only modest increase) and IL-18 were reported in SF from JIA patients with active disease compared to patients in remission (62). In addition, CD161^+^ Th17 cells can convert to Th1 cells and thereby remain CD161^+^ (29, 30) explaining both IL-17 and IFNγ production by CD161^+^ T cells and suggesting that CD161 expression may mark T cell plasticity.

The shared transcriptional signature of CD161^+^ Tconv and CD161^+^ Treg is in accordance with recent evidence demonstrating that expression of CD161 defines a common transcriptional signature between CD4^+^ and CD8^+^ T cells, γδ^+^ T cells and MAIT cells independent of classical T cell lineages (40). These data together with the transcriptional signatures reported here suggested that CD161^+^ lymphocytes might originate from a common early precursor cell. However, TCRβ repertoire analysis of CD161^+^ and CD161^−^ Tconv and Treg from blood of healthy controls demonstrated limited overlap in TCRβ repertoire between CD161^+^ Tconv and CD161^+^ Treg indicating that commitment to CD161 expression is either a very early thymic event or can occur in several cell lineages during thymocyte development.

Pathway analysis revealed that CD161^+^ T cells have a migratory phenotype and CD161^+^ Tconv and CD161^+^ Treg expressed chemokine receptors and integrins associated with tissue homing. In concurrence with this finding, studies have previously reported that CD161 expression by CD4^+^ and γδ^+^ T cells is involved in trans-endothelial migration. Resting CD4^+^CD161^+^ T cells, but not CD4^+^CD161^−^ T cells, were able to adhere and migrate through a monolayer of vascular endothelial cells using a Transwell chamber system. The trans-endothelial migration of CD4^+^CD161^+^ T cells was reduced when cells were pre-treated with anti-CD161 mAb (63–65). In addition, CD161^+^ CD4^+^ T cells have previously been detected in gut (26, 66, 67), skin (26), lung (68), and synovial tissue (69). These data indicate that CD161^+^ T cells might be more prone to migrate into tissues compared to CD161^−^ T cells. Our demonstration of high expression of tissue-homing receptors supports this concept.

Transcriptome and protein data showed higher expression of CCR9 and integrin α4β7, markers associated with gut homing (43, 44), on CD161^+^ T cells from blood. In contrast, CD161^+^ T cells from the inflamed site contained fewer CCR9^+^ and integrin α4^+^β7^+^ cells compared to blood and exposure to RA failed to alter expression of CCR9, whereas there was a trend toward increased integrin α4β7 levels within CD161^+^ T cells. RA synthesis occurs in the gut under homeostatic conditions, but can also be induced at systemic sites in response to inflammation (58–61). Upregulation of RA synthesis at peripheral sites has been shown to induce expression of CCR9 and α4β7 on lymphocytes with subsequent gut homing (59, 61). The increased expression of CCR9 and α4β7 on CD161^+^ T cells from blood as well as the ability of these cells to respond to RA to a greater degree than CD161^−^ T cells suggests that these cells may have received a RA signal during T cell priming, either in the gut or the periphery, which resulted in gut homing. The enrichment of CD161^+^ T cells in SF suggests that these cells might then migrate towards the synovial joint where they contribute to disease pathogenesis. We believe that these findings highlight a potential gut–joint axis. Future studies to demonstrate whether CD161^+^ T cells are trafficking from the gut to the joint, for example by analyzing overlap in TCRβ repertoire from CD161^+^ T cells from gut tissue and SF of the same patient, would be of interest.

In order to retain CD161^+^ T cells in the joint, our data suggest that cells lose their gut-homing phenotype and their ability to respond to RA. Possible mechanisms include downregulation caused by signals present in SF or following interaction with ligands *in vivo*. Alternatively, a high antigen dose has been reported to limit ATRA-induced CCR9 expression on activated CD8^+^ cells (70). It is very likely that cells are exposed to high antigen levels at the inflamed site and this could possibly overcome the RA-induced expression of gut-homing markers. In addition, high expression of integrin β1 was found to prevent binding of integrin α4 and β7 (47). High levels of integrin α4β1 have been observed on SF T cells in rheumatoid arthritis (71), which could explain the low expression of integrin α4β7 in JIA SF. Interestingly, CD161^+^ and CD161^−^ Tconv and Treg are highly enriched for integrin α4^+^β1^+^ cells in the synovial joint (C.L.D. unpublished data).

The maintained cytokine-producing phenotype of both CD161^+^ Tconv and CD161^+^ Treg in JIA SF is in accordance with previously published studies reporting that CD161^+^ T cells from JIA (29, 30, 72) and rheumatoid arthritis (69) patients produce multiple pro-inflammatory cytokines. In addition, CD161^+^ Treg from JIA SF showed equal suppressive capacity to CD161^−^ Treg indicating that these cells are functionally suppressive. Despite the limited overlap in TCRβ repertoire of CD161^+^ and CD161^−^ Tconv and Treg from healthy individuals, we observed considerable overlap between CD161^+^ and CD161^−^ cell populations from the inflamed site. In addition, CD161^+^ Tconv and CD161^+^ Treg showed overlap (~20%) in TCRβ repertoire suggesting that a small proportion of CD161^+^ Treg might convert to CD161^+^ Tconv or the other way around within the inflammatory environment. In a previous study, we observed limited overlap (<10%) between TCR clones of Tconv and Treg from JIA SF (33). However, in this study, Tconv and Treg were not divided into CD161^+^ and CD161^−^ cells, potentially explaining the different results. The shared TCRβ repertoire between CD161^+^ and CD161^−^ Tconv, and CD161^+^ and CD161^−^ Treg suggests that the inflamed site might create an environment that alters CD161 expression causing a switch between non- and pro-inflammatory phenotypes and thereby contributes to disease pathogenesis.

Potential limitations of this study are that TCRβ repertoire was only analyzed in a limited number of samples, which was partially due to the limited availability of paired PB and SF samples from JIA patients with sufficient cell numbers for cell sorting of all four populations. Therefore, the number of sequences obtained for CD161^+^ and CD161^−^ Tconv from JIA PB was much lower compared to JIA SF, and the TCRβ repertoire of CD161^+^ and CD161^−^ Treg from JIA PB was not analyzed due the small volumes of blood obtained from JIA patients and thus insufficient number of cells for sorting.

Data reported here suggests that trafficking of CD161^+^ T cells through the gut might be a necessary process in order to shape the course of immune responses in for example the synovial joint. The functional relevance of this process remains elusive, but the gut–joint axis could provide a novel therapeutic target as for example maybe a simple change in diet might help restore the balance in the gut and impact upon or even resolve symptoms. Analysis of TCRβ repertoire suggested that CD161 expression is regulated differently in health and disease. Therefore, it will be important to define mechanisms controlling CD161 expression. In addition, the indication that CD161 expression might be labile at the inflamed site suggests that the pro-inflammatory phenotype may be switched “on” or “off,” possibly due to factors present the synovial environment. Future studies should focus on functional relevance of CD161 and the labile CD161 expression in inflammatory conditions as this could yield great therapeutic potential.

## Author Contributions

CD, CB, RO, and LW were responsible for experimental design, interpretation of the data, and revising the manuscript for intellectual content. CD acquired and analyzed the data and drafted the manuscript. All authors approved the final version of the submitted manuscript.

## Conflict of Interest Statement

The authors declare that the research was conducted in the absence of any commercial or financial relationships that could be construed as a potential conflict of interest.

## Footnotes

^1^https://software.broadinstitute.org/GENE-E/index.html.

^2^www.qiagen.com/ingenuity.
