# Supplementary material for: CD161+ Tconv and CD161+ Treg Share a Transcriptional and Functional Phenotype despite Limited Overlap in TCRβ Repertoire
Source: Front Immunol. 2017 Mar 6;8:103. doi: 10.3389/fimmu.2017.00103 (PMC5337494; doi:10.3389/fimmu.2017.00103)
Supplement: Supplementary file 1 [file Table_1.PDF]

Supplementary Tables 1-5

CD161+ Tconv and CD161+ Treg a Share Transcriptional and Functional Phenotype despite Limited Overlap in TCRβ Repertoire

Chantal L Duurland*, Chrysothemis C Brown, Ryan FL O'Shaughnessy, Lucy R Wedderburn

***Correspondence:** Chantal L Duurland: c.duurland@ucl.ac.uk

1. Supplementary Tables

Table S1: List of antibodies used for flow cytometry and cell sorting

**Marker**	**Fluorochrome**	**Clone**	**Supplier**	**Catalog Number**
CD3	FITC	UCHT1	BD Biosciences	555916
CD3	PE	UCHT1	Biolegend	300408
CD3	PE-Cy7	UCHT1	Biolegend	300420
CD3	PerCPCy5.5	OKT3	eBioscience	45-0037-42
CD3	BV421	UCHT1	Biolegend	300434
CD3	V500	UCHT1	BD Biosciences	561416
CD3	BV605	OKT3	Biolegend	317322
CD3	BV711	OKT3	Biolegend	317328
CD3	APC	UCHT1	Biolegend	300412
CD4	BV711	OKT4	Biolegend	317440
CD25	PE	M-A251	BD Biosciences	555432
CD25	BV421	M-A251	Biolegend	356114
CD45RA	PerCPCy5.5	HI100	Biolegend	304122
CD45RO	PE-Cy7	UCHL1	Biolegend	304230
CD49d (=Integrin α4)	PerCPCy5.5	9F10	Biolegend	304312
CD49d (=Integrin α4)	BV510	9F10	Biolegend	304318
CD127	FITC	eBioRDR5	eBioscience	11-1278-42
CD161	BV605	HP-3G10	Biolegend	339916
CD161	PE	HP-3G10	eBioscience	12-1619-42
CD161	APC	HP-3G10	eBioscience	17-1619-42
CD199 (=CCR9)	PE-Cy7	L053E8	Biolegend	358910
CD195 (=CCR5)	PerCPCy5.5	J418F1	Biolegend	359112
TIGIT	PE	MBSA43	eBiosciences	12-9500-42
Integrin β7	PE	FIB504	BD Pharmingen	555945
Integrin β7	PerCPCy5.5	FIB504	Biolegend	321220
CD218a (=IL18Rα)	PE	H44	eBiosciences	12-7183-41
GITR	PE	110416	R&D	FAB689P
CTLA4	PE	14D3	eBioscience	12-1529-42
PD1	PE	eBioJ105	eBioscience	12-2799-41
CXCR3	PE	1C6/CXCR3	BD Pharmingen	557185
Ki67	eFluor450	20Raj1	eBioscience	48-5699-42
FoxP3	APC	236A/E7	eBioscience	17-4777-42

Foxp3	eFluor450	236A/E7	eBioscience	48-4777-42
Helios	Pacific Blue	22F6	Biolegend	137220
IFNγ	V500	B27	BD Biosciences	561980
IL-17A	BV605	BL168	Biolegend	512326
DAPI	n/a	n/a	Sigma-Aldrich	D9542
Propidium Iodide (PI)	n/a	n/a	Sigma-Aldrich	P4170
Live/Dead fixable blue	n/a	n/a	Thermo Fisher Scientific	L34962
Live/Dead fixable yellow	n/a	n/a	Thermo Fisher Scientific	L34967
FxCycle Violet	n/a	n/a	Thermo Fisher Scientific	F10347

Table S2: List of differentially expressed genes (1626) comparing CD161$^+$ Tconv to CD161$^-$ Tconv with *P*<0.05 and fold change ≥ 1.5

Gene symbol	*P*-value	Fold change	Up/Down
KLRB1	3.39E-08	258.1587	Up
MIR4440	1.30E-02	87.6819	Up
LTK	6.40E-06	30.9877	Up
IL18RAP	2.24E-04	25.2634	Up
COL5A3	3.40E-04	24.7934	Up
NOG	3.19E-08	17.9858	Down
NKG7	1.70E-02	17.7619	Up
CCR5	2.89E-04	16.8174	Up
GNLY	2.31E-02	16.2624	Up
HOPX	9.34E-04	16.1555	Up
CCR2	1.59E-04	15.8351	Up
CST7	1.13E-02	14.9894	Up
RORC	5.95E-05	14.1147	Up
AIF1	8.69E-06	14.0958	Down
PLXND1	7.47E-05	13.8612	Up
MYO1F	1.33E-03	13.7915	Up
LGALS3	1.59E-03	13.6729	Up
IL18R1	1.50E-04	13.5570	Up
GZMA	1.03E-02	13.4680	Up
CCR6	3.19E-05	12.6807	Up
LRRN3	3.08E-04	12.4135	Down
MAF	1.29E-03	12.3964	Up
GZMK	1.02E-02	12.2069	Up
PLEK	4.38E-03	12.0897	Up
PLEKHG3	4.71E-04	12.0527	Up
ADAM19	1.83E-04	11.8034	Up
MYBL1	1.69E-04	11.6407	Up
FOSL2	2.25E-02	11.5066	Up
TBX21	3.98E-03	11.3158	Up
PRF1	3.80E-03	11.3069	Up
CXCR6	2.74E-04	10.9396	Up
CEBPD	2.96E-04	10.8791	Up
CCL5	6.07E-03	10.6905	Up
COL18A1	4.34E-05	10.5338	Down
CD58	2.82E-04	10.4881	Up
BHLHE40	4.46E-03	10.1269	Up
DUSP5	1.89E-03	9.9771	Up
NCF4	8.19E-04	9.8613	Up
ADTRP	3.27E-04	9.7258	Down
SLC4A10	1.43E-04	9.6466	Up
PRDM1	5.48E-04	9.6227	Up
NPDC1	1.81E-04	9.5981	Up
MIR3180-5	1.39E-03	9.3575	Down
CTSH	5.30E-04	9.1018	Up
S1PR5	7.14E-04	9.0322	Up

EDAR	1.67E-04	8.9245	Down
KLRG1	5.40E-04	8.8995	Up
DACT1	5.70E-05	8.8963	Down
KRT72	3.76E-02	8.7507	Down
PTGDS	6.36E-04	8.7355	Up
AUTS2	2.93E-05	8.5751	Up
SLAMF7	2.34E-03	8.4360	Up
EOMES	3.15E-03	8.2357	Up
ANXA2	2.74E-03	8.2330	Up
FAM129A	7.79E-04	8.1993	Up
PRR5L	1.84E-03	8.0860	Up
AK5	2.86E-05	7.9656	Down
ADRB2	2.49E-04	7.7899	Up
COLQ	2.61E-04	7.7166	Up
SCML1	3.14E-03	7.6015	Down
FAM46C	4.29E-03	7.4959	Up
C10orf128	6.83E-07	7.4755	Up
F2R	9.20E-04	7.3379	Up
STOM	1.10E-03	7.2460	Up
B4GALT5	8.25E-04	7.0937	Up
TNFRSF4	1.16E-03	7.0633	Up
MAP3K8	4.00E-03	7.0358	Up
CFH	4.01E-04	7.0091	Up
MIR1260B	1.16E-04	6.8610	Down
MMP28	1.30E-06	6.8360	Down
APOBEC3H	1.26E-03	6.6674	Up
PDCD1	2.10E-03	6.5920	Up
TNFRSF18	2.95E-04	6.5114	Up
MCOLN2	2.14E-04	6.3892	Up
CD300A	3.32E-03	6.3742	Up
CYB561	2.60E-04	6.1804	Up
IGF1R	7.06E-04	6.1431	Down
ITGAM	1.39E-03	6.1312	Up
NET1	2.26E-05	6.1003	Down
ERN1	2.94E-04	6.0455	Up
LAG3	1.03E-03	5.9801	Up
ACTN1	2.25E-04	5.9770	Down
FRMD4B	2.48E-04	5.9653	Up
CXCR3	7.04E-03	5.9290	Up
MAN1C1	2.30E-05	5.9021	Down
PPP2R2B	7.39E-06	5.8227	Up
TGFBR3	2.58E-04	5.7933	Up
TSPAN15	1.92E-05	5.7372	Up
CYTH3	1.04E-04	5.7369	Up
SRXN1	3.82E-05	5.7225	Up
CSGALNACT1	2.61E-05	5.7094	Down
EPHA1	4.03E-04	5.6538	Down
PDE9A	2.33E-03	5.6385	Down

PCSK5	2.35E-04	5.6281	Down
NCALD	2.34E-05	5.5905	Up
ANKRD55	1.44E-03	5.5152	Down
PLXDC1	7.02E-04	5.4898	Down
IRF5	9.57E-06	5.4379	Up
SLC22A17	1.39E-04	5.4333	Down
SLC40A1	5.08E-04	5.3938	Down
NSG1	2.98E-02	5.3659	Up
SYT11	4.00E-03	5.3590	Up
ITPRIPL1	5.23E-04	5.3450	Up
GPR15	1.83E-03	5.3308	Up
TRGC1	7.96E-04	5.3221	Up
NCR3	1.21E-04	5.3164	Up
JAKMIP1	1.04E-02	5.2896	Up
PTPRM	5.43E-04	5.2870	Up
YWHAH	9.35E-04	5.2513	Up
TNF	3.75E-03	5.2414	Up
SERPINE2	8.50E-04	5.2321	Down
CDCA7L	1.73E-03	5.1766	Down
PREX1	1.23E-03	5.1634	Up
DFNB31	4.38E-04	5.1016	Up
CALHM2	8.56E-04	5.1014	Up
GPR114	1.47E-03	5.0145	Up
TBKBP1	8.40E-04	5.0030	Up
S100A11	5.13E-04	4.9953	Up
PLAG1	3.35E-05	4.9888	Down
BCAS4	1.66E-04	4.9004	Down
S100A4	1.35E-03	4.8839	Up
IL2RB	3.09E-04	4.8319	Up
IL12RB2	9.51E-04	4.8299	Up
CCR7	3.14E-04	4.8258	Down
C3AR1	8.23E-05	4.8081	Up
AGPAT4	1.01E-04	4.8058	Up
PHACTR2	5.27E-04	4.7951	Up
RHOC	6.88E-05	4.7744	Up
GAB3	4.17E-04	4.7735	Up
IL6ST	1.05E-05	4.7582	Down
FAS	2.19E-05	4.7511	Up
GPR68	1.05E-04	4.7228	Up
GNA15	3.87E-03	4.6955	Up
CTLA4	4.10E-03	4.6803	Up
CCR4	4.96E-03	4.6323	Up
GDPD5	2.80E-05	4.6284	Up
PYHIN1	1.28E-03	4.6194	Up
SOX13	3.23E-05	4.6075	Up
PTPN13	1.41E-04	4.5757	Up
CTNNA1	1.87E-04	4.5692	Up
PMAIP1	1.26E-02	4.5308	Up

CLCF1	8.50E-04	4.4680	Up
TMIGD2	1.42E-05	4.4659	Down
ANXA4	1.76E-03	4.4624	Up
ITGB1	4.56E-03	4.4089	Up
ACTN4	1.82E-04	4.4050	Up
DENND3	1.26E-04	4.4027	Up
IFNGR2	1.38E-04	4.3967	Down
ACE	5.14E-04	4.3966	Up
ABCA2	1.88E-04	4.3965	Up
TNFRSF10D	8.95E-04	4.3952	Down
PTGER2	3.13E-03	4.3948	Up
SH2D2A	5.81E-04	4.3883	Up
SAP30	1.11E-04	4.3782	Up
FAM153A	9.98E-04	4.3772	Down
TIGIT	1.16E-02	4.3597	Up
TBXAS1	4.44E-04	4.3478	Up
MLLT4	4.32E-05	4.3376	Up
HLA-DQB1	5.03E-03	4.3319	Up
MAN1A1	2.75E-04	4.3164	Up
EFHD2	1.13E-03	4.3009	Up
PTMS	5.59E-03	4.2604	Up
ATXN1	8.91E-04	4.2593	Up
AHNAK	1.23E-03	4.2322	Up
STAP1	5.61E-03	4.2263	Down
IGFBP3	1.45E-03	4.2174	Up
KBTBD11	3.06E-05	4.2129	Down
CARD17	3.83E-04	4.1880	Up
TP53INP1	8.08E-04	4.1805	Up
NHSL2	2.29E-04	4.1765	Up
PTK2	1.28E-05	4.1547	Down
BAIAP3	3.00E-05	4.1459	Up
KLRF1	4.47E-04	4.1372	Up
TNFRSF1B	9.37E-04	4.1017	Up
GPC2	4.03E-07	4.0994	Down
DCHS1	8.60E-05	4.0864	Down
CACHD1	4.10E-04	4.0785	Down
FAM213A	1.99E-07	4.0759	Down
OASL	2.42E-03	4.0615	Up
CR2	1.03E-04	4.0513	Down
APBA2	7.79E-06	4.0343	Down
PAM	5.00E-04	4.0260	Up
GPR160	2.15E-04	4.0191	Down
SLC22A23	1.87E-04	4.0069	Down
RHOU	1.37E-03	4.0065	Up
LEF1	3.90E-05	4.0042	Down
MAP3K5	2.45E-05	3.9764	Up
KLF10	1.88E-02	3.9732	Up
LGALS1	1.14E-02	3.9563	Up

TIPARP	2.14E-02	3.9462	Up
TRABD2A	5.11E-05	3.9429	Down
GTDC1	3.03E-04	3.9390	Up
OGFRL1	5.97E-04	3.9216	Up
ABCB1	2.57E-03	3.9215	Up
CITED4	4.47E-05	3.8932	Down
OSM	1.28E-03	3.8924	Up
IFNG	1.26E-02	3.8911	Up
C12orf75	2.01E-02	3.8831	Up
USP46	3.08E-05	3.8723	Up
CDC42EP3	9.22E-04	3.8598	Up
MXRA7	1.37E-03	3.8513	Up
SLC1A5	1.28E-04	3.8404	Up
SMCO4	9.53E-04	3.8303	Up
CAPN12	2.03E-06	3.8273	Up
DUSP2	8.88E-03	3.8226	Up
GAL3ST4	2.32E-05	3.8073	Down
CDCA7	2.04E-02	3.8070	Up
EPHX2	2.16E-04	3.8050	Down
CSF1	2.38E-04	3.7963	Up
MB21D1	1.18E-05	3.7932	Up
ATP2B4	3.59E-04	3.7700	Up
SPON2	2.10E-02	3.7588	Up
MXRA8	2.45E-05	3.7473	Down
PROSAPIP1	7.34E-05	3.7343	Down
IL15	1.01E-05	3.7234	Up
PLA2G16	1.43E-03	3.7233	Up
PDK1	8.14E-05	3.7232	Down
SULT1B1	2.75E-04	3.7152	Down
RHBDF2	2.21E-04	3.7049	Up
GSE1	4.97E-04	3.6979	Up
AGAP1	6.57E-04	3.6944	Up
DENND5A	1.85E-04	3.6905	Down
LIMS1	2.91E-04	3.6828	Up
TNFSF14	2.99E-05	3.6744	Up
NPC1	2.08E-04	3.6734	Up
NPAS2	2.73E-04	3.6489	Down
HNRPLL	1.00E-04	3.6292	Up
EMR1	2.49E-04	3.6195	Down
PRKAR1B	1.19E-05	3.5915	Down
LMO7	1.50E-04	3.5869	Down
LYAR	1.72E-04	3.5840	Up
PTGDR	4.47E-04	3.5811	Up
GBP5	2.37E-03	3.5715	Up
PLLP	5.91E-04	3.5704	Down
DDR1	1.12E-04	3.5608	Down
PITPNM2	1.12E-04	3.5525	Down
SKAP2	2.09E-03	3.5469	Up

SNPH	9.89E-06	3.5375	Down
CNKSR2	1.69E-07	3.5254	Down
PLEKHA5	1.10E-03	3.5150	Up
TTYH2	3.01E-04	3.5007	Up
TNFSF13B	2.95E-04	3.5003	Up
OBSCN	4.10E-04	3.4810	Down
KLF8	5.17E-05	3.4790	Up
SSTR3	9.96E-04	3.4745	Down
PLCL1	2.39E-04	3.4743	Down
NELL2	2.60E-03	3.4738	Down
TLE2	2.26E-03	3.4671	Down
CHI3L2	7.00E-03	3.4611	Down
SMAD3	5.28E-06	3.4505	Up
GALNT10	1.71E-04	3.4502	Up
RGMB	7.27E-03	3.4500	Down
ZC3H12A	2.57E-03	3.4055	Up
GPRIN3	2.49E-05	3.4004	Up
CAMK2N1	3.76E-04	3.3957	Up
BACH2	2.15E-05	3.3926	Down
AOAH	9.83E-03	3.3851	Down
DLG4	8.71E-05	3.3760	Down
VNN2	4.83E-04	3.3612	Down
EPHB6	8.75E-04	3.3559	Down
SYNE2	5.07E-04	3.3492	Up
CYFIP1	9.86E-08	3.3289	Up
LATS2	3.73E-03	3.3259	Up
SLAMF1	1.56E-03	3.3100	Up
RGS3	1.31E-03	3.3031	Up
ICAM1	2.79E-03	3.2954	Up
CREB3L2	3.60E-05	3.2733	Up
LGMN	1.41E-03	3.2731	Down
TNNT3	1.45E-03	3.2717	Down
H1F0	9.70E-03	3.2668	Down
GPR65	1.19E-03	3.2499	Up
SYTL2	5.20E-05	3.2446	Up
FGFR1	4.09E-03	3.2436	Up
SLC16A3	1.15E-03	3.2277	Up
RCN3	2.73E-05	3.2277	Down
ARAP2	8.40E-04	3.2039	Up
GFI1	3.56E-03	3.2033	Up
APOBEC3C	7.50E-03	3.2020	Up
FCGRT	1.37E-04	3.1952	Down
PDZD4	1.35E-02	3.1918	Up
MBOAT1	5.40E-05	3.1908	Up
NOD2	1.32E-03	3.1821	Up
THEM5	1.18E-04	3.1766	Up
KANK1	2.34E-02	3.1727	Down
MICAL2	8.73E-04	3.1727	Up

PARVB	4.32E-02	3.1723	Up
ARMCX2	7.99E-07	3.1539	Down
ZNF844	7.06E-03	3.1538	Down
CKS2	2.94E-03	3.1508	Up
SETD7	9.49E-04	3.1427	Up
RAB27A	1.08E-03	3.1361	Up
RAP1GAP2	1.25E-03	3.1331	Up
RBM11	9.48E-03	3.1196	Down
PTTG1	1.01E-03	3.1157	Up
HKDC1	4.52E-03	3.1073	Down
APOBR	1.52E-02	3.1061	Up
OCRL	6.13E-05	3.1017	Down
SRGN	2.39E-02	3.0929	Up
ARHGEF12	4.08E-04	3.0908	Up
ALS2CL	2.06E-03	3.0787	Down
TCEA3	2.23E-03	3.0782	Down
NBEAL2	7.57E-05	3.0739	Up
DLG3	2.74E-04	3.0671	Up
MATK	1.08E-03	3.0482	Up
TMIE	5.94E-05	3.0464	Down
CRIP1	1.98E-04	3.0435	Up
LIMS3L	6.02E-09	3.0380	Up
LIMS3	6.02E-09	3.0380	Up
CCDC50	2.30E-02	3.0352	Up
PDE4D	2.95E-02	3.0306	Up
ZNF516	4.61E-04	3.0241	Down
CLIC1	3.56E-03	3.0235	Up
CXCR5	2.86E-02	3.0225	Up
RORA	2.50E-05	3.0182	Up
CTSL1	4.40E-02	3.0177	Down
TSPAN5	4.08E-03	3.0167	Up
SYNM	9.31E-03	3.0033	Up
ME3	1.30E-02	3.0005	Down
NUAK2	2.31E-03	2.9838	Down
MAL	6.56E-04	2.9801	Down
IFNGR1	1.46E-03	2.9763	Up
TBK1	1.20E-03	2.9741	Up
CFP	3.09E-03	2.9725	Down
SPINT2	7.43E-04	2.9720	Down
NTN4	4.84E-04	2.9717	Up
BCL6	2.16E-04	2.9712	Up
PDE4A	2.76E-05	2.9638	Up
CYSLTR1	1.19E-03	2.9627	Down
VCL	4.94E-04	2.9602	Up
IL32	4.65E-02	2.9588	Up
ELOVL6	2.63E-05	2.9395	Up
CYSTM1	1.12E-04	2.9354	Up
APOBEC3G	2.85E-03	2.9293	Up

ASIC3	2.89E-03	2.9244	Down
RHEBL1	7.32E-05	2.9092	Up
RALGPS2	4.72E-04	2.9072	Down
TMEM156	1.04E-03	2.9063	Up
SPEG	6.86E-03	2.8906	Down
KCNQ1	1.28E-04	2.8878	Down
GP5	5.10E-04	2.8834	Down
GYG1	6.14E-04	2.8801	Up
RIN3	3.03E-04	2.8791	Down
PIEZO1	3.36E-05	2.8790	Up
SSBP4	4.54E-04	2.8787	Up
FURIN	2.25E-02	2.8762	Up
DSC1	4.54E-04	2.8634	Down
SARDH	1.65E-02	2.8604	Down
RAB11FIP1	1.97E-04	2.8570	Up
PLXNC1	1.26E-03	2.8553	Up
UEVLD	9.47E-03	2.8530	Up
SLC35F2	9.42E-04	2.8529	Up
COL6A1	6.84E-03	2.8452	Down
SUSD4	2.42E-02	2.8419	Down
TMEM220	6.25E-04	2.8402	Down
SNN	4.36E-03	2.8324	Down
FKBP11	1.33E-04	2.8112	Up
ADAM15	2.95E-04	2.8061	Up
RNF175	2.37E-03	2.8034	Down
CD151	1.96E-02	2.8033	Up
C1orf216	4.13E-03	2.8031	Up
DUSP10	6.82E-03	2.7952	Up
NGFRAP1	4.36E-02	2.7949	Down
CAMSAP2	1.03E-03	2.7917	Down
CHMP7	2.42E-04	2.7913	Down
BEND5	8.19E-04	2.7866	Down
CARNS1	2.53E-03	2.7844	Down
HN1	3.54E-04	2.7841	Up
IL10RA	1.72E-04	2.7825	Up
TESC	2.66E-02	2.7713	Up
CRB3	5.92E-06	2.7702	Down
DNAJC1	8.03E-04	2.7693	Up
TIFA	2.33E-03	2.7687	Up
ITGA6	9.89E-06	2.7686	Down
FAM134B	1.82E-03	2.7677	Down
REEP5	2.85E-04	2.7652	Up
TNK1	1.57E-04	2.7540	Down
TXK	2.27E-04	2.7535	Down
F5	1.51E-04	2.7510	Up
WEE1	1.30E-05	2.7501	Up
AGPHD1	2.96E-04	2.7456	Down
CD74	1.96E-03	2.7398	Up

KLF7	5.98E-04	2.7388	Down
ADAP1	2.52E-03	2.7382	Up
CTSC	6.97E-03	2.7354	Up
ALOX5AP	5.40E-04	2.7335	Up
CD84	3.85E-02	2.7312	Up
PHGDH	1.78E-03	2.7206	Down
KLF6	2.71E-03	2.7197	Up
MUC1	6.69E-04	2.7186	Up
GALM	2.05E-03	2.7122	Up
GRAPL	5.54E-04	2.7087	Down
APBB1	1.37E-05	2.7060	Down
ATHL1	2.66E-02	2.7046	Down
CLDND1	6.14E-03	2.7020	Up
MPST	5.57E-03	2.7003	Up
LRP8	8.86E-05	2.7002	Up
NEK6	2.09E-03	2.6967	Up
BZRAP1	2.07E-04	2.6966	Up
RNF207	5.54E-05	2.6925	Up
ZC3H12D	3.13E-03	2.6897	Up
IL4R	3.35E-04	2.6856	Down
SIPA1L2	2.07E-03	2.6833	Up
MICAL3	7.75E-04	2.6773	Up
CACNA2D2	1.02E-02	2.6758	Up
B4GALT4	1.67E-04	2.6736	Down
MAML2	1.43E-04	2.6704	Down
SRGAP2	1.71E-05	2.6703	Up
RASSF1	9.92E-05	2.6652	Up
IFI30	2.61E-03	2.6619	Up
SH3BGRL3	1.08E-04	2.6592	Up
ACOT9	7.58E-05	2.6561	Up
FLNB	2.08E-05	2.6501	Down
SERTAD3	6.37E-03	2.6496	Up
AGO4	9.37E-05	2.6491	Up
CHST12	4.56E-03	2.6453	Up
GSTM2	5.73E-03	2.6441	Down
LRRN2	1.46E-04	2.6398	Down
EPHB4	3.04E-05	2.6384	Down
BCL7A	2.23E-06	2.6378	Down
CHML	9.30E-06	2.6374	Down
FUT7	3.13E-03	2.6325	Up
YWHAQ	3.67E-04	2.6322	Up
GALNT3	1.33E-03	2.6313	Up
DPY19L1	7.26E-04	2.6274	Up
KCTD15	1.20E-04	2.6230	Up
ARHGAP10	2.03E-03	2.6230	Up
FAM63A	3.75E-03	2.6180	Down
SPATC1L	3.77E-03	2.6170	Down
PHTF1	9.97E-05	2.6143	Up

NOXA1	1.45E-03	2.6098	Down
PLEKHB1	1.40E-04	2.6092	Down
GCA	2.41E-04	2.6064	Down
BCL2L1	2.53E-03	2.6057	Up
ANXA1	4.13E-03	2.6055	Up
RAB34	1.84E-02	2.6043	Down
MT1E	8.69E-03	2.5991	Up
FADS3	1.22E-02	2.5963	Up
TMEM64	7.62E-05	2.5962	Up
ACSS2	5.08E-05	2.5929	Down
IL17RE	4.42E-03	2.5915	Up
EVA1B	2.11E-03	2.5898	Up
CELA1	1.34E-02	2.5892	Down
UST	3.33E-04	2.5866	Up
OXNAD1	2.04E-06	2.5835	Down
CENPM	3.21E-03	2.5807	Up
GALC	3.05E-04	2.5799	Up
SOX12	1.99E-04	2.5710	Down
MTUS1	1.16E-02	2.5702	Down
S100A10	9.59E-04	2.5686	Up
ADAM8	1.23E-04	2.5677	Up
DBH	2.77E-02	2.5627	Down
AHR	4.58E-04	2.5612	Up
ZBTB18	2.65E-03	2.5571	Down
KCTD3	2.85E-04	2.5557	Down
LTBP3	2.86E-05	2.5524	Down
ITGA4	7.63E-04	2.5474	Up
NLGN2	1.57E-04	2.5466	Down
SMPD1	1.32E-03	2.5452	Down
SLC16A5	6.77E-03	2.5438	Down
NABP1	1.27E-03	2.5437	Up
MPP1	1.05E-02	2.5412	Down
ALCAM	9.77E-04	2.5405	Up
USP6NL	4.97E-05	2.5345	Down
NREP	2.05E-05	2.5337	Down
B4GALT1	1.56E-04	2.5335	Up
Sep-08	2.12E-04	2.5327	Up
TOP1MT	3.35E-05	2.5226	Down
HHAT	3.14E-06	2.5197	Up
CDC42BPG	3.78E-04	2.5187	Down
C1orf162	4.30E-03	2.5182	Down
BTG3	1.07E-02	2.5137	Up
ARHGAP18	8.16E-05	2.5124	Up
IKZF3	1.05E-03	2.5099	Up
MCF2L	4.67E-05	2.5058	Down
ADPRM	3.25E-05	2.5036	Down
TP53I11	3.92E-02	2.5036	Up
DUSP16	3.46E-04	2.5028	Up

LPGAT1	6.84E-04	2.4994	Up
SELL	5.73E-04	2.4963	Down
LRRC16A	1.64E-04	2.4911	Down
TIMP1	4.70E-04	2.4895	Up
MPZL3	2.12E-03	2.4866	Up
CACNB3	1.61E-03	2.4855	Up
IL15RA	9.32E-04	2.4813	Up
RNF19B	1.17E-04	2.4804	Up
PLCB1	3.45E-06	2.4784	Up
PRNP	1.34E-05	2.4776	Up
TPCN1	1.89E-05	2.4727	Down
ID3	1.55E-02	2.4697	Down
CCDC107	4.99E-03	2.4688	Up
USP28	7.11E-03	2.4680	Up
TAF13	1.87E-02	2.4668	Up
SLC24A6	3.77E-05	2.4660	Down
TXN	2.07E-04	2.4657	Up
AXIN2	2.70E-03	2.4655	Down
HID1	4.08E-05	2.4651	Down
CHST11	4.83E-04	2.4641	Up
FAM101B	2.79E-02	2.4627	Down
TMX4	1.89E-04	2.4605	Up
RASGEF1A	6.07E-04	2.4588	Up
CACNA2D4	1.12E-03	2.4542	Up
SOX8	9.63E-03	2.4527	Down
NFKBIE	3.19E-02	2.4508	Up
LPCAT1	7.23E-04	2.4432	Up
PERP	1.24E-03	2.4392	Up
RNF135	3.07E-03	2.4380	Up
ANTXR2	9.03E-05	2.4335	Up
SUSD1	1.13E-03	2.4286	Up
PHLDA1	5.13E-04	2.4281	Up
IER5	1.17E-02	2.4250	Up
PRDM8	7.74E-05	2.4245	Up
SAMSN1	1.54E-02	2.4229	Up
TMCC3	2.52E-03	2.4178	Up
HIVEP1	1.24E-03	2.4144	Down
RNF144A	4.90E-04	2.4144	Down
KLHL3	3.67E-04	2.4138	Down
ARNTL	5.15E-05	2.4111	Up
JAKMIP2	3.33E-02	2.4104	Up
PRKCA	7.57E-04	2.4047	Down
ZSCAN18	4.98E-03	2.3999	Down
ARHGEF11	8.76E-03	2.3990	Down
HLF	3.18E-04	2.3973	Up
TTYH3	6.82E-07	2.3961	Down
PRRT1	2.29E-05	2.3922	Down
ANXA5	1.06E-04	2.3919	Up

CD274	1.16E-03	2.3914	Up
CERKL	1.32E-03	2.3912	Up
PPAP2A	1.09E-04	2.3900	Down
STX11	6.87E-05	2.3882	Up
RENBP	1.75E-04	2.3866	Down
ANK1	3.11E-03	2.3859	Up
TMEM62	3.32E-03	2.3849	Up
ACVR1C	4.96E-03	2.3843	Down
PTPN22	5.59E-03	2.3837	Up
RUNX3	3.95E-03	2.3826	Up
MDK	3.07E-02	2.3801	Down
GFPT2	9.44E-04	2.3800	Up
CD7	4.13E-04	2.3780	Down
HRH2	1.20E-03	2.3728	Down
TTC38	6.36E-03	2.3692	Up
SLC16A10	1.48E-05	2.3689	Down
KRT18	1.06E-03	2.3665	Down
HOOK1	2.93E-04	2.3636	Down
ACPL2	1.61E-03	2.3627	Down
ZNF658	2.14E-02	2.3613	Down
OGDH	4.67E-04	2.3561	Up
SMAD5	4.41E-03	2.3509	Up
FAM117B	2.84E-03	2.3456	Down
LPHN1	1.02E-04	2.3427	Down
SESN2	2.99E-03	2.3421	Up
GPRASP1	1.31E-03	2.3373	Down
CDYL2	3.94E-04	2.3353	Up
PLEKHG4	6.53E-05	2.3326	Down
ATG9B	1.80E-03	2.3319	Down
GPR125	1.87E-03	2.3300	Down
GNA11	1.25E-03	2.3291	Down
GPR137B	2.41E-03	2.3252	Up
CERS6	6.86E-05	2.3226	Down
RRAS2	1.21E-02	2.3209	Up
IL12RB1	1.79E-04	2.3187	Up
LMO4	3.69E-03	2.3158	Up
INF2	2.73E-06	2.3108	Down
MRPL10	6.29E-03	2.3076	Up
NRM	2.41E-03	2.3048	Up
SGK223	3.12E-04	2.3033	Down
AQP3	3.30E-04	2.3032	Up
AMIGO1	3.01E-03	2.3020	Down
HEMGN	2.61E-03	2.3019	Down
PSD	6.30E-06	2.3016	Down
FAM53B	1.68E-03	2.3007	Up
USP45	3.52E-03	2.2985	Up
FUCA2	4.99E-03	2.2973	Up
ZNF439	3.25E-02	2.2961	Down

PDGFB	4.26E-02	2.2958	Up
ACSM3	1.32E-03	2.2941	Down
FLNA	2.32E-04	2.2934	Up
MYB	8.97E-03	2.2932	Down
PNPLA7	1.02E-02	2.2932	Down
SCNN1D	4.65E-04	2.2904	Down
TOX	1.78E-02	2.2895	Up
ADCY9	3.62E-03	2.2879	Up
SNED1	9.36E-03	2.2876	Down
FBP1	1.21E-03	2.2852	Down
AKR1C3	4.12E-02	2.2837	Up
GRAMD1B	9.88E-03	2.2834	Up
C1orf228	3.86E-03	2.2813	Down
RGS12	1.12E-04	2.2807	Down
NAP1L3	7.71E-04	2.2790	Down
ARHGAP35	3.20E-04	2.2774	Up
ALKBH2	1.49E-03	2.2774	Down
BCL9	1.23E-03	2.2655	Down
LUZP1	9.25E-04	2.2636	Up
BCL2L11	3.06E-03	2.2566	Up
ARRDC5	2.76E-04	2.2539	Down
EAF1	7.44E-04	2.2538	Up
EGFL8	1.50E-03	2.2522	Down
GLIPR1	1.01E-03	2.2521	Up
DLG5	7.07E-05	2.2481	Up
TPST2	1.04E-02	2.2479	Up
CNN3	1.52E-02	2.2448	Down
RGS2	1.54E-03	2.2426	Up
PPP1R16B	9.95E-03	2.2417	Up
TGFBI	1.02E-02	2.2408	Up
KIAA0895L	6.91E-04	2.2407	Up
SNX10	3.59E-03	2.2406	Up
WNT1	1.19E-03	2.2402	Up
CMC1	2.31E-02	2.2395	Up
P2RX5	3.06E-03	2.2393	Up
REEP6	4.63E-04	2.2386	Down
LRIG1	2.18E-03	2.2382	Up
SREBF1	1.69E-04	2.2379	Down
CPNE8	2.68E-04	2.2353	Up
EPHA4	7.24E-03	2.2317	Up
GNG7	3.19E-05	2.2306	Down
MAPKAPK2	1.57E-03	2.2297	Up
PCED1B	4.41E-05	2.2271	Down
GGT7	3.08E-04	2.2252	Down
FCGBP	2.17E-02	2.2244	Down
NPTXR	1.15E-02	2.2227	Down
ZNF358	3.44E-03	2.2217	Down
ABHD15	3.76E-03	2.2197	Up

CAPN2	3.99E-04	2.2188	Up
CD63	7.08E-04	2.2166	Up
SLAMF6	2.14E-02	2.2156	Up
RNF19A	3.06E-03	2.2120	Up
UBE2E2	2.85E-05	2.2117	Down
FAM216A	5.06E-03	2.2092	Down
BATF	1.15E-02	2.2034	Up
OPTN	7.22E-04	2.2028	Up
TFEB	6.09E-03	2.2011	Up
IFI44	1.71E-02	2.2001	Up
CAMKK1	3.65E-03	2.1999	Down
BMP2K	3.70E-04	2.1977	Down
HERPUD1	1.57E-02	2.1964	Up
LDLRAP1	5.96E-04	2.1928	Down
ZBP1	8.98E-04	2.1921	Up
MYO5A	2.64E-04	2.1905	Up
ZNF300	1.21E-04	2.1870	Down
PDIA6	2.65E-04	2.1862	Up
ADD2	2.89E-05	2.1850	Down
CEP170	2.08E-04	2.1839	Down
NEO1	3.48E-03	2.1834	Up
GRAP	6.53E-06	2.1711	Down
B3GNT9	3.23E-02	2.1704	Up
CHST2	1.30E-03	2.1691	Down
ZNF609	4.84E-06	2.1689	Down
CCDC167	1.57E-02	2.1672	Up
PHTF2	4.37E-04	2.1666	Up
SLC29A2	4.47E-04	2.1599	Down
MAGEF1	1.46E-03	2.1598	Down
WIPI1	1.91E-03	2.1571	Up
FAM160B1	1.38E-02	2.1506	Up
GPRC5B	6.58E-03	2.1495	Down
CD226	6.09E-03	2.1490	Up
YPEL1	1.06E-02	2.1478	Up
ZNF662	2.18E-03	2.1438	Down
KIF21A	3.09E-03	2.1413	Up
EDARADD	4.89E-03	2.1364	Up
CBLL1	1.12E-03	2.1351	Up
KCTD12	1.31E-02	2.1313	Down
PPP4R1	1.57E-03	2.1281	Up
PPT2	1.86E-03	2.1280	Down
RAPGEF2	7.62E-03	2.1248	Up
WSB2	1.14E-03	2.1237	Up
DYNLL1	7.53E-03	2.1233	Up
COMTD1	1.06E-02	2.1192	Up
FBXO10	1.80E-05	2.1141	Up
ARAP3	5.67E-03	2.1138	Up
DIAPH2	1.27E-03	2.1119	Up

FAM179A	4.34E-02	2.1101	Up
AKR1E2	1.39E-02	2.1084	Down
AKR1C1	8.80E-04	2.1067	Down
GPA33	1.21E-02	2.1065	Down
CERK	5.43E-05	2.1038	Up
LMTK3	1.34E-03	2.1032	Down
ZNF629	7.37E-05	2.1007	Down
FBLN5	3.93E-03	2.0997	Down
RDX	1.95E-03	2.0997	Up
REEP3	3.12E-03	2.0995	Up
LTBP4	2.16E-03	2.0994	Up
APP	1.49E-03	2.0962	Down
RAB15	3.16E-03	2.0931	Down
CACNB1	1.65E-03	2.0920	Up
TOB2	1.38E-04	2.0893	Up
SLC25A24	1.51E-02	2.0892	Up
ZDHHC14	1.22E-03	2.0811	Up
CBLB	4.83E-03	2.0803	Up
MVB12B	2.08E-02	2.0792	Up
ZFP28	2.82E-02	2.0791	Down
SLC25A20	9.92E-03	2.0789	Up
CLSTN3	9.67E-03	2.0777	Up
ATP2B1	1.00E-03	2.0771	Up
GLCCI1	8.77E-06	2.0771	Up
GSR	1.71E-03	2.0761	Up
AMN1	6.08E-03	2.0731	Down
ATP1B3	1.29E-03	2.0709	Up
MLLT3	7.22E-04	2.0704	Down
BBC3	4.69E-02	2.0699	Down
AAED1	2.00E-02	2.0668	Up
SAMD3	1.32E-02	2.0634	Up
RILPL2	9.86E-03	2.0626	Up
HAGHL	2.18E-03	2.0611	Up
SSBP2	7.86E-05	2.0606	Down
SLC27A3	1.64E-03	2.0594	Up
ZBTB46	1.43E-03	2.0588	Down
ARHGAP11A	4.37E-03	2.0581	Up
DHRS3	2.26E-03	2.0580	Down
SEC24D	3.00E-04	2.0545	Up
FHOD1	1.84E-03	2.0531	Up
SPTY2D1	1.79E-02	2.0507	Up
ADHFE1	6.01E-03	2.0506	Up
SESN3	2.69E-03	2.0486	Down
PTGIR	8.48E-03	2.0481	Down
DNMT3A	5.13E-06	2.0477	Down
GPR18	1.93E-03	2.0476	Down
TAF4B	3.65E-03	2.0475	Down
MPP6	3.07E-02	2.0470	Down

NUCB2	3.00E-04	2.0453	Down
DBNDD1	2.66E-02	2.0441	Down
SIGLEC9	7.47E-03	2.0438	Up
CD2	1.73E-03	2.0434	Up
TBC1D19	1.64E-03	2.0417	Up
NCOA7	3.29E-04	2.0415	Up
EVA1C	7.38E-03	2.0414	Up
RIMKLB	3.42E-03	2.0408	Down
ARHGEF39	6.22E-05	2.0407	Up
LYRM9	1.84E-03	2.0394	Down
GDE1	1.67E-03	2.0367	Up
ITGAL	5.00E-04	2.0362	Up
VIPR1	4.56E-02	2.0360	Down
CLIC5	7.05E-07	2.0359	Up
PLEKHA2	7.73E-03	2.0351	Up
SATB1	5.70E-04	2.0349	Down
CHST7	6.27E-05	2.0338	Up
MCTP2	4.02E-04	2.0335	Up
RAB3GAP1	1.08E-03	2.0332	Down
SNTB2	1.83E-02	2.0331	Up
RARG	1.83E-04	2.0319	Up
TUBB4B	4.34E-03	2.0313	Up
TSHZ2	4.74E-02	2.0306	Down
FAR2	4.93E-02	2.0304	Up
TTN	4.91E-07	2.0295	Down
EEA1	1.22E-02	2.0290	Down
ABLIM1	1.01E-04	2.0287	Down
F8	2.78E-02	2.0281	Up
KIF19	4.98E-02	2.0256	Up
RAB7L1	2.95E-03	2.0254	Up
TIAM1	2.65E-02	2.0248	Down
NSMF	1.03E-03	2.0218	Down
SAMD1	1.08E-03	2.0207	Down
SLFN11	8.34E-05	2.0200	Up
KDSR	4.12E-02	2.0158	Up
RGS10	6.32E-05	2.0155	Down
C20orf96	5.29E-03	2.0137	Down
ARHGAP32	2.64E-02	2.0120	Down
SNX9	1.40E-03	2.0110	Down
TMEM25	1.02E-03	2.0107	Down
IQGAP2	1.02E-03	2.0104	Up
PIK3IP1	3.55E-03	2.0091	Down
SESN1	2.02E-03	2.0063	Up
MYO1D	1.96E-04	2.0060	Up
TGFBR2	3.40E-05	2.0050	Down
PBX3	1.45E-03	2.0032	Down
APMAP	2.01E-03	2.0020	Up
MYO1G	8.63E-03	2.0012	Up

HVCN1	1.59E-02	2.0002	Down
EFEMP2	2.45E-03	1.9992	Down
FUOM	3.37E-02	1.9975	Down
SMS	8.23E-05	1.9966	Up
TRIM69	2.50E-02	1.9965	Up
RAPGEF1	3.58E-03	1.9950	Up
EIF4G3	1.68E-04	1.9942	Up
AHDC1	4.85E-03	1.9924	Down
KANSL1L	1.55E-03	1.9907	Down
FMNL3	7.61E-03	1.9895	Down
ADAMTSL5	4.14E-03	1.9874	Down
SGPP2	4.93E-03	1.9873	Up
EZR	3.43E-02	1.9873	Up
SAP25	3.36E-03	1.9850	Down
CENPV	9.92E-03	1.9823	Down
SLC25A23	5.40E-03	1.9805	Down
DAGLB	2.18E-02	1.9805	Down
VAV3	2.98E-03	1.9800	Up
LRP6	1.25E-04	1.9775	Down
CMTM6	1.34E-03	1.9760	Up
YARS	7.73E-03	1.9758	Up
OPN3	1.09E-02	1.9747	Down
ZBTB38	2.04E-02	1.9741	Up
KAT2B	8.53E-04	1.9740	Up
C9orf142	1.77E-03	1.9728	Up
NT5C3B	7.49E-04	1.9693	Down
FANCL	2.44E-04	1.9693	Up
PPP3CA	2.32E-04	1.9689	Up
C6orf48	2.80E-07	1.9681	Down
ATP6V1G2	1.31E-02	1.9678	Down
PCYT1A	3.77E-04	1.9661	Up
IGFBP4	1.97E-02	1.9652	Up
ALDH5A1	8.75E-03	1.9638	Down
RALGDS	8.58E-05	1.9633	Up
STOML1	1.11E-03	1.9628	Down
ADAMTS10	1.81E-02	1.9617	Down
ANXA9	2.17E-04	1.9614	Down
FAM214B	1.75E-02	1.9603	Up
TRADD	8.08E-04	1.9597	Up
NOSIP	1.77E-03	1.9544	Down
MTHFD2	6.85E-03	1.9521	Up
EEPD1	1.00E-02	1.9518	Up
GLIPR2	3.44E-02	1.9508	Up
ELOVL5	5.00E-05	1.9507	Up
KLHL6	3.94E-03	1.9505	Down
TMEM30B	1.54E-03	1.9496	Down
AGFG2	1.60E-02	1.9488	Up
SH3YL1	7.49E-03	1.9479	Down

KCNH2	1.22E-03	1.9466	Down
HIST2H2AA3	2.01E-02	1.9452	Up
HIST2H2AA4	2.01E-02	1.9452	Up
SNTA1	7.56E-03	1.9425	Up
ALKBH7	3.76E-03	1.9418	Down
TNNC1	4.67E-02	1.9407	Down
SQRDL	1.27E-03	1.9398	Up
MTA3	3.44E-03	1.9397	Down
MARCKSL1	1.47E-02	1.9397	Down
HSF2	1.38E-04	1.9395	Down
JAK2	1.30E-03	1.9394	Up
ZNF831	2.79E-03	1.9389	Up
ZNF467	3.45E-02	1.9371	Down
PTPN7	1.52E-03	1.9366	Up
CD81	2.35E-03	1.9353	Up
APOL1	3.21E-03	1.9336	Up
PNP	3.12E-03	1.9333	Up
CFL2	2.00E-03	1.9319	Up
PPARD	2.33E-04	1.9306	Down
FYCO1	2.70E-04	1.9305	Up
ZSWIM1	2.01E-03	1.9301	Down
SLC38A5	1.26E-02	1.9288	Up
AMIGO2	2.81E-03	1.9271	Down
C2orf40	8.25E-03	1.9246	Down
MEOX1	2.53E-02	1.9242	Up
REEP4	3.48E-02	1.9241	Up
ELOVL4	2.21E-02	1.9234	Up
ZNF302	4.65E-03	1.9228	Down
MAP1LC3B2	2.96E-03	1.9218	Up
PHPT1	3.20E-03	1.9214	Up
BMPR1A	4.65E-03	1.9198	Up
RAP2A	8.61E-03	1.9197	Up
TCF7	1.04E-04	1.9184	Down
CASP1	1.56E-02	1.9175	Up
GIPC3	2.34E-04	1.9169	Down
SLC35D2	7.29E-04	1.9161	Up
DLL1	2.06E-02	1.9141	Down
ZC4H2	9.82E-03	1.9119	Down
TRIP6	1.55E-03	1.9105	Down
ID2	4.21E-04	1.9088	Up
TMEM38B	5.43E-03	1.9087	Down
LYRM5	5.13E-04	1.9080	Down
MGAT2	1.35E-03	1.9071	Up
NCKAP5L	2.51E-03	1.9071	Down
ZNF414	6.71E-03	1.9068	Down
PTPRE	1.12E-03	1.9066	Up
FAM169A	9.69E-05	1.9059	Down
ZIK1	1.35E-02	1.9055	Down

CASP10	9.38E-04	1.9049	Down
CASK	3.93E-04	1.9038	Up
ST3GAL4	1.67E-03	1.9034	Up
HSBP1L1	9.77E-03	1.9022	Down
ABCB6	7.66E-03	1.9020	Down
TREML2	3.46E-03	1.9002	Down
CAPN5	4.22E-05	1.8989	Down
PTP4A3	8.45E-03	1.8987	Down
ZNF768	6.27E-04	1.8985	Down
TP53I13	9.72E-04	1.8984	Up
SYNE1	1.01E-02	1.8984	Up
TFR2	7.11E-04	1.8981	Up
NABP2	6.28E-03	1.8977	Up
SURF4	1.03E-04	1.8968	Up
CCNE1	6.26E-03	1.8955	Down
RAB30	3.87E-02	1.8949	Down
NEDD9	6.86E-03	1.8946	Up
SNAI3	2.96E-03	1.8945	Up
MYH3	4.44E-05	1.8943	Down
CNKSR1	7.05E-03	1.8935	Down
SLC6A6	4.06E-03	1.8919	Up
EIF4EBP1	2.37E-02	1.8907	Up
FUT11	4.75E-02	1.8903	Up
IKZF2	3.98E-02	1.8885	Up
TCEAL1	1.99E-03	1.8881	Down
TMEM177	1.45E-02	1.8879	Down
CDK20	5.31E-04	1.8877	Down
PTPDC1	7.75E-05	1.8872	Down
GATA3	1.76E-04	1.8861	Up
TGFB1	9.74E-03	1.8850	Up
ZNF593	4.16E-02	1.8840	Up
INPP5A	3.96E-04	1.8827	Down
PNPLA4	1.13E-02	1.8826	Down
IFI16	5.28E-03	1.8821	Up
DYRK1B	6.80E-04	1.8800	Up
DHX32	8.54E-03	1.8800	Down
EPB41L4A	5.68E-03	1.8787	Down
EPHX1	1.49E-03	1.8777	Down
NAA50	1.31E-03	1.8777	Up
LIPT2	7.31E-03	1.8766	Down
SFMBT2	4.25E-04	1.8764	Down
MIS18BP1	1.07E-02	1.8758	Up
MEST	9.16E-04	1.8716	Down
ELL	2.53E-03	1.8707	Up
DET1	3.39E-03	1.8706	Down
SPPL2A	9.20E-04	1.8702	Up
CD27	3.05E-03	1.8694	Down
NDC80	1.97E-02	1.8689	Up

HIP1R	8.18E-04	1.8665	Down
PICALM	1.18E-04	1.8651	Up
SLC25A43	1.44E-02	1.8649	Up
BEX5	2.04E-02	1.8644	Down
MACROD1	5.64E-03	1.8635	Down
NUGGC	3.49E-02	1.8631	Up
NUSAP1	1.65E-03	1.8614	Up
CHN1	1.27E-02	1.8613	Up
NAA16	2.10E-05	1.8612	Down
IL24	3.13E-03	1.8606	Down
METTL21B	2.56E-02	1.8602	Up
SH2B3	3.19E-04	1.8600	Up
GGT1	3.23E-02	1.8590	Up
MAST2	1.18E-03	1.8586	Up
ZNF853	1.05E-03	1.8571	Down
MGAT5	4.86E-03	1.8569	Up
KCNN4	1.83E-02	1.8569	Up
DGKD	1.51E-04	1.8563	Down
TRIM46	3.04E-03	1.8558	Up
ZNF532	4.34E-02	1.8554	Up
IQGAP1	7.91E-03	1.8548	Up
FZD6	8.54E-03	1.8545	Down
RABGAP1L	2.87E-03	1.8544	Up
SIAE	5.87E-03	1.8533	Down
PARP8	2.18E-03	1.8509	Up
SEC61B	4.07E-03	1.8497	Up
CIDEB	2.45E-04	1.8492	Up
GCNT4	2.60E-02	1.8492	Down
NME4	4.55E-03	1.8473	Down
NETO2	8.83E-03	1.8468	Up
C2CD2	1.11E-03	1.8460	Down
TECPR1	4.90E-04	1.8458	Down
LDOC1L	4.07E-02	1.8452	Up
SPN	1.86E-02	1.8429	Up
ATP1A3	4.23E-03	1.8422	Up
GRINA	1.08E-03	1.8422	Down
PGAM2	1.84E-02	1.8399	Down
NFKBIA	4.58E-02	1.8399	Up
GAMT	8.54E-04	1.8397	Down
C12orf57	1.29E-04	1.8375	Down
PTPRJ	7.77E-04	1.8362	Up
SLC48A1	3.60E-03	1.8360	Down
CDCA4	3.52E-03	1.8346	Up
NDRG1	1.91E-03	1.8341	Up
SLC9A9	4.89E-03	1.8340	Up
SPOPL	3.27E-04	1.8337	Up
NPM3	9.95E-04	1.8308	Down
BEX2	5.48E-03	1.8304	Down

STARD4	1.55E-02	1.8304	Up
SIAH2	1.95E-02	1.8283	Up
EFCAB13	4.79E-02	1.8271	Down
EPB41L5	1.42E-04	1.8269	Down
GSTT1	2.52E-02	1.8267	Down
FGL2	2.02E-03	1.8229	Up
ARHGAP26	5.12E-03	1.8223	Up
MAP4	4.17E-03	1.8206	Up
SNTB1	5.82E-03	1.8202	Down
C2orf81	6.55E-03	1.8199	Down
LST1	7.66E-03	1.8197	Up
CASP8	8.45E-04	1.8191	Up
ZNF69	7.64E-03	1.8189	Down
CROT	3.94E-04	1.8179	Up
SMPD3	8.11E-03	1.8150	Down
STK39	2.69E-03	1.8122	Up
DLEC1	2.39E-03	1.8116	Up
ATP6V0D1	4.53E-03	1.8112	Up
SETD1B	3.76E-03	1.8112	Down
SLC39A11	1.97E-02	1.8109	Up
KIF3B	2.15E-04	1.8107	Up
TANK	1.98E-04	1.8106	Up
EFHC2	1.06E-02	1.8101	Down
HYOU1	5.47E-04	1.8074	Up
ARPC5L	1.64E-03	1.8071	Up
MGME1	1.77E-02	1.8071	Up
ITPKB	3.11E-04	1.8068	Down
LOH12CR1	7.95E-03	1.8061	Up
IFFO2	5.20E-03	1.8058	Up
ZNF267	3.64E-04	1.8049	Up
BCL11B	1.27E-04	1.8049	Down
IDH2	3.73E-02	1.8031	Up
RHPN1	3.66E-04	1.8021	Down
MST1	3.43E-03	1.8018	Down
CAPNS1	2.78E-03	1.8015	Up
MMAB	8.81E-04	1.8011	Down
ATP6V0A1	8.87E-03	1.8009	Down
CYP2U1	1.49E-03	1.8007	Down
LMLN	1.35E-04	1.8005	Down
REPIN1	2.30E-03	1.7998	Down
BEX4	1.31E-03	1.7998	Down
IL2RG	2.56E-04	1.7993	Up
GNAI1	5.03E-03	1.7984	Down
ZFP3	2.02E-02	1.7981	Down
LTB4R	6.53E-03	1.7968	Up
KLF5	2.48E-02	1.7968	Down
RECK	4.23E-03	1.7963	Down
SLCO3A1	2.35E-03	1.7955	Up

BAK1	5.29E-03	1.7955	Up
SYDE2	1.17E-02	1.7949	Down
CDC42BPB	3.56E-03	1.7948	Down
KLHDC1	7.35E-04	1.7947	Down
CPT1A	4.49E-02	1.7945	Up
CDKN2C	2.93E-02	1.7937	Up
ZNF404	2.25E-02	1.7929	Down
CUBN	9.17E-03	1.7912	Down
ADAT1	4.23E-04	1.7910	Up
PMVK	2.59E-03	1.7891	Up
MTPN	1.08E-03	1.7891	Up
PLEKHO2	3.46E-02	1.7884	Up
CD55	2.07E-02	1.7879	Down
CASZ1	1.22E-02	1.7879	Up
BNIP3	9.16E-03	1.7879	Down
C9orf72	1.31E-03	1.7875	Down
SUSD3	3.26E-05	1.7874	Down
ZFYVE28	9.27E-03	1.7870	Up
ZNF550	6.29E-04	1.7868	Down
FBXO45	4.22E-04	1.7851	Up
SLFN13	2.35E-03	1.7848	Up
CAMK4	2.32E-03	1.7844	Down
FAM129B	4.03E-03	1.7833	Up
TMEM181	2.03E-03	1.7829	Up
RNF157	2.16E-02	1.7815	Down
GEMIN7	5.43E-04	1.7802	Up
LLGL2	2.21E-03	1.7802	Up
MAP2K3	1.50E-03	1.7794	Up
GAPDH	7.25E-03	1.7772	Up
FAM184A	1.42E-02	1.7759	Down
PRR24	3.35E-02	1.7758	Down
ELMO2	1.05E-04	1.7756	Up
STARD5	2.94E-02	1.7754	Down
THRA	7.79E-03	1.7749	Down
TFAP4	3.48E-05	1.7743	Down
E2F3	1.92E-03	1.7737	Up
GK5	1.11E-03	1.7734	Up
CAPZA1	7.15E-04	1.7728	Up
ARHGAP11B	1.09E-02	1.7723	Up
DEPDC7	7.15E-05	1.7720	Down
CD82	1.28E-03	1.7706	Up
DHRS7	2.40E-02	1.7701	Up
TTC24	3.15E-02	1.7695	Down
ATP6V1D	5.07E-03	1.7693	Up
PTPN18	7.03E-03	1.7677	Up
TRIM59	1.47E-02	1.7673	Up
INPP1	1.67E-02	1.7652	Up
APH1B	1.31E-02	1.7650	Down

NINJ2	2.27E-02	1.7643	Up
TAGLN2	6.15E-04	1.7633	Up
PEX7	1.08E-02	1.7621	Down
ARPC1B	7.59E-03	1.7619	Up
KIAA1324	2.73E-04	1.7619	Up
LRFN4	2.53E-02	1.7616	Up
PTCH1	2.86E-02	1.7598	Up
AGMAT	3.43E-03	1.7593	Down
MSC	4.22E-02	1.7587	Up
RAP2B	9.26E-03	1.7579	Up
PPP1R18	1.49E-02	1.7566	Up
SCPEP1	4.50E-03	1.7554	Down
FAM102A	1.84E-04	1.7546	Down
RMND5B	7.66E-04	1.7529	Down
TTC39C	3.67E-03	1.7524	Up
HYI	6.36E-04	1.7523	Down
TMEM204	7.54E-03	1.7518	Down
PTPN9	9.96E-03	1.7510	Up
SLC17A9	1.09E-02	1.7501	Down
STMN3	1.05E-02	1.7493	Down
A1BG	6.10E-03	1.7490	Down
LDHA	1.57E-02	1.7472	Up
OSTF1	3.41E-03	1.7463	Up
RCAN3	3.00E-04	1.7460	Down
SCAI	1.26E-03	1.7459	Down
RIN1	4.97E-02	1.7450	Down
GPR146	1.91E-03	1.7449	Down
PPP1CA	1.85E-02	1.7431	Up
GPR171	2.78E-02	1.7425	Up
PAIP2B	2.40E-02	1.7402	Down
APOBEC3F	7.60E-03	1.7397	Up
TPM4	5.41E-04	1.7384	Up
NDFIP1	1.85E-03	1.7360	Down
MORN2	3.06E-04	1.7355	Down
COTL1	5.16E-03	1.7347	Up
TCTN1	9.14E-04	1.7346	Down
SSR3	6.16E-04	1.7340	Up
PDE7A	8.09E-04	1.7335	Down
ZC2HC1A	3.45E-02	1.7320	Up
CDH24	1.56E-03	1.7311	Down
ARRB1	1.54E-03	1.7303	Down
MICB	2.36E-03	1.7297	Up
PDZD11	8.03E-03	1.7287	Up
STRADB	3.35E-02	1.7277	Down
ODF2L	3.70E-04	1.7266	Up
IRF1	7.65E-03	1.7255	Up
CEP78	5.34E-03	1.7251	Up
USB1	2.23E-03	1.7244	Up

KATNAL1	7.22E-03	1.7234	Up
RASA4	1.28E-02	1.7234	Down
FAM45A	1.42E-03	1.7232	Up
PUS10	1.26E-02	1.7220	Up
PDE3B	1.22E-02	1.7205	Down
MEGF6	1.51E-02	1.7203	Down
KCNMB4	3.12E-04	1.7196	Down
CARD16	2.48E-03	1.7196	Up
TNIP1	7.17E-05	1.7193	Up
CELF6	1.61E-03	1.7184	Down
C19orf38	2.95E-02	1.7177	Down
ZBTB49	5.98E-03	1.7166	Up
MPZL1	7.51E-03	1.7163	Down
ACOT7	4.38E-02	1.7161	Up
PI4K2A	3.27E-02	1.7150	Up
IL17RA	8.57E-03	1.7145	Down
SFXN3	3.60E-05	1.7145	Up
C17orf67	2.94E-02	1.7144	Down
BOD1	1.27E-02	1.7135	Down
C16orf87	7.94E-03	1.7130	Up
HEXDC	4.91E-03	1.7129	Down
CISD3	3.79E-03	1.7125	Up
PTPN4	2.59E-03	1.7102	Up
HENMT1	2.55E-02	1.7098	Up
DIP2C	4.66E-02	1.7097	Down
LCP1	5.44E-03	1.7090	Up
RAB37	4.83E-02	1.7085	Up
PRPF40B	1.23E-02	1.7074	Down
CAST	9.53E-05	1.7070	Up
OTUD7B	2.86E-05	1.7056	Up
AL592284.1	1.04E-03	1.7050	Down
TIGD7	1.25E-03	1.7046	Down
EPS15	2.59E-03	1.7037	Up
SP140	3.18E-02	1.7025	Up
MPP7	1.20E-03	1.7019	Down
CDNF	7.80E-04	1.7002	Down
PLCD1	3.99E-03	1.7001	Up
IPCEF1	1.83E-02	1.6998	Down
CREG1	2.71E-02	1.6982	Down
LIN9	2.00E-02	1.6969	Down
KLHL5	8.93E-03	1.6962	Up
ACBD4	3.22E-04	1.6961	Down
IDS	4.72E-05	1.6958	Up
C11orf35	4.82E-03	1.6954	Down
MKNK1	2.92E-04	1.6948	Up
BLVRA	1.75E-03	1.6947	Up
EMP3	1.45E-03	1.6946	Up
CD79B	4.05E-02	1.6939	Up

CCND2	1.08E-03	1.6937	Up
TMEM60	1.93E-02	1.6931	Up
ZBED5	1.17E-02	1.6918	Down
CYP2E1	9.71E-04	1.6906	Up
RECQL5	1.88E-05	1.6905	Down
SLA	3.65E-03	1.6903	Up
MT2A	2.85E-02	1.6897	Up
KIAA0247	1.14E-04	1.6876	Up
MTRF1	1.56E-03	1.6871	Down
JAM3	3.16E-02	1.6863	Down
ST7	1.18E-03	1.6860	Up
FKBP2	1.52E-03	1.6857	Up
MACF1	2.76E-03	1.6853	Up
ZNF546	3.04E-02	1.6834	Down
RGS9	4.09E-02	1.6833	Up
RNF138	1.24E-02	1.6832	Down
MIEN1	8.85E-03	1.6816	Up
GCLM	2.35E-03	1.6811	Up
KLHL2	7.86E-04	1.6808	Down
HSPB1	2.13E-02	1.6798	Down
NCAPG2	3.48E-02	1.6796	Up
MDFIC	6.48E-03	1.6794	Up
USP44	4.74E-02	1.6791	Down
TUBG2	6.59E-03	1.6785	Down
ZNF30	2.95E-02	1.6781	Down
CRLF3	7.31E-05	1.6779	Down
CTSB	7.59E-04	1.6777	Up
WNT10B	8.10E-04	1.6775	Up
ZNRF2	9.31E-04	1.6770	Up
CRTC1	3.13E-03	1.6769	Down
CEP70	1.96E-02	1.6767	Down
PGAM1	1.05E-02	1.6761	Up
CYSLTR2	6.05E-03	1.6756	Down
IZUMO4	1.62E-02	1.6751	Down
ACVR1B	4.61E-03	1.6738	Down
FAM171A1	1.05E-02	1.6738	Down
RPL39L	4.21E-02	1.6736	Up
MLKL	2.14E-03	1.6735	Up
ZNF518B	1.69E-03	1.6728	Down
HIC2	1.78E-02	1.6725	Down
METAP1D	5.21E-03	1.6709	Down
MBLAC2	1.88E-02	1.6709	Down
TMEM184B	4.26E-04	1.6707	Up
FAM156B	9.79E-05	1.6704	Down
SPCS2	3.50E-03	1.6704	Up
NOS3	3.74E-02	1.6703	Down
SEL1L3	1.06E-02	1.6691	Up
RIPK3	1.23E-03	1.6686	Up

ACACB	8.80E-04	1.6679	Down
KBTBD8	4.04E-03	1.6677	Up
RMDN2	1.62E-02	1.6665	Down
CHP1	3.15E-03	1.6662	Up
TEX2	9.12E-03	1.6658	Down
ZNF441	2.35E-03	1.6647	Down
ALPK1	2.26E-02	1.6645	Down
ABHD17C	4.43E-03	1.6645	Up
HOOK2	2.16E-02	1.6640	Down
SULT1A1	6.19E-03	1.6639	Down
PFN2	2.80E-02	1.6639	Down
CPD	3.24E-03	1.6633	Up
TTC22	2.82E-02	1.6630	Up
C6orf1	9.79E-03	1.6624	Up
MSRB2	1.25E-02	1.6616	Down
BDH1	1.06E-03	1.6600	Down
LPP	1.19E-04	1.6598	Up
KTI12	1.62E-03	1.6596	Down
PPIF	3.36E-02	1.6576	Up
TATDN1	1.29E-03	1.6564	Down
ZNF80	6.41E-03	1.6549	Up
GPR25	1.15E-03	1.6549	Up
CLN8	3.90E-03	1.6544	Down
ANKRD26	6.03E-03	1.6524	Down
DNAI2	5.86E-03	1.6523	Up
MAP4K1	5.02E-03	1.6523	Up
PFN1	1.13E-02	1.6521	Up
TSSK6	3.59E-02	1.6514	Down
TMEM102	2.15E-02	1.6501	Up
TOR3A	9.37E-03	1.6487	Up
FOXP1	9.05E-04	1.6486	Down
DNAJC3	6.79E-03	1.6483	Up
SEC11C	3.31E-03	1.6479	Up
IFIT5	2.40E-02	1.6479	Down
NUDT17	3.76E-02	1.6475	Down
UBE2A	8.86E-05	1.6474	Up
CALM3	1.10E-03	1.6473	Down
MPP5	1.76E-03	1.6469	Down
SALL2	6.20E-04	1.6456	Down
DDX31	3.19E-02	1.6456	Down
EMB	7.51E-03	1.6445	Up
DPP4	3.62E-02	1.6444	Up
RASA2	1.28E-04	1.6441	Down
EHD1	1.48E-02	1.6437	Up
CHD7	2.62E-02	1.6433	Down
EOGT	8.46E-05	1.6428	Up
C11orf31	6.26E-03	1.6425	Down
C10orf35	9.16E-03	1.6420	Down

RASGRP2	2.95E-04	1.6413	Down
EFNA1	4.92E-02	1.6412	Down
FGFRL1	5.93E-04	1.6409	Up
GOLGA6L9	1.02E-02	1.6405	Up
PHF21A	1.10E-04	1.6403	Up
RRAS	5.63E-03	1.6402	Down
SLC10A3	3.78E-03	1.6402	Up
CD248	2.16E-02	1.6397	Down
RAB9A	9.92E-03	1.6397	Up
ZNF444	6.02E-03	1.6378	Down
ACTB	1.76E-02	1.6377	Up
ARL6IP1	2.85E-05	1.6367	Up
VCPIP1	7.65E-03	1.6367	Up
MMP23B	1.36E-02	1.6367	Up
BNIP3L	3.36E-06	1.6363	Down
PGAM4	2.42E-02	1.6360	Up
TBXA2R	5.68E-03	1.6356	Down
ZNF740	3.48E-03	1.6356	Down
CEP41	1.15E-02	1.6355	Down
PEA15	4.67E-04	1.6350	Up
PRKCD	1.66E-02	1.6349	Up
C14orf182	2.83E-02	1.6341	Down
ZCWPW1	2.08E-03	1.6340	Down
PIP4K2A	2.66E-03	1.6339	Up
HK2	4.61E-02	1.6330	Down
RPS13	1.13E-05	1.6327	Down
IL16	3.12E-03	1.6313	Down
STRBP	1.04E-03	1.6313	Down
TOM1L2	4.97E-05	1.6312	Down
CFLAR	7.88E-05	1.6309	Up
C1orf172	5.28E-03	1.6307	Down
OSBPL7	1.71E-03	1.6306	Up
UTRN	1.05E-03	1.6303	Up
DIP2B	1.26E-03	1.6301	Down
DIAPH1	1.05E-03	1.6299	Up
IFT140	6.31E-03	1.6294	Down
RAPGEF6	4.15E-03	1.6293	Down
MFSD12	3.96E-03	1.6290	Down
ITPK1	1.42E-03	1.6289	Up
ETV6	3.44E-03	1.6277	Up
AKTIP	3.89E-03	1.6271	Down
SLC39A8	1.03E-02	1.6268	Up
TCF3	4.23E-03	1.6267	Down
ANKRD16	5.92E-03	1.6266	Down
TPM2	2.61E-02	1.6257	Down
EML4	2.26E-02	1.6256	Up
ZRANB2	2.16E-02	1.6252	Down
MIB1	2.03E-03	1.6244	Up

CTSF	1.06E-02	1.6244	Down
DHODH	3.00E-03	1.6243	Down
C4orf32	2.00E-02	1.6239	Down
N6AMT1	5.04E-03	1.6234	Down
DOK2	6.95E-03	1.6233	Up
ENGASE	2.34E-03	1.6231	Down
LIG1	1.78E-03	1.6230	Down
ZNRF1	3.97E-03	1.6229	Up
PYCR2	3.99E-03	1.6227	Down
SIK3	2.18E-04	1.6222	Up
LEAP2	1.96E-02	1.6220	Down
ZNF395	1.31E-03	1.6220	Down
SMAP1	6.11E-04	1.6220	Up
TCEA2	2.97E-04	1.6200	Down
DOK6	1.73E-02	1.6200	Up
ZMAT1	8.94E-03	1.6197	Down
SERPINI1	9.90E-03	1.6190	Up
ELP3	1.14E-02	1.6188	Up
SYNJ2	1.72E-02	1.6184	Down
VWA5A	9.49E-03	1.6184	Down
DOPEY2	3.60E-05	1.6180	Up
TMEM8B	4.05E-03	1.6160	Down
LYSMD4	8.64E-05	1.6159	Down
GCH1	3.54E-02	1.6147	Up
PAOX	3.37E-02	1.6143	Up
SERPINB8	4.29E-04	1.6143	Up
TCP11L1	1.26E-02	1.6143	Up
RAB18	2.63E-03	1.6140	Up
VASH1	4.81E-06	1.6139	Up
HELZ	1.75E-04	1.6123	Up
MKL2	8.22E-03	1.6120	Down
ZNF512B	4.58E-04	1.6112	Down
VIMP	6.40E-03	1.6105	Up
AGBL2	1.65E-03	1.6103	Down
SLC20A1	3.89E-03	1.6102	Up
RIC3	4.17E-02	1.6097	Down
ZDHHC18	1.46E-04	1.6096	Up
TNFSF8	3.77E-02	1.6090	Up
NBPF3	8.21E-04	1.6083	Down
CASP4	2.91E-03	1.6076	Up
SLC19A1	1.73E-02	1.6063	Down
LRRC33	3.93E-02	1.6060	Up
ZNF681	4.62E-03	1.6058	Down
TPP1	9.33E-03	1.6054	Up
TSPAN4	4.33E-03	1.6051	Down
ARF1	1.72E-03	1.6049	Up
LAX1	2.06E-02	1.6049	Up
CHRNE	9.69E-03	1.6048	Up

CFL1	3.37E-03	1.6038	Up
MSH2	1.93E-02	1.6034	Down
GTF2IRD2	1.82E-03	1.6029	Down
SPIN3	4.27E-02	1.6014	Down
YPEL2	1.63E-02	1.6012	Down
TMEM50A	5.55E-03	1.6011	Up
DEGS1	5.10E-04	1.6009	Up
FAM156A	2.36E-04	1.5998	Down
MANF	8.37E-04	1.5982	Up
ST13	1.99E-03	1.5981	Down
AKIRIN2	6.69E-03	1.5970	Up
TTC14	2.22E-02	1.5967	Down
SETMAR	1.62E-02	1.5962	Down
PARP15	1.05E-03	1.5960	Up
SPSB1	1.88E-03	1.5959	Up
TTC12	9.89E-03	1.5958	Down
NIPA1	2.72E-02	1.5956	Up
LRCH4	9.26E-04	1.5956	Down
DNAJB11	1.34E-02	1.5951	Up
SERTAD2	9.00E-03	1.5939	Down
GP1BA	7.15E-03	1.5938	Down
CEP68	1.35E-02	1.5938	Down
ATM	3.42E-04	1.5931	Down
CTSA	3.74E-03	1.5921	Up
ZNF347	1.04E-02	1.5917	Down
NSDHL	1.06E-03	1.5910	Up
APOL3	2.86E-03	1.5909	Up
ZMYND8	2.77E-04	1.5908	Down
GDI1	2.43E-04	1.5907	Up
NIPAL2	7.95E-03	1.5894	Up
VDAC1	6.21E-03	1.5869	Up
CCDC106	2.77E-05	1.5868	Down
KRTCAP3	1.35E-02	1.5868	Down
TMEM9B	5.83E-03	1.5864	Up
KSR1	1.64E-02	1.5862	Up
AACS	9.94E-03	1.5856	Down
TMED8	1.07E-02	1.5851	Up
LRRC57	5.59E-03	1.5850	Up
LRRC20	7.53E-04	1.5847	Down
ZNF189	2.07E-02	1.5845	Down
BLM	4.29E-03	1.5843	Up
S100PBP	6.01E-03	1.5837	Up
RAB8B	2.20E-02	1.5836	Up
CRTC3	8.47E-04	1.5836	Down
ANKS6	1.96E-05	1.5828	Down
CDKN2AIP	6.03E-04	1.5826	Down
COX5A	4.29E-02	1.5823	Up
MYD88	5.07E-03	1.5821	Up

HOXB4	4.57E-02	1.5818	Up
NFKBIL1	1.44E-02	1.5805	Down
PET112	4.07E-02	1.5804	Down
TYSND1	1.68E-02	1.5800	Down
ARHGDIA	1.80E-02	1.5800	Up
FAIM3	1.09E-04	1.5799	Down
DPH5	4.94E-03	1.5793	Down
PKIG	5.15E-03	1.5793	Down
AKIP1	3.69E-02	1.5788	Up
ZNF879	1.76E-02	1.5787	Down
TJP2	1.01E-03	1.5787	Down
ST8SIA1	2.01E-02	1.5777	Up
SELT	1.32E-03	1.5765	Up
CDK2AP2	4.60E-02	1.5764	Up
DNHD1	2.81E-03	1.5759	Down
ELK3	4.41E-02	1.5757	Up
PAPSS1	3.77E-02	1.5757	Up
CLN5	1.21E-02	1.5757	Down
MAP3K4	6.99E-04	1.5751	Up
CHSY1	2.51E-03	1.5744	Up
GNG2	2.66E-03	1.5734	Up
DCPS	1.25E-02	1.5730	Up
WDYHV1	3.25E-02	1.5724	Up
PLOD3	2.07E-02	1.5722	Up
ZNF605	3.24E-02	1.5719	Down
MSN	4.39E-03	1.5718	Up
TUT1	5.43E-05	1.5713	Down
TLR2	1.81E-02	1.5711	Down
CHCHD5	1.51E-02	1.5711	Up
KEAP1	3.39E-02	1.5710	Up
P4HB	8.35E-03	1.5709	Up
PHLDB3	4.59E-02	1.5705	Down
MAP4K4	5.64E-04	1.5699	Down
GTF2IRD2B	4.99E-03	1.5699	Down
AKAP7	7.83E-05	1.5696	Down
DSTN	2.55E-04	1.5696	Up
ZNF141	3.01E-03	1.5695	Down
LPCAT4	2.54E-02	1.5695	Up
STPG1	5.80E-03	1.5690	Down
TTC9	4.95E-02	1.5685	Down
SMC4	4.32E-03	1.5684	Up
ITGA3	2.82E-02	1.5683	Up
KCNAB3	1.09E-03	1.5682	Down
SFT2D2	6.48E-04	1.5682	Up
KDM8	6.66E-03	1.5681	Down
PPP2R5C	1.43E-03	1.5675	Up
APOBEC3D	2.28E-02	1.5662	Up
PYGM	4.56E-03	1.5662	Down

C1QTNF6	1.35E-03	1.5661	Down
PSMB2	2.26E-03	1.5660	Up
AC006132.1	9.63E-03	1.5657	Up
OAF	3.01E-02	1.5656	Down
ZNF274	4.17E-04	1.5654	Down
NEK7	2.00E-02	1.5648	Up
P2RX7	1.34E-02	1.5647	Up
PATZ1	2.36E-04	1.5641	Down
SELM	3.82E-03	1.5637	Down
NLE1	4.23E-02	1.5636	Down
CASP7	3.15E-03	1.5636	Up
ZNF780B	2.54E-02	1.5633	Down
PBXIP1	7.54E-05	1.5632	Up
RPS6KA3	7.21E-03	1.5629	Up
RPN1	1.96E-03	1.5628	Up
FANCI	4.83E-03	1.5622	Up
SLFN12L	2.11E-03	1.5620	Up
GEMIN4	8.78E-04	1.5615	Down
RPS5	2.58E-05	1.5609	Down
CAPN1	1.54E-02	1.5606	Up
WDR19	2.52E-03	1.5604	Down
ZNF813	1.91E-02	1.5593	Down
ARL15	3.89E-03	1.5592	Up
PLA2G12A	1.34E-02	1.5582	Down
UAP1L1	4.24E-03	1.5577	Down
CHPF	3.53E-02	1.5575	Up
TRIM47	4.51E-02	1.5570	Up
PBX4	1.33E-02	1.5566	Up
KAT2A	3.92E-04	1.5559	Down
CLINT1	8.71E-03	1.5558	Up
MARK4	1.20E-02	1.5555	Up
ACRBP	2.70E-02	1.5547	Down
PDIA3	4.37E-03	1.5527	Up
EDA	1.05E-03	1.5519	Down
ACTG1	1.49E-02	1.5519	Up
NOL11	5.13E-04	1.5518	Down
ATP10A	3.80E-02	1.5518	Down
CSRP1	5.11E-03	1.5513	Up
FAM3A	1.05E-03	1.5511	Down
ZNF540	2.46E-02	1.5507	Down
MTMR2	7.40E-04	1.5500	Up
SNX5	6.03E-03	1.5497	Up
TESPA1	2.33E-02	1.5491	Down
RHOH	3.64E-04	1.5490	Down
SEPN1	1.81E-03	1.5490	Down
TAF1B	1.63E-02	1.5488	Up
CANX	2.11E-03	1.5486	Up
DGKH	3.17E-02	1.5485	Up

MINK1	1.47E-03	1.5478	Up
ZNF497	2.42E-02	1.5477	Down
ZNF485	8.89E-03	1.5475	Down
PLCB3	3.58E-02	1.5457	Up
EIF5A2	5.11E-03	1.5455	Up
HEMK1	3.11E-02	1.5453	Down
TFRC	3.92E-03	1.5449	Up
B3GNT2	3.30E-03	1.5446	Up
TBC1D4	2.76E-02	1.5439	Down
ARF3	3.25E-03	1.5429	Up
DTX1	2.94E-02	1.5425	Down
SERINC5	5.25E-03	1.5421	Down
ITK	1.47E-04	1.5420	Down
RPL21	2.13E-04	1.5419	Down
ERCC6L2	2.88E-04	1.5409	Down
UBASH3B	7.09E-04	1.5405	Down
TPD52	1.68E-02	1.5404	Up
C5orf63	4.89E-02	1.5403	Down
FAM50A	2.16E-03	1.5402	Up
NRIP1	3.73E-02	1.5399	Down
SLC7A6	7.58E-03	1.5399	Down
TPM1	2.08E-02	1.5395	Down
RNF168	1.83E-02	1.5390	Up
CYBA	1.73E-02	1.5384	Up
RPS3A	6.46E-04	1.5381	Down
TNK2	4.19E-03	1.5372	Down
MUC20	2.98E-02	1.5370	Down
TNFAIP8L1	2.33E-04	1.5367	Down
SH2D1A	2.10E-02	1.5364	Up
NSUN5	7.22E-03	1.5355	Down
MTERFD2	5.78E-04	1.5349	Down
ACVR2B	3.91E-02	1.5349	Down
DHX58	2.18E-03	1.5347	Down
NCR3LG1	3.16E-03	1.5346	Up
TM7SF2	3.35E-03	1.5344	Down
SLC7A6OS	3.61E-02	1.5340	Down
EDEM2	1.70E-03	1.5340	Up
SERPINF1	2.44E-02	1.5339	Down
HIVEP3	2.61E-02	1.5336	Up
ATP6V0E2	1.98E-03	1.5335	Down
RNF149	3.65E-03	1.5334	Up
ACBD5	4.43E-03	1.5333	Up
AIM1	7.87E-03	1.5325	Up
RMDN3	1.22E-03	1.5310	Up
DECR2	2.83E-02	1.5309	Down
EXD2	3.11E-03	1.5309	Down
PELI3	8.68E-04	1.5298	Down
LYRM4	2.27E-02	1.5294	Down

CLCN2	7.18E-04	1.5294	Down
PEX3	2.40E-02	1.5293	Down
EIF3E	3.71E-03	1.5293	Down
SDF2L1	4.40E-02	1.5286	Up
SYPL1	7.76E-03	1.5285	Down
PPIB	4.28E-03	1.5284	Up
CDK19	7.15E-05	1.5278	Up
FBXW8	8.01E-03	1.5270	Down
RALGPS1	1.34E-02	1.5269	Up
FOXO3	5.38E-03	1.5269	Up
ZNHIT2	1.19E-02	1.5268	Down
KAT8	2.60E-03	1.5260	Down
DCAF7	1.62E-02	1.5259	Up
NCKIPSD	1.03E-02	1.5253	Down
FOXP3	4.58E-02	1.5252	Up
FOXO1	7.18E-04	1.5252	Down
ANKS3	4.16E-03	1.5242	Down
MAGEH1	5.79E-03	1.5238	Down
ZNF500	2.47E-03	1.5238	Down
TMED9	4.00E-03	1.5238	Up
LYST	3.31E-02	1.5233	Up
UBQLN4	4.09E-02	1.5228	Up
RASSF2	7.95E-04	1.5223	Up
DYRK4	7.18E-03	1.5223	Down
RETSAT	4.11E-02	1.5220	Up
NEDD4	3.79E-02	1.5216	Up
MMP19	5.32E-04	1.5212	Down
CD28	8.48E-03	1.5210	Up
PCBP4	2.23E-02	1.5206	Up
SAT2	2.43E-02	1.5203	Down
TARBP1	2.62E-03	1.5200	Down
UNG	3.97E-02	1.5186	Up
EMC7	2.43E-03	1.5183	Up
KPTN	8.64E-03	1.5181	Down
SIT1	4.82E-02	1.5159	Up
ELOVL1	3.72E-03	1.5158	Up
YWHAZ	8.90E-04	1.5154	Up
TUBA1C	3.07E-02	1.5154	Up
ABHD5	3.13E-03	1.5151	Up
SVIL	1.61E-02	1.5149	Down
DERL1	5.47E-04	1.5149	Up
C21orf2	3.21E-03	1.5149	Down
PDSS1	1.92E-02	1.5146	Down
PDE6B	7.21E-03	1.5145	Down
RPS6KL1	1.35E-04	1.5141	Down
ZCCHC6	5.59E-03	1.5140	Up
PRKCB	1.82E-02	1.5137	Up
GIMAP2	1.25E-02	1.5136	Down

MTRR	1.95E-03	1.5129	Up
LRMP	3.28E-04	1.5128	Down
HSD11B1L	3.19E-02	1.5127	Down
SLC2A11	2.11E-02	1.5123	Down
ZNF580	4.35E-02	1.5123	Down
TMEM243	4.68E-04	1.5123	Down
SFXN2	1.44E-02	1.5118	Down
RPL31	7.32E-06	1.5113	Down
PGAP2	7.69E-04	1.5112	Down
NOTCH1	2.09E-02	1.5110	Down
TBCB	6.04E-03	1.5109	Up
TCTEX1D2	1.44E-02	1.5104	Up
ZNF287	3.33E-02	1.5103	Down
PPP2CA	7.99E-03	1.5102	Up
PSMB5	2.04E-02	1.5098	Down
ZNF573	4.52E-03	1.5097	Down
PITPNA	3.13E-03	1.5094	Up
C21orf91	1.53E-02	1.5092	Up
SH3GLB1	2.07E-04	1.5091	Up
ZNF419	3.57E-03	1.5087	Down
SPTBN1	9.30E-04	1.5087	Down
PSMB9	3.67E-02	1.5086	Up
ZNF862	3.89E-02	1.5082	Down
ST8SIA4	3.80E-02	1.5077	Up
SPCS3	1.11E-04	1.5074	Up
CDK2	2.88E-02	1.5068	Down
NBPF1	3.02E-04	1.5061	Down
APOM	4.71E-02	1.5058	Down
ASPH	8.77E-04	1.5055	Down
HSP90AA1	4.36E-04	1.5048	Up
EEF1G	6.95E-06	1.5047	Down
SMIM14	1.27E-03	1.5042	Up
PSTPIP1	4.65E-04	1.5042	Up
ANKRD32	1.45E-02	1.5041	Up
FAM135A	5.14E-03	1.5037	Down
TTC7A	1.65E-03	1.5037	Up
ZFP62	2.82E-02	1.5035	Down
GNA13	4.65E-03	1.5032	Up
PXMP2	4.72E-02	1.5028	Up
UMPS	9.58E-03	1.5026	Down
RPS6	4.12E-04	1.5023	Down
RPL22	1.46E-03	1.5022	Down
FSTL3	1.10E-02	1.5020	Down
AGPAT2	4.82E-02	1.5020	Up
FAM126A	2.07E-02	1.5019	Up

Table S3: List of differentially expressed genes (826) comparing CD161^{+} Treg to CD161^{-} Treg with *P*<0.05 and fold change ≥1.5

Gene symbol	*P*-value	Fold change	Up/Down
KLRB1	1.26E-06	172.2297	Up
COL5A3	8.01E-04	23.8095	Up
FCER1G	9.20E-04	12.5644	Down
NOG	4.69E-04	11.8050	Down
APBA2	2.54E-04	11.3065	Down
RORC	7.81E-04	10.9854	Up
AIF1	1.71E-03	10.6097	Down
CEACAM4	2.33E-04	10.4224	Down
FXYD2	6.93E-04	9.2964	Down
GPR25	4.44E-05	8.5780	Up
CTSH	6.44E-04	8.2194	Up
CCR9	2.22E-04	7.2053	Up
LTK	9.31E-04	7.1599	Up
LAG3	4.26E-06	7.0177	Up
PLEKHG3	1.78E-03	6.9440	Up
AK5	1.01E-03	6.8174	Down
PLXND1	8.08E-04	6.5104	Up
FLT4	3.95E-03	6.3799	Down
PTGDS	2.53E-04	6.2690	Up
AUTS2	1.43E-03	6.2071	Up
CCR2	3.67E-04	6.1070	Up
IGF1R	2.87E-03	6.0833	Down
AIM2	4.13E-04	5.8585	Up
IL1R1	1.91E-03	5.8261	Up
EPHA1	4.00E-04	5.8214	Down
CCL20	3.18E-05	5.7371	Up
SLC7A8	2.61E-03	5.4765	Up
SCML1	1.13E-02	5.3450	Down
PTMS	4.16E-04	5.2596	Up
MAF	7.08E-04	5.2563	Up
TBXAS1	1.01E-02	4.9709	Up
VNN2	2.46E-03	4.9653	Down
BHLHE40	4.46E-02	4.6529	Up
LMO4	3.52E-03	4.5780	Up
GCNT4	1.98E-03	4.5574	Down
FAM174B	1.13E-03	4.4848	Up
RNF144A	3.30E-03	4.4799	Down
RASGRP4	5.13E-03	4.4346	Up
ZSCAN18	4.74E-03	4.4285	Down
C3AR1	7.05E-04	4.3968	Up
CITED4	1.84E-03	4.3693	Down
SMCO4	7.51E-06	4.3653	Up
ITGAM	1.32E-04	4.3479	Up
COL18A1	7.52E-04	4.3400	Down
POU2AF1	4.03E-04	4.3287	Up

RNF175	1.19E-02	4.2928	Down
ACTN1	3.70E-03	4.1642	Down
GOLGA8O	1.73E-03	4.1478	Down
SWAP70	4.65E-03	4.1460	Down
RTKN2	6.94E-04	4.0562	Down
PDCD1	6.43E-03	4.0435	Up
TNFRSF13C	7.37E-04	4.0232	Down
SELP	5.40E-03	4.0213	Down
TCEA3	1.02E-02	3.9860	Down
RRM2	1.30E-02	3.9457	Up
CDKN2A	8.02E-04	3.8470	Up
AURKB	1.72E-03	3.8428	Up
DCHS1	3.90E-04	3.8319	Down
GADD45G	1.47E-02	3.7978	Up
CCR5	3.33E-03	3.7060	Up
RNF130	1.83E-03	3.6929	Down
LIMS3L	7.77E-04	3.6849	Up
LIMS3	7.77E-04	3.6849	Up
ADAM12	2.86E-02	3.6269	Up
HLF	1.78E-04	3.5910	Up
HOPX	1.90E-02	3.5532	Up
CD300A	8.46E-05	3.5218	Up
TYMS	1.84E-02	3.5136	Up
CCR6	3.18E-02	3.4888	Up
GNA15	1.29E-02	3.4549	Up
E2F2	2.34E-03	3.4502	Up
INPP1	1.27E-03	3.4479	Up
EEPD1	4.14E-03	3.4478	Up
SLC22A17	2.77E-02	3.4457	Down
LIMS1	6.47E-04	3.4351	Up
MIR1260B	1.02E-02	3.4062	Down
SLC14A1	7.06E-03	3.3658	Down
SERPINB6	4.84E-02	3.3604	Down
CENPV	9.03E-04	3.3496	Down
CXCR6	3.37E-02	3.3398	Up
MT1E	1.04E-02	3.3383	Up
ADAM19	1.68E-03	3.3225	Up
GPRASP1	6.04E-04	3.3131	Down
MYBL2	1.05E-02	3.2931	Up
ASB2	1.72E-04	3.2670	Up
HOMER3	7.78E-03	3.2239	Up
BCAS4	5.77E-04	3.2208	Down
USP46	7.76E-04	3.2136	Up
GPR55	4.00E-03	3.2109	Down
RMI2	1.95E-03	3.2053	Up
FURIN	4.76E-04	3.2015	Up
CDT1	6.04E-03	3.1911	Up
LEF1	5.72E-04	3.1844	Down

SFMBT2	2.88E-04	3.1831	Down
SYTL2	8.92E-03	3.1737	Up
EDAR	8.44E-03	3.1704	Down
ANK1	2.71E-03	3.1702	Up
NOXA1	9.84E-04	3.1668	Down
TK1	8.64E-03	3.1647	Up
CD70	7.25E-03	3.0966	Up
SLC29A2	9.44E-04	3.0744	Down
MAPK11	3.31E-03	3.0510	Up
FRY	4.69E-04	3.0419	Up
TLE2	3.60E-04	3.0417	Down
MPP1	2.59E-03	3.0379	Down
GNLY	2.93E-04	3.0051	Up
RAD54B	7.61E-04	3.0038	Up
GPX1	1.93E-04	2.9974	Up
PDE9A	4.94E-02	2.9721	Down
AKAP7	6.16E-03	2.9572	Down
TNK1	2.27E-03	2.9514	Down
SMPD3	1.73E-03	2.9341	Down
IER3	2.53E-02	2.9332	Down
OBSCN	3.80E-03	2.9229	Down
LMO7	1.85E-03	2.8807	Down
SLC17A9	2.53E-03	2.8507	Down
GAL3ST4	5.70E-03	2.8458	Down
PTPN13	7.31E-03	2.8453	Up
ZNF626	1.65E-02	2.8299	Down
SORBS3	5.96E-03	2.8275	Down
NHSL2	3.95E-05	2.8260	Up
AGPAT4	1.28E-02	2.8061	Up
MYBL1	4.72E-03	2.7989	Up
MKI67	1.17E-03	2.7840	Up
GPC2	3.87E-03	2.7768	Down
CD38	1.45E-02	2.7676	Up
TOP2A	3.42E-02	2.7667	Up
CHD7	1.12E-02	2.7666	Down
GAS6	5.22E-04	2.7491	Up
LIF	9.49E-03	2.7474	Up
SPG20	8.47E-03	2.7444	Down
GAB2	2.35E-02	2.7430	Up
ZNRF1	1.82E-02	2.7304	Up
METRN	2.06E-03	2.7265	Down
SLC40A1	1.10E-02	2.7153	Down
TNFRSF4	4.38E-03	2.7118	Up
PPM1N	6.96E-03	2.7032	Down
SSTR3	1.71E-02	2.7017	Down
GPA33	6.97E-03	2.6891	Down
TNFRSF18	6.40E-03	2.6830	Up
NPDC1	3.96E-03	2.6817	Up

IRF5	1.38E-02	2.6752	Up
TSPAN2	5.14E-05	2.6695	Up
TPX2	5.23E-03	2.6683	Up
KRT18	2.29E-02	2.6627	Down
AMIGO1	3.98E-03	2.6577	Down
KIAA0101	9.46E-03	2.6501	Up
KLRG1	1.58E-02	2.6409	Up
SNPH	5.83E-03	2.6373	Down
SPSB1	1.42E-04	2.6268	Up
HRH4	3.62E-03	2.6142	Up
GPR183	4.98E-04	2.6139	Up
THEM5	2.98E-02	2.6138	Up
AMIGO2	1.85E-04	2.5990	Down
RGS12	8.22E-03	2.5977	Down
PTPRM	1.32E-02	2.5956	Up
ITGA4	3.83E-03	2.5908	Up
E2F1	8.56E-03	2.5862	Up
BLVRA	7.23E-05	2.5858	Up
AQP3	7.43E-04	2.5747	Up
MCF2L2	1.37E-02	2.5716	Up
TIMD4	2.34E-02	2.5641	Down
IKZF2	1.67E-03	2.5580	Down
ADAMTS10	4.21E-03	2.5497	Down
IFI44	3.41E-02	2.5178	Up
YWHAH	1.98E-03	2.5141	Up
NRIP1	3.65E-02	2.5130	Down
CEBPA	1.10E-02	2.5088	Up
GGT7	1.36E-03	2.5083	Down
MXRA7	5.73E-04	2.5023	Up
ID2	2.26E-04	2.5002	Up
TOX2	2.23E-02	2.4958	Up
VCL	1.96E-04	2.4945	Up
GAB3	3.49E-03	2.4887	Up
IL6ST	2.17E-02	2.4862	Down
ZNF506	1.19E-03	2.4770	Down
CD151	7.25E-03	2.4686	Up
AXIN2	3.23E-02	2.4674	Down
CD80	6.85E-03	2.4649	Up
CCNB2	7.98E-03	2.4538	Up
MYL6B	2.09E-03	2.4461	Up
RIMKLB	1.10E-02	2.4448	Down
S100A11	6.41E-03	2.4310	Up
CXorf57	1.61E-03	2.4292	Down
SULT1B1	4.08E-02	2.4103	Down
SUOX	3.43E-03	2.4092	Down
ZNF550	2.15E-03	2.4078	Down
VDR	3.05E-06	2.4027	Up
FAM153A	1.97E-02	2.4006	Down

SNTA1	2.06E-02	2.3913	Up
SYT11	1.88E-02	2.3906	Up
TMEM156	1.42E-03	2.3863	Up
CERKL	6.90E-03	2.3843	Up
MAML2	4.97E-03	2.3733	Down
PM20D2	1.04E-02	2.3691	Down
NLGN2	3.07E-03	2.3679	Down
PELI2	2.66E-02	2.3671	Down
PTCH1	5.16E-04	2.3667	Down
ARHGAP21	2.27E-02	2.3645	Down
LGMN	2.12E-03	2.3578	Up
HID1	1.01E-02	2.3559	Down
PDE3B	5.22E-03	2.3543	Down
MID2	1.04E-02	2.3482	Down
PDE1B	2.35E-02	2.3457	Up
CYFIP1	5.50E-05	2.3435	Up
TMEM30B	1.57E-02	2.3374	Down
CD200R1	2.88E-02	2.3351	Up
ARHGAP31	2.59E-04	2.3306	Up
NME4	1.56E-02	2.3261	Down
REEP3	1.07E-02	2.3252	Up
NCF2	5.01E-04	2.3240	Up
DDR1	1.29E-02	2.3193	Down
PROSAPIP1	2.00E-02	2.3103	Down
STRIP2	8.42E-05	2.3071	Up
BACH2	3.27E-02	2.3038	Down
FAM117B	3.00E-02	2.3037	Down
CEP68	1.02E-03	2.3029	Down
MYO1F	1.15E-02	2.3002	Up
RGPD1	7.80E-05	2.3001	Down
SNTB1	4.59E-02	2.2897	Down
PLEKHF1	3.35E-02	2.2882	Up
ARHGAP10	7.45E-03	2.2864	Up
NSG1	3.89E-02	2.2850	Up
NR3C2	1.65E-02	2.2778	Down
THEMIS	2.01E-02	2.2736	Up
ROPN1L	2.39E-04	2.2723	Up
TAF4B	2.32E-02	2.2719	Down
DUSP6	6.32E-04	2.2705	Up
ACSS2	1.58E-03	2.2613	Down
ADPRH	4.80E-02	2.2600	Up
CENPN	9.75E-03	2.2581	Up
C4orf48	9.10E-03	2.2547	Up
KIF11	1.66E-02	2.2546	Up
IKZF4	4.63E-02	2.2516	Down
PLEKHA5	3.59E-02	2.2516	Up
CNKSR2	2.62E-03	2.2507	Down
TROAP	1.40E-02	2.2488	Up

RGPD2	2.04E-04	2.2457	Down
WDR34	1.92E-02	2.2449	Up
CORO1C	1.80E-02	2.2426	Up
STOM	1.81E-03	2.2425	Up
CENPF	1.86E-04	2.2390	Up
POU6F1	1.41E-03	2.2387	Down
MGAT5	1.15E-03	2.2336	Up
SNN	3.46E-03	2.2218	Down
HOOK1	3.97E-02	2.2203	Down
PKD2	3.29E-03	2.2168	Up
CKS2	1.33E-02	2.2152	Up
PPP1R26	2.91E-02	2.2132	Up
GRAMD1B	2.41E-04	2.2102	Up
TRIM73	3.30E-02	2.2089	Down
CEBPD	4.01E-02	2.1916	Up
SLAMF1	3.71E-03	2.1894	Up
LYZ	2.56E-02	2.1880	Up
NUSAP1	2.66E-02	2.1833	Up
GOLGA7B	9.60E-04	2.1798	Down
OCM	9.95E-03	2.1606	Down
NPC1	9.31E-04	2.1562	Up
TXK	2.15E-02	2.1546	Down
ARNTL	1.50E-03	2.1509	Up
CEP70	1.24E-03	2.1506	Down
APP	8.38E-03	2.1450	Down
ZNF320	3.03E-03	2.1411	Down
ANXA4	3.52E-02	2.1397	Up
ZNF532	1.57E-02	2.1368	Down
GSAP	4.57E-03	2.1332	Down
ETS2	1.29E-03	2.1324	Down
CD40LG	4.62E-03	2.1306	Up
TNFRSF9	6.84E-04	2.1303	Down
PDGFB	1.35E-02	2.1265	Down
ASF1B	4.01E-02	2.1228	Up
CMTM6	2.54E-03	2.1210	Up
DFNB31	3.14E-02	2.1200	Down
SLC25A35	1.54E-02	2.1157	Up
DENND3	2.41E-02	2.1152	Up
FNDC3B	9.68E-03	2.1121	Down
ZNF486	3.74E-03	2.1093	Down
EPHX2	3.82E-02	2.1052	Down
ZCCHC14	2.03E-02	2.1042	Down
RNF207	9.74E-04	2.0944	Up
AIG1	7.24E-03	2.0915	Down
ARHGAP32	2.45E-02	2.0890	Down
SLC25A20	2.47E-04	2.0889	Up
SLC16A3	5.29E-03	2.0875	Up
ZNF737	2.48E-03	2.0855	Down

FAM105A	1.30E-03	2.0842	Down
CYSTM1	2.07E-02	2.0825	Up
ODF3B	2.94E-02	2.0802	Up
OSBPL1A	1.41E-03	2.0783	Up
PMVK	3.91E-04	2.0745	Up
ANKS6	2.19E-03	2.0656	Down
IRAK2	7.82E-04	2.0653	Up
ARHGEF39	2.27E-02	2.0600	Up
IMPACT	3.40E-02	2.0554	Up
CDKN2C	3.84E-02	2.0553	Up
RAB34	1.99E-02	2.0516	Down
ATP1B1	2.39E-02	2.0510	Up
CEP55	1.69E-02	2.0463	Up
CDC14B	2.83E-03	2.0454	Down
C7orf41	2.69E-03	2.0432	Down
ADA	1.36E-04	2.0423	Up
C1orf85	5.37E-04	2.0418	Up
RGMB	8.83E-05	2.0418	Down
ZNF485	9.08E-03	2.0367	Down
LRRC33	1.38E-02	2.0355	Up
RBPJ	2.72E-02	2.0345	Up
HIP1R	6.87E-03	2.0343	Down
SAMD3	1.26E-02	2.0338	Up
FCRL3	2.18E-02	2.0318	Down
CTSB	2.93E-04	2.0288	Up
EXD2	7.46E-03	2.0278	Down
OGFRL1	1.07E-02	2.0266	Up
PLXDC1	1.42E-02	2.0254	Down
TIAM1	3.80E-04	2.0240	Down
ERN1	1.15E-02	2.0233	Up
BLMH	7.40E-03	2.0232	Up
RAP1GAP2	5.56E-03	2.0168	Up
JAM3	2.46E-02	2.0145	Down
KIAA0895L	2.01E-03	2.0134	Up
NUF2	7.09E-03	2.0099	Up
GYLTL1B	1.78E-04	2.0070	Down
B4GALT5	8.96E-03	2.0024	Up
HVCN1	3.66E-02	2.0018	Down
FCGRT	3.95E-02	1.9988	Down
GATA3	3.55E-02	1.9987	Up
MAL	4.17E-03	1.9954	Down
WSB2	2.38E-04	1.9943	Up
CD59	2.07E-03	1.9926	Up
ZBTB18	2.42E-02	1.9923	Down
PAQR4	1.98E-02	1.9904	Up
PLTP	1.98E-02	1.9883	Up
PEPD	5.17E-03	1.9841	Up
RASSF2	7.00E-03	1.9786	Up

TBC1D2	2.69E-02	1.9784	Up
ACYP2	2.75E-02	1.9729	Up
BIRC5	4.57E-02	1.9708	Up
ACAA2	7.55E-03	1.9690	Up
LTBP3	6.04E-03	1.9652	Down
MLLT4	2.39E-02	1.9647	Up
SUSD1	6.16E-03	1.9634	Up
STARD9	8.09E-04	1.9613	Down
LMTK3	3.08E-03	1.9610	Down
BAIAP3	4.07E-02	1.9593	Up
ZNF585B	2.22E-02	1.9588	Down
PITPNM2	1.75E-03	1.9540	Down
GDE1	9.24E-04	1.9538	Up
TMEM8B	1.46E-03	1.9488	Down
OSTF1	2.10E-03	1.9476	Up
ARPC1B	6.45E-03	1.9463	Up
AGO4	4.80E-04	1.9425	Up
TIGIT	4.06E-02	1.9420	Down
ZNF329	7.28E-03	1.9395	Down
ZNF382	3.79E-04	1.9368	Down
TUFT1	1.04E-02	1.9291	Up
ARRDC5	1.58E-02	1.9268	Down
RILPL1	1.38E-02	1.9253	Down
CRIP1	3.71E-02	1.9232	Up
C3orf14	1.20E-02	1.9231	Up
RYK	2.44E-04	1.9191	Down
CD226	1.45E-02	1.9182	Up
SSBP4	2.47E-02	1.9174	Up
FGFR1	4.98E-02	1.9165	Up
MICAL2	3.78E-02	1.9114	Up
TRIM74	3.05E-02	1.9100	Down
ZNF420	1.58E-02	1.9065	Down
IMMP2L	1.53E-02	1.9061	Down
MRPL10	1.33E-03	1.9015	Up
ETV6	2.84E-04	1.8977	Up
AL592284.1	1.68E-02	1.8925	Down
CD72	4.40E-02	1.8917	Up
GKAP1	4.47E-02	1.8898	Down
OXNAD1	2.91E-02	1.8897	Down
SELM	3.17E-02	1.8867	Down
CD79B	2.89E-02	1.8866	Up
CRY1	1.67E-03	1.8846	Up
SLC25A5	5.73E-05	1.8831	Up
MAFG	1.47E-03	1.8803	Up
COTL1	1.50E-02	1.8788	Up
GALNT10	6.01E-03	1.8775	Up
PNP	1.03E-02	1.8773	Up
ANXA2	2.98E-02	1.8771	Up

METAP1D	4.78E-02	1.8733	Down
CCR4	4.64E-02	1.8731	Up
MAGEF1	2.09E-03	1.8728	Down
CCDC167	1.49E-02	1.8714	Up
CDC42BPG	3.40E-02	1.8702	Down
MLLT3	4.14E-02	1.8698	Down
GPSM1	3.69E-02	1.8694	Up
TMEM64	1.27E-02	1.8688	Up
FRMD4B	2.54E-02	1.8641	Up
GTDC1	4.81E-02	1.8641	Up
ZNF256	1.69E-02	1.8639	Down
B4GALT1	7.68E-03	1.8588	Up
HN1	2.91E-03	1.8564	Up
RUFY3	2.65E-03	1.8556	Down
FOXM1	1.33E-02	1.8552	Up
RABGAP1L	1.12E-03	1.8548	Up
GPR137B	3.11E-02	1.8510	Up
DBNDD2	2.80E-02	1.8496	Down
PHTF2	1.12E-02	1.8492	Up
CD82	4.88E-03	1.8474	Up
ZNF649	5.97E-03	1.8466	Down
GPRIN3	9.82E-03	1.8464	Up
CDK20	6.35E-03	1.8462	Down
RNF157	5.18E-03	1.8441	Down
PRR22	1.95E-03	1.8432	Down
TSHZ2	1.44E-02	1.8430	Up
NETO2	3.96E-02	1.8423	Up
AMN1	1.18E-02	1.8421	Down
UBE2T	3.78E-03	1.8398	Up
IL12RB1	1.08E-03	1.8397	Up
NBEAL2	3.17E-02	1.8378	Up
SLC4A5	1.16E-04	1.8376	Down
F2R	5.92E-03	1.8375	Up
ULK1	4.02E-03	1.8363	Up
EPHB4	1.31E-02	1.8360	Down
CERK	6.52E-04	1.8351	Up
ZC4H2	1.15E-02	1.8350	Down
NDC80	3.99E-02	1.8332	Up
FANCC	4.01E-03	1.8326	Down
TCF7	4.15E-02	1.8323	Down
KLHDC1	6.89E-04	1.8298	Down
OGDH	8.41E-03	1.8290	Up
AACS	1.68E-02	1.8274	Down
CNN2	4.16E-02	1.8258	Up
RHEBL1	7.75E-03	1.8246	Up
ATM	1.08E-02	1.8240	Down
DZIP3	2.14E-03	1.8227	Up
ZNF101	5.31E-03	1.8224	Down

FAS	7.47E-03	1.8203	Up
BAK1	2.40E-03	1.8152	Up
ZNF607	7.34E-03	1.8095	Down
S100A13	2.44E-02	1.8089	Up
CHEK1	2.93E-02	1.8088	Up
ZIK1	4.32E-02	1.8078	Down
DPP3	2.37E-03	1.8070	Up
CPPED1	4.83E-03	1.8058	Up
SCNN1D	9.58E-03	1.8057	Down
ZCWPW1	1.67E-02	1.8045	Down
HEMK1	1.48E-02	1.8034	Down
RASGRP3	1.01E-02	1.8023	Up
SLC2A11	1.37E-02	1.8023	Down
ABRACL	2.57E-03	1.8020	Up
CTNNA1	3.69E-02	1.8020	Up
TIGD7	3.23E-03	1.8007	Down
FAM84B	2.83E-02	1.7966	Down
CKS1B	7.97E-03	1.7961	Up
FAM216A	3.83E-02	1.7940	Down
UCP2	3.36E-03	1.7939	Up
C10orf2	2.30E-02	1.7935	Down
LZTFL1	3.74E-02	1.7930	Up
NCAPG2	3.14E-03	1.7929	Up
FBXO10	5.45E-03	1.7916	Up
ZNF493	2.49E-02	1.7885	Down
ZFP82	4.36E-02	1.7884	Down
KCNA2	1.55E-02	1.7880	Up
C15orf41	8.52E-03	1.7866	Down
RORA	8.95E-03	1.7856	Up
IKZF3	1.99E-02	1.7838	Up
C1orf109	1.33E-02	1.7797	Down
MAP3K4	1.17E-02	1.7793	Up
RASA2	1.96E-03	1.7778	Down
PDK1	3.72E-02	1.7774	Down
ZNF441	4.43E-02	1.7774	Down
MYH3	8.37E-03	1.7742	Down
ZNF274	3.09E-04	1.7737	Down
ZNF680	1.11E-02	1.7724	Down
MLF1IP	1.31E-02	1.7719	Up
RCBTB2	2.69E-02	1.7695	Down
SAV1	1.46E-02	1.7692	Down
SORL1	1.94E-03	1.7686	Down
EPM2A	4.85E-03	1.7684	Down
ZNF254	8.24E-03	1.7683	Down
TFDP1	1.12E-04	1.7660	Up
PLEKHB1	2.35E-02	1.7658	Down
TRMT11	9.75E-03	1.7651	Down
MB21D1	4.36E-03	1.7585	Up

LYRM5	1.58E-02	1.7574	Down
GALNT12	1.01E-02	1.7552	Down
TFAP4	2.34E-02	1.7545	Down
NAB1	1.92E-02	1.7533	Up
ACPL2	1.83E-02	1.7521	Down
KLF4	4.53E-03	1.7515	Up
RPS6KA2	3.90E-02	1.7487	Down
HEG1	7.31E-03	1.7486	Down
RECK	8.94E-04	1.7477	Down
DDX31	6.75E-03	1.7458	Down
SMYD5	7.16E-04	1.7447	Down
TM9SF1	4.53E-02	1.7437	Up
MBOAT1	3.94E-04	1.7430	Up
SFI1	5.87E-03	1.7429	Down
CAPN12	1.61E-02	1.7398	Up
SQRDL	1.25E-04	1.7392	Up
ARL3	3.19E-03	1.7382	Up
C16orf45	5.30E-03	1.7365	Down
TCTEX1D2	9.54E-03	1.7347	Up
ATP2B4	4.15E-02	1.7334	Up
SCAPER	5.46E-03	1.7327	Down
REC8	3.04E-02	1.7323	Up
FDXR	5.40E-03	1.7319	Up
RAB11FIP1	1.59E-02	1.7318	Up
ADRBK2	4.97E-04	1.7301	Down
ENO2	4.07E-03	1.7295	Down
SPEF2	1.07E-02	1.7265	Down
PHTF1	1.40E-02	1.7261	Up
CDK1	3.06E-02	1.7239	Up
TGFBR1	3.29E-03	1.7237	Down
ZNF136	1.09E-03	1.7229	Down
ZNF44	8.41E-03	1.7222	Down
GALC	5.76E-03	1.7212	Up
BCL2	2.38E-02	1.7184	Down
ZNF395	3.02E-03	1.7184	Down
ZNF880	8.08E-03	1.7149	Down
MYO5A	3.13E-03	1.7141	Up
DUSP16	3.63E-03	1.7132	Up
SNX2	7.40E-03	1.7131	Up
PSTPIP2	4.21E-04	1.7118	Down
FABP5	3.19E-02	1.7111	Up
PCNA	2.78E-03	1.7098	Up
ACTG1	2.83E-03	1.7079	Up
ACACB	1.88E-02	1.7065	Down
NCKAP5L	2.81E-02	1.7061	Down
TIMELESS	4.69E-03	1.7059	Up
ZNF528	4.58E-03	1.7023	Down
S100A4	2.92E-02	1.7022	Up

RELL1	3.57E-02	1.7021	Up
SH3BGRL3	2.03E-02	1.7010	Up
ZBTB32	1.45E-02	1.7007	Up
CDC42EP3	2.90E-02	1.6988	Up
FBXO45	7.05E-03	1.6981	Up
KAT2B	6.74E-03	1.6963	Up
ZNF721	1.93E-02	1.6963	Down
GSR	6.57E-03	1.6953	Up
ZNF768	3.93E-03	1.6949	Down
ZNF268	1.81E-02	1.6946	Down
FRMD4A	8.17E-03	1.6936	Up
USP53	2.09E-02	1.6912	Down
BAG2	9.98E-03	1.6907	Down
IL18R1	2.28E-02	1.6896	Up
RGPD4	3.62E-03	1.6868	Down
TRPC1	2.52E-03	1.6859	Down
METTL21B	2.40E-02	1.6850	Up
ANXA5	3.88E-03	1.6837	Up
MAN1A1	1.03E-02	1.6834	Up
MCOLN2	5.36E-03	1.6832	Up
MMAB	3.18E-03	1.6795	Down
NAP1L3	4.85E-02	1.6787	Down
CAPZB	1.64E-03	1.6784	Up
UBE2F	1.86E-02	1.6772	Up
TRAPPC1	3.95E-02	1.6769	Up
DOPEY2	6.81E-03	1.6768	Up
PTPN18	5.87E-03	1.6751	Up
ZNF93	2.22E-02	1.6749	Down
ZMAT1	3.61E-03	1.6743	Down
TXNDC17	5.39E-03	1.6740	Up
CHML	4.24E-02	1.6732	Down
CHP1	4.47E-03	1.6725	Up
PBX3	1.04E-02	1.6713	Down
RAB37	1.59E-02	1.6709	Up
BAG3	1.93E-02	1.6690	Up
TGFB3	2.90E-02	1.6674	Down
PPP1CA	3.72E-02	1.6673	Up
PPP4R1	4.52E-04	1.6672	Up
STRADB	3.87E-02	1.6670	Down
RACGAP1	9.56E-03	1.6663	Up
PFN1	8.59E-03	1.6657	Up
HEXDC	4.29E-02	1.6647	Down
ZNF658	4.24E-02	1.6639	Down
SLC10A3	9.37E-03	1.6632	Up
TJP2	4.51E-02	1.6631	Up
CELF6	8.04E-04	1.6615	Down
SLC25A37	5.69E-03	1.6611	Down
MAST4	1.03E-02	1.6597	Up

WARS2	6.07E-03	1.6592	Up
USP15	5.69E-03	1.6590	Down
MRC2	2.11E-02	1.6568	Up
ZNF813	2.58E-02	1.6568	Down
ZNF616	2.75E-02	1.6564	Down
CTPS2	3.41E-02	1.6537	Down
C9orf16	3.35E-02	1.6536	Up
HLTF	2.64E-03	1.6533	Down
ATP8A1	7.95E-03	1.6528	Down
CD58	4.26E-02	1.6526	Up
BBS2	2.73E-02	1.6518	Down
ZDHHC12	2.70E-02	1.6509	Up
STX11	1.93E-02	1.6488	Up
THG1L	3.14E-02	1.6484	Down
DPY19L1	1.75E-02	1.6469	Up
FAM13A	4.21E-03	1.6456	Down
ATP6V0D1	1.30E-02	1.6444	Up
ZNF681	1.39E-03	1.6436	Down
C12orf57	2.02E-02	1.6435	Down
TGFB1	4.35E-02	1.6421	Up
TPD52	8.40E-03	1.6412	Up
ASNA1	3.84E-03	1.6402	Up
ZNF568	2.05E-02	1.6388	Down
ANKRD13B	3.79E-02	1.6385	Up
GALM	3.65E-02	1.6385	Up
CCDC141	2.13E-02	1.6381	Down
SLC16A10	3.65E-02	1.6377	Down
KIAA1328	1.69E-02	1.6369	Down
PHACTR2	2.27E-02	1.6368	Up
SPON2	4.21E-02	1.6358	Up
SIL1	4.82E-03	1.6357	Up
RNASEL	4.47E-02	1.6338	Down
ZNF549	1.68E-02	1.6337	Down
TPK1	3.17E-03	1.6321	Up
CDC20	1.42E-02	1.6318	Up
ZNF814	1.75E-02	1.6314	Down
ZBP1	4.01E-02	1.6312	Up
RAP2B	1.30E-03	1.6310	Up
MOCS3	6.44E-03	1.6297	Down
TXN	9.34E-03	1.6285	Up
AP1S1	4.41E-02	1.6283	Up
PIK3IP1	6.56E-03	1.6265	Down
GTF3C6	6.37E-03	1.6264	Up
OASL	3.80E-02	1.6257	Up
TTC12	2.07E-03	1.6239	Down
C1orf112	1.09E-03	1.6212	Up
RCN3	2.32E-02	1.6205	Down
SETD6	9.17E-03	1.6202	Down

C5orf30	1.55E-02	1.6187	Up
CECR1	2.02E-02	1.6182	Down
LSR	4.38E-02	1.6177	Down
ZNF91	2.70E-02	1.6174	Down
C6orf48	4.34E-03	1.6168	Down
PLIN2	8.53E-03	1.6161	Up
LDOC1	4.48E-02	1.6149	Up
HSF2	3.51E-02	1.6142	Down
C16orf87	9.78E-04	1.6139	Up
ATF7IP2	3.05E-02	1.6134	Up
GEN1	4.09E-03	1.6126	Up
IL18RAP	3.34E-02	1.6122	Up
CFL2	1.74E-02	1.6119	Up
ABCC4	1.75E-02	1.6116	Down
CHST12	2.91E-02	1.6116	Up
HSDL2	3.25E-02	1.6111	Up
ZNF554	1.90E-02	1.6097	Down
CA11	1.66E-02	1.6091	Down
RHOG	3.36E-02	1.6088	Up
ELMO2	8.32E-04	1.6086	Up
TAF1A	4.15E-02	1.6082	Down
MAPK3	2.74E-02	1.6077	Up
PIEZO1	3.09E-02	1.6068	Up
ACSM3	4.59E-02	1.6059	Down
CEP170	7.96E-03	1.6041	Down
ZNF662	6.43E-03	1.6033	Down
ZC3H12D	3.22E-02	1.6023	Up
PSMB3	8.57E-04	1.6015	Up
PCED1B	3.70E-02	1.6009	Down
TMEM38B	2.87E-02	1.6003	Down
DET1	3.85E-03	1.5997	Down
STARD4	4.93E-02	1.5997	Up
NADK	1.92E-02	1.5996	Up
ERCC6L2	2.84E-03	1.5995	Down
PIM2	2.00E-02	1.5992	Up
ATP6V1D	8.47E-03	1.5952	Up
SFR1	5.35E-03	1.5949	Down
CD28	4.76E-02	1.5948	Up
COLQ	2.31E-02	1.5946	Up
Sep-08	2.87E-02	1.5934	Up
BOD1	2.38E-03	1.5929	Down
MPP5	4.15E-02	1.5928	Down
ZNF302	3.18E-02	1.5919	Down
LPAR6	4.52E-02	1.5914	Down
CDK2AP1	2.02E-02	1.5912	Up
KIAA1147	1.28E-03	1.5905	Down
FKBP11	3.49E-02	1.5900	Up
ARHGAP11A	1.30E-03	1.5895	Up

RAPGEF1	1.79E-02	1.5894	Up
ZMAT3	2.75E-02	1.5893	Up
PGAM1	1.05E-02	1.5890	Up
GCC2	2.38E-02	1.5886	Down
EFHD2	4.16E-02	1.5884	Up
ARRB1	1.70E-02	1.5879	Down
CMSS1	1.87E-02	1.5878	Down
CFL1	8.15E-03	1.5871	Up
DCUN1D4	3.73E-03	1.5854	Down
FLNA	1.83E-02	1.5852	Up
PILRA	3.39E-02	1.5850	Down
ZNF605	2.53E-02	1.5833	Down
AMPD2	2.10E-02	1.5802	Up
PLXNC1	4.50E-02	1.5792	Up
ACTB	3.05E-02	1.5786	Up
ZNF16	3.71E-03	1.5778	Down
ACVR2A	1.74E-02	1.5778	Up
LAMTOR2	9.60E-03	1.5757	Up
FANCI	1.27E-02	1.5744	Up
TPM4	2.41E-02	1.5742	Up
ACTN4	4.75E-02	1.5723	Up
PHF21A	1.21E-03	1.5721	Up
BRMS1L	5.37E-03	1.5718	Down
ERO1LB	1.06E-02	1.5717	Down
IQCE	1.86E-02	1.5713	Up
PCK2	4.25E-02	1.5702	Up
WDR27	8.61E-03	1.5697	Down
SESN2	1.01E-02	1.5693	Up
NDUFB3	7.47E-03	1.5688	Up
ARHGAP11B	1.67E-02	1.5671	Up
DCTN3	3.81E-03	1.5669	Up
MIB1	7.98E-03	1.5664	Up
RAPGEF2	3.89E-02	1.5661	Up
RNF216	9.31E-04	1.5659	Down
PBXIP1	7.77E-04	1.5636	Up
TRIM4	1.24E-03	1.5632	Down
RAD51	3.80E-02	1.5616	Up
ZNF763	1.77E-03	1.5616	Down
SH2D1A	7.16E-03	1.5613	Up
TCTN2	2.22E-02	1.5610	Down
ETHE1	1.07E-02	1.5599	Up
C11orf1	8.88E-03	1.5598	Down
TTC3	6.12E-03	1.5597	Down
ZFP14	2.93E-03	1.5591	Down
CD6	9.76E-04	1.5584	Up
NUDT1	3.28E-02	1.5582	Up
LRIF1	1.75E-02	1.5577	Down
FAM53B	2.47E-02	1.5572	Up

ARPC5	1.90E-02	1.5564	Up
FAM135A	2.87E-03	1.5564	Down
LMLN	3.48E-04	1.5548	Down
IL4R	1.54E-02	1.5545	Down
TPCN1	3.16E-02	1.5543	Down
ABCD1	3.26E-02	1.5543	Up
RPL26L1	2.19E-02	1.5519	Up
XKR8	5.83E-03	1.5507	Up
PGAM4	8.11E-03	1.5480	Up
NSMF	3.48E-02	1.5479	Down
TIMP1	1.73E-02	1.5472	Up
ZNF627	1.16E-02	1.5464	Down
IFITM2	3.97E-02	1.5455	Up
SYNJ2	4.08E-03	1.5443	Up
CAMK4	5.50E-03	1.5432	Down
PRDX5	1.42E-02	1.5431	Up
ZNF354B	5.35E-03	1.5430	Down
SCP2	1.31E-03	1.5426	Up
TRADD	1.91E-02	1.5419	Up
TSPAN5	2.52E-02	1.5418	Up
GPHN	4.68E-02	1.5417	Down
SARS2	3.46E-02	1.5416	Up
MIEN1	2.86E-03	1.5413	Up
TMOD2	4.91E-02	1.5409	Down
ZNF841	1.03E-02	1.5404	Down
MAK16	4.50E-02	1.5403	Down
S1PR4	3.93E-02	1.5401	Up
WDFY2	1.24E-02	1.5394	Up
MTRR	4.49E-03	1.5392	Up
ZNF347	8.41E-03	1.5390	Down
SLC12A6	6.14E-03	1.5385	Down
GSE1	4.08E-02	1.5375	Up
S100A10	2.87E-02	1.5370	Up
ZNF480	1.02E-02	1.5366	Down
RAB27A	3.62E-02	1.5356	Up
SERTAD3	4.69E-02	1.5356	Up
ARF3	7.81E-04	1.5355	Up
KLF9	7.03E-03	1.5348	Down
CD81	8.43E-03	1.5337	Up
ZNF14	2.60E-02	1.5333	Down
PRKAG2	1.30E-03	1.5332	Down
OCM2	3.08E-03	1.5331	Down
TMEM25	2.33E-02	1.5310	Down
SEC14L1	1.48E-04	1.5310	Up
SPAG16	4.37E-02	1.5298	Down
ZNF124	1.13E-02	1.5295	Down
BIRC3	4.55E-04	1.5284	Down
TMBIM1	1.34E-03	1.5273	Up

PCYOX1L	7.88E-04	1.5267	Up
NIPSNAP3A	2.29E-02	1.5264	Up
C1orf216	4.79E-02	1.5262	Up
U2AF1L4	1.90E-02	1.5261	Up
PYGM	2.23E-02	1.5258	Down
TMEM60	7.57E-03	1.5254	Up
PRDX6	2.90E-03	1.5245	Down
PPIF	2.87E-02	1.5239	Up
PKP4	2.12E-02	1.5234	Up
EMP3	2.15E-02	1.5231	Up
PLCL1	3.21E-02	1.5231	Down
TIMM17B	3.26E-03	1.5223	Up
RNF215	4.53E-02	1.5221	Up
SPIN3	7.14E-03	1.5218	Down
TRIM44	2.24E-02	1.5207	Down
YWHAQ	5.21E-03	1.5206	Up
TET2	9.71E-03	1.5196	Up
ORAI3	4.45E-02	1.5193	Up
RPS6KA3	1.22E-03	1.5193	Up
CHAF1B	3.62E-02	1.5190	Up
MYL6	3.73E-03	1.5184	Up
H2AFV	7.62E-05	1.5179	Up
NMUR1	7.65E-06	1.5179	Up
LRRFIP1	3.09E-03	1.5173	Up
NCALD	6.13E-03	1.5171	Up
UBAP1	1.17E-02	1.5168	Up
NDUFB10	2.44E-02	1.5161	Up
UBE2L6	7.63E-03	1.5132	Up
SGOL1	1.49E-02	1.5124	Up
RCN1	2.69E-02	1.5115	Up
DHODH	2.80E-02	1.5108	Down
CDK5	4.33E-02	1.5101	Up
TUBB	8.02E-03	1.5101	Up
ZNF682	8.67E-03	1.5100	Down
THEMIS2	2.75E-02	1.5100	Up
TTL	2.33E-02	1.5092	Up
LAPTM5	2.87E-03	1.5091	Up
SLC9A1	2.96E-02	1.5091	Up
ANO6	4.97E-03	1.5088	Up
RPS12	5.18E-03	1.5087	Down
FGFRL1	4.33E-02	1.5080	Down
ATP11C	1.32E-02	1.5066	Down
MACROD1	4.56E-02	1.5066	Down
ZNF567	5.33E-04	1.5060	Down
ANKRD39	3.06E-02	1.5059	Up
CHMP2A	2.58E-04	1.5054	Up
MTPN	1.89E-02	1.5054	Up
BEX4	4.07E-02	1.5052	Down

AGPHD1	3.30E-02	1.5051	Down
SH2D2A	1.99E-02	1.5047	Up
ZNF816	4.40E-02	1.5044	Down
TMEM184B	3.75E-02	1.5038	Up
REV1	4.00E-03	1.5027	Down
DNHD1	4.06E-03	1.5021	Down
SLC37A1	1.77E-02	1.5017	Down
C17orf49	4.87E-04	1.5015	Up
DNAI2	5.50E-03	1.5010	Up
TTC27	2.26E-02	1.5009	Down
ZNF512	3.78E-02	1.5008	Down
UBE2H	1.77E-02	1.5006	Up
CAMKK2	1.64E-02	1.5001	Up

Table S4: Significantly altered pathways within CD161$^+$ Tconv signature as identified using Ingenuity Pathway analysis (IPA)

**Ingenuity Canonical Pathways**	**-log(*P*-value)**	***P*-value**	**Ratio**
Molecular Mechanisms of Cancer	8.36	4.37E-09	1.78E-01
T Helper Cell Differentiation	7.97	1.07E-08	3.28E-01
Protein Kinase A Signaling	6.73	1.86E-07	1.65E-01
Germ Cell-Sertoli Cell Junction Signaling	6.31	4.90E-07	2.12E-01
Integrin Signaling	6.26	5.50E-07	1.96E-01
Regulation of Cellular Mechanics by Calpain Protease	5.88	1.32E-06	3.09E-01
Leukocyte Extravasation Signaling	5.88	1.32E-06	1.92E-01
Epithelial Adherens Junction Signaling	5.72	1.91E-06	2.10E-01
Actin Cytoskeleton Signaling	5.39	4.07E-06	1.81E-01
RhoA Signaling	5.31	4.90E-06	2.17E-01
Role of Macrophages, Fibroblasts and Endothelial Cells in Rheumatoid Arthritis	4.90	1.26E-05	1.60E-01
PI3K/AKT Signaling	4.75	1.78E-05	2.07E-01
Apoptosis Signaling	4.55	2.82E-05	2.27E-01
Death Receptor Signaling	4.32	4.79E-05	2.20E-01
nNOS Signaling in Neurons	4.18	6.61E-05	2.83E-01
Sphingosine-1-phosphate Signaling	4.16	6.92E-05	2.04E-01
Sertoli Cell-Sertoli Cell Junction Signaling	4.04	9.12E-05	1.73E-01
p70S6K Signaling	4.01	9.77E-05	1.95E-01
Hepatic Fibrosis / Hepatic Stellate Cell Activation	3.68	2.09E-04	1.66E-01
NF-κB Activation by Viruses	3.56	2.75E-04	2.19E-01
Factors Promoting Cardiogenesis in Vertebrates	3.47	3.39E-04	2.02E-01
Melatonin Signaling	3.41	3.89E-04	2.21E-01
RhoGDI Signaling	3.34	4.57E-04	1.63E-01
Ephrin Receptor Signaling	3.34	4.57E-04	1.63E-01
Eicosanoid Signaling	3.33	4.68E-04	2.26E-01
PPARα/RXRα Activation	3.28	5.25E-04	1.64E-01
Cardiac Hypertrophy Signaling	3.17	6.76E-04	1.50E-01
PTEN Signaling	3.16	6.92E-04	1.78E-01
Production of Nitric Oxide and Reactive Oxygen Species in Macrophages	3.06	8.71E-04	1.56E-01
Virus Entry via Endocytic Pathways	3.02	9.55E-04	1.91E-01
Crosstalk between Dendritic Cells and Natural Killer Cells	3.02	9.55E-04	1.91E-01
Tec Kinase Signaling	2.99	1.02E-03	1.61E-01
MSP-RON Signaling Pathway	2.95	1.12E-03	2.39E-01
PI3K Signaling in B Lymphocytes	2.92	1.20E-03	1.71E-01
fMLP Signaling in Neutrophils	2.90	1.26E-03	1.78E-01
Role of Tissue Factor in Cancer	2.90	1.26E-03	1.78E-01
Axonal Guidance Signaling	2.88	1.32E-03	1.26E-01
HIPPO signaling	2.80	1.58E-03	1.88E-01
Superpathway of Inositol Phosphate Compounds	2.80	1.58E-03	1.51E-01

Colorectal Cancer Metastasis Signaling	2.80	1.58E-03	1.43E-01
B Cell Receptor Signaling	2.70	2.00E-03	1.52E-01
Glioblastoma Multiforme Signaling	2.70	2.00E-03	1.59E-01
Rac Signaling	2.70	2.00E-03	1.75E-01
UVA-Induced MAPK Signaling	2.69	2.04E-03	1.84E-01
G-Protein Coupled Receptor Signaling	2.66	2.19E-03	1.38E-01
D-myo-inositol-5-phosphate Metabolism	2.63	2.34E-03	1.59E-01
iNOS Signaling	2.63	2.34E-03	2.33E-01
VEGF Signaling	2.59	2.57E-03	1.80E-01
Tumoricidal Function of Hepatic Natural Killer Cells	2.57	2.69E-03	2.92E-01
Type I Diabetes Mellitus Signaling	2.56	2.75E-03	1.70E-01
Chondroitin Sulfate Biosynthesis (Late Stages)	2.55	2.82E-03	2.27E-01
Role of NFAT in Cardiac Hypertrophy	2.53	2.95E-03	1.48E-01
Endothelin-1 Signaling	2.50	3.16E-03	1.49E-01
Synaptic Long Term Potentiation	2.48	3.31E-03	1.64E-01
14-3-3-mediated Signaling	2.48	3.31E-03	1.64E-01
GPCR-Mediated Nutrient Sensing in Enteroendocrine Cells	2.48	3.31E-03	1.81E-01
Thrombin Signaling	2.46	3.47E-03	1.44E-01
NF-κB Signaling	2.46	3.47E-03	1.48E-01
Chemokine Signaling	2.44	3.63E-03	1.91E-01
nNOS Signaling in Skeletal Muscle Cells	2.38	4.17E-03	3.57E-01
Synaptic Long Term Depression	2.37	4.27E-03	1.54E-01
Breast Cancer Regulation by Stathmin1	2.37	4.27E-03	1.42E-01
FAK Signaling	2.33	4.68E-03	1.74E-01
Ceramide Signaling	2.23	5.89E-03	1.75E-01
Sperm Motility	2.21	6.17E-03	1.58E-01
ERK/MAPK Signaling	2.20	6.31E-03	1.40E-01
Signaling by Rho Family GTPases	2.20	6.31E-03	1.33E-01
Ephrin B Signaling	2.17	6.76E-03	1.78E-01
Paxillin Signaling	2.17	6.76E-03	1.63E-01
Cellular Effects of Sildenafil (Viagra)	2.16	6.92E-03	1.53E-01
Remodeling of Epithelial Adherens Junctions	2.11	7.76E-03	1.82E-01
ILK Signaling	2.08	8.32E-03	1.38E-01
Induction of Apoptosis by HIV1	2.06	8.71E-03	1.86E-01
Chondroitin and Dermatan Biosynthesis	2.02	9.55E-03	5.00E-01
Chondroitin Sulfate Biosynthesis	2.01	9.77E-03	1.92E-01
Macropinocytosis Signaling	2.01	9.77E-03	1.76E-01
phagosome formation	2.00	1.00E-02	1.57E-01
Wnt/β-catenin Signaling	1.97	1.07E-02	1.39E-01
3-phosphoinositide Degradation	1.95	1.12E-02	1.44E-01
Role of Osteoblasts, Osteoclasts and Chondrocytes in Rheumatoid Arthritis	1.95	1.12E-02	1.31E-01
Regulation of IL-2 Expression in Activated and Anergic T Lymphocytes	1.93	1.17E-02	1.67E-01
3-phosphoinositide Biosynthesis	1.92	1.20E-02	1.41E-01
HGF Signaling	1.92	1.20E-02	1.54E-01
D-myo-inositol (1,4,5,6)-Tetrakisphosphate	1.91	1.23E-02	1.48E-01

Biosynthesis			
D-myo-inositol (3,4,5,6)-tetrakisphosphate Biosynthesis	1.91	1.23E-02	1.48E-01
GPCR-Mediated Integration of Enteroendocrine Signaling Exemplified by an L Cell	1.91	1.23E-02	1.71E-01
Regulation of Actin-based Motility by Rho	1.91	1.23E-02	1.61E-01
Caveolar-mediated Endocytosis Signaling	1.86	1.38E-02	1.69E-01
PEDF Signaling	1.86	1.38E-02	1.69E-01
CXCR4 Signaling	1.86	1.38E-02	1.39E-01
IGF-1 Signaling	1.85	1.41E-02	1.55E-01
ERK5 Signaling	1.85	1.41E-02	1.75E-01
Dermatan Sulfate Biosynthesis	1.84	1.45E-02	1.82E-01
Gluconeogenesis I	1.83	1.48E-02	2.40E-01
Pregnenolone Biosynthesis	1.81	1.55E-02	4.29E-01
Actin Nucleation by ARP-WASP Complex	1.79	1.62E-02	1.79E-01
Adipogenesis pathway	1.78	1.66E-02	1.43E-01
p38 MAPK Signaling	1.77	1.70E-02	1.45E-01
Triacylglycerol Biosynthesis	1.77	1.70E-02	2.12E-01
Phospholipase C Signaling	1.77	1.70E-02	1.26E-01
GADD45 Signaling	1.76	1.74E-02	2.63E-01
1D-myo-inositol Hexakisphosphate Biosynthesis II (Mammalian)	1.76	1.74E-02	2.63E-01
Gαq Signaling	1.76	1.74E-02	1.38E-01
HMGB1 Signaling	1.74	1.82E-02	1.44E-01
Granulocyte Adhesion and Diapedesis	1.73	1.86E-02	1.33E-01
IL-15 Signaling	1.71	1.95E-02	1.67E-01
Cholecystokinin/Gastrin-mediated Signaling	1.70	2.00E-02	1.49E-01
Role of NFAT in Regulation of the Immune Response	1.70	2.00E-02	1.33E-01
Dopamine-DARPP32 Feedback in cAMP Signaling	1.69	2.04E-02	1.34E-01
SAPK/JNK Signaling	1.67	2.14E-02	1.51E-01
CCR5 Signaling in Macrophages	1.66	2.19E-02	1.64E-01
Agrin Interactions at Neuromuscular Junction	1.66	2.19E-02	1.64E-01
NRF2-mediated Oxidative Stress Response	1.66	2.19E-02	1.30E-01
cAMP-mediated signaling	1.66	2.19E-02	1.25E-01
Xenobiotic Metabolism Signaling	1.65	2.24E-02	1.21E-01
α-Adrenergic Signaling	1.64	2.29E-02	1.53E-01
Stearate Biosynthesis I (Animals)	1.63	2.34E-02	2.00E-01
CD27 Signaling in Lymphocytes	1.63	2.34E-02	1.76E-01
IL-12 Signaling and Production in Macrophages	1.62	2.40E-02	1.37E-01
Dendritic Cell Maturation	1.62	2.40E-02	1.30E-01
CCR3 Signaling in Eosinophils	1.60	2.51E-02	1.42E-01
TNFR2 Signaling	1.60	2.51E-02	2.14E-01
Relaxin Signaling	1.59	2.57E-02	1.36E-01
TGF-β Signaling	1.56	2.75E-02	1.49E-01
Reelin Signaling in Neurons	1.53	2.95E-02	1.52E-01

IL-3 Signaling	1.49	3.24E-02	1.55E-01
Pathogenesis of Multiple Sclerosis	1.48	3.31E-02	3.33E-01
GNRH Signaling	1.46	3.47E-02	1.34E-01
Neuropathic Pain Signaling In Dorsal Horn Neurons	1.46	3.47E-02	1.41E-01
Altered T Cell and B Cell Signaling in Rheumatoid Arthritis	1.45	3.55E-02	1.48E-01
P2Y Purigenic Receptor Signaling Pathway	1.45	3.55E-02	1.36E-01
Spermine and Spermidine Degradation I	1.43	3.72E-02	5.00E-01
Glutathione Redox Reactions II	1.43	3.72E-02	5.00E-01
Leptin Signaling in Obesity	1.42	3.80E-02	1.51E-01
CDP-diacylglycerol Biosynthesis I	1.41	3.89E-02	2.50E-01
Androgen Signaling	1.41	3.89E-02	1.36E-01
Angiopoietin Signaling	1.38	4.17E-02	1.54E-01
CREB Signaling in Neurons	1.38	4.17E-02	1.24E-01
Ephrin A Signaling	1.37	4.27E-02	1.67E-01
Calcium Signaling	1.35	4.47E-02	1.24E-01
Calcium Transport I	1.35	4.47E-02	3.00E-01
Histidine Degradation VI	1.35	4.47E-02	3.00E-01
Human Embryonic Stem Cell Pluripotency	1.35	4.47E-02	1.30E-01
Superpathway of D-myo-inositol (1,4,5)-trisphosphate Metabolism	1.35	4.47E-02	2.08E-01
Glycolysis I	1.35	4.47E-02	2.08E-01
Glioma Invasiveness Signaling	1.35	4.47E-02	1.58E-01
Role of PKR in Interferon Induction and Antiviral Response	1.35	4.47E-02	1.75E-01
Gap Junction Signaling	1.34	4.57E-02	1.26E-01
Regulation of eIF4 and p70S6K Signaling	1.32	4.79E-02	1.27E-01
Calcium-induced T Lymphocyte Apoptosis	1.31	4.90E-02	1.55E-01
Myc Mediated Apoptosis Signaling	1.31	4.90E-02	1.55E-01
Retinoic acid Mediated Apoptosis Signaling	1.31	4.90E-02	1.55E-01

© 2000-2016 QIAGEN. All rights reserved.

Table S5: Significantly altered pathways within CD161^{+} Treg signature as identified using Ingenuity Pathway analysis (IPA)

**Ingenuity Canonical Pathways**	**-log(*P*-value)**	***P*-value**	**Ratio**
Epithelial Adherens Junction Signaling	5.58	2.63E-06	1.40E-01
Cell Cycle: G2/M DNA Damage Checkpoint Regulation	5.31	4.90E-06	2.24E-01
T Helper Cell Differentiation	4.67	2.14E-05	1.79E-01
Hepatic Fibrosis / Hepatic Stellate Cell Activation	4.06	8.71E-05	1.10E-01
Actin Cytoskeleton Signaling	4.06	8.71E-05	1.05E-01
RhoA Signaling	3.78	1.66E-04	1.25E-01
Germ Cell-Sertoli Cell Junction Signaling	3.47	3.39E-04	1.09E-01
Integrin Signaling	3.22	6.03E-04	9.79E-02
Tight Junction Signaling	3.16	6.92E-04	1.02E-01
GADD45 Signaling	3.02	9.55E-04	2.63E-01
Adenine and Adenosine Salvage III	2.86	1.38E-03	5.00E-01
Purine Ribonucleosides Degradation to Ribose-1-phosphate	2.86	1.38E-03	5.00E-01
Molecular Mechanisms of Cancer	2.83	1.48E-03	7.80E-02
Cyclins and Cell Cycle Regulation	2.83	1.48E-03	1.30E-01
Pancreatic Adenocarcinoma Signaling	2.76	1.74E-03	1.13E-01
Remodeling of Epithelial Adherens Junctions	2.75	1.78E-03	1.36E-01
ILK Signaling	2.74	1.82E-03	9.39E-02
Protein Kinase A Signaling	2.64	2.29E-03	7.57E-02
Sertoli Cell-Sertoli Cell Junction Signaling	2.55	2.82E-03	9.25E-02
Leukocyte Extravasation Signaling	2.45	3.55E-03	8.81E-02
STAT3 Pathway	2.44	3.63E-03	1.23E-01
Rac Signaling	2.37	4.27E-03	1.07E-01
VEGF Signaling	2.36	4.37E-03	1.12E-01
Cell Cycle: G1/S Checkpoint Regulation	2.31	4.90E-03	1.27E-01
Chronic Myeloid Leukemia Signaling	2.26	5.50E-03	1.09E-01
Glioma Signaling	2.19	6.46E-03	1.06E-01
Altered T Cell and B Cell Signaling in Rheumatoid Arthritis	2.14	7.24E-03	1.11E-01
p53 Signaling	2.07	8.51E-03	1.02E-01
Molybdenum Cofactor Biosynthesis	1.99	1.02E-02	5.00E-01
Signaling by Rho Family GTPases	1.98	1.05E-02	7.73E-02
Pyrimidine Deoxyribonucleotides De Novo Biosynthesis I	1.97	1.07E-02	1.90E-01
p38 MAPK Signaling	1.96	1.10E-02	9.40E-02
Myc Mediated Apoptosis Signaling	1.96	1.10E-02	1.21E-01
Regulation of Actin-based Motility by Rho	1.95	1.12E-02	1.03E-01
PTEN Signaling	1.94	1.15E-02	9.32E-02
ATM Signaling	1.92	1.20E-02	1.19E-01
PAK Signaling	1.92	1.20E-02	1.02E-01
MSP-RON Signaling Pathway	1.90	1.26E-02	1.30E-01
Cellular Effects of Sildenafil (Viagra)	1.79	1.62E-02	8.87E-02
ERK5 Signaling	1.77	1.70E-02	1.11E-01

Role of BRCA1 in DNA Damage Response	1.76	1.74E-02	1.03E-01
Regulation of IL-2 Expression in Activated and Anergic T Lymphocytes	1.76	1.74E-02	1.03E-01
Estrogen-mediated S-phase Entry	1.76	1.74E-02	1.67E-01
DNA Double-Strand Break Repair by Homologous Recombination	1.71	1.95E-02	2.14E-01
nNOS Signaling in Skeletal Muscle Cells	1.71	1.95E-02	2.14E-01
Nur77 Signaling in T Lymphocytes	1.70	2.00E-02	1.18E-01
Paxillin Signaling	1.64	2.29E-02	9.18E-02
Unfolded protein response	1.62	2.40E-02	1.13E-01
Cardiac Hypertrophy Signaling	1.60	2.51E-02	7.27E-02
Adenosine Nucleotides Degradation II	1.55	2.82E-02	1.88E-01
Role of CHK Proteins in Cell Cycle Checkpoint Control	1.55	2.82E-02	1.09E-01
Regulation of Cellular Mechanics by Calpain Protease	1.55	2.82E-02	1.09E-01
TGF-β Signaling	1.51	3.09E-02	9.20E-02
Autoimmune Thyroid Disease Signaling	1.50	3.16E-02	1.19E-01
Atherosclerosis Signaling	1.48	3.31E-02	8.26E-02
3-phosphoinositide Degradation	1.47	3.39E-02	7.91E-02
D-myo-inositol (1,4,5,6)-Tetrakisphosphate Biosynthesis	1.46	3.47E-02	8.20E-02
D-myo-inositol (3,4,5,6)-tetrakisphosphate Biosynthesis	1.46	3.47E-02	8.20E-02
Role of NFAT in Cardiac Hypertrophy	1.43	3.72E-02	7.39E-02
Role of Tissue Factor in Cancer	1.42	3.80E-02	8.41E-02
Induction of Apoptosis by HIV1	1.42	3.80E-02	1.02E-01
Glutathione Redox Reactions I	1.42	3.80E-02	1.67E-01
Glucocorticoid Receptor Signaling	1.38	4.17E-02	6.62E-02
Thiamin Salvage III	1.37	4.27E-02	1.00E+00
Xanthine and Xanthosine Salvage	1.37	4.27E-02	1.00E+00
Sulfite Oxidation IV	1.37	4.27E-02	1.00E+00
Purine Nucleotides Degradation II (Aerobic)	1.35	4.47E-02	1.58E-01
DNA damage-induced 14-3-3σ Signaling	1.35	4.47E-02	1.58E-01

© 2000-2016 QIAGEN. All rights reserved.
