# Supplementary material for: CD161+ Tconv and CD161+ Treg Share a Transcriptional and Functional Phenotype despite Limited Overlap in TCRβ Repertoire
Source: Front Immunol. 2017 Mar 6;8:103. doi: 10.3389/fimmu.2017.00103 (PMC5337494; doi:10.3389/fimmu.2017.00103)
Supplement: Supplementary file 2 [file Image_1.PDF]

Supplementary Figures 1-7

CD161^{+} Tconv and CD161^{+} Treg a Share Transcriptional and Functional Phenotype despite Limited Overlap in TCRβ Repertoire

Chantal L Duurland*, Chrysothemis C Brown, Ryan FL O'Shaughnessy, Lucy R Wedderburn

***Correspondence:** Chantal L Duurland: c.duurland@ucl.ac.uk

1. Supplementary Figures

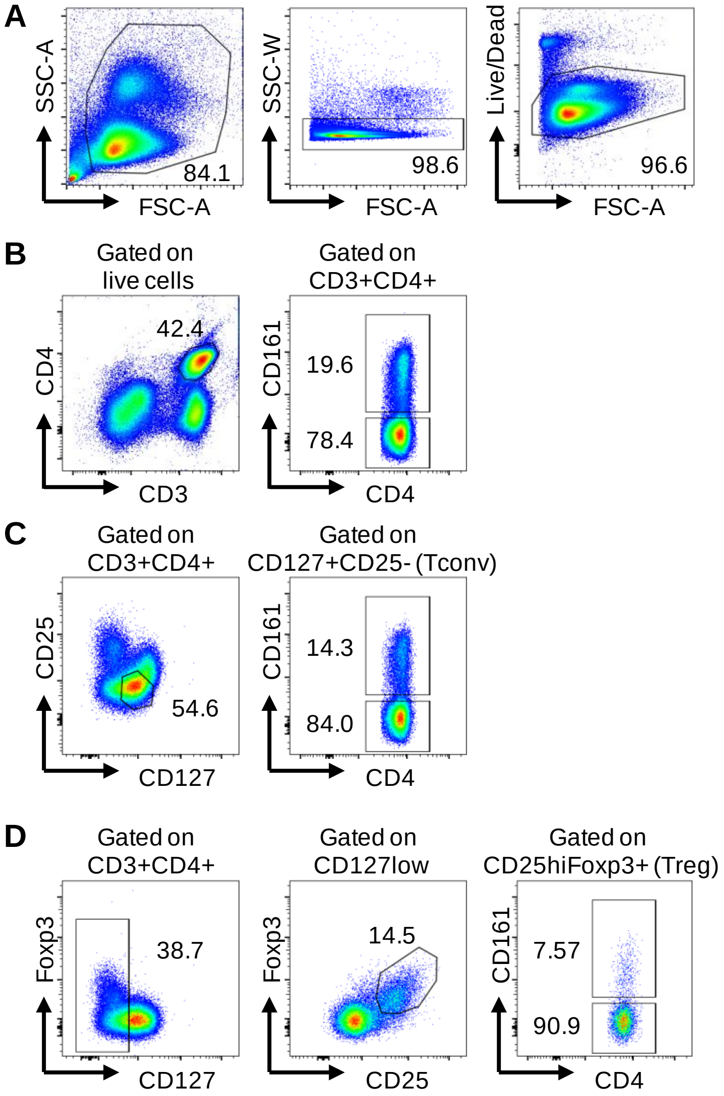

Figure S1: Gating strategy for CD161^{+} and CD161^{-} T cell populations. Representative flow cytometry plots from a healthy adult control showing the gating strategy used to identify live cells (**A**), CD3^{+}CD4^{+} T cells (**B**), Tconv (**C**) and Treg (**D**), and gating of CD161^{+} and CD161^{-} cells within CD3^{+}CD4^{+} T cells (**B**), Tconv (**C**) and Treg (**D**).

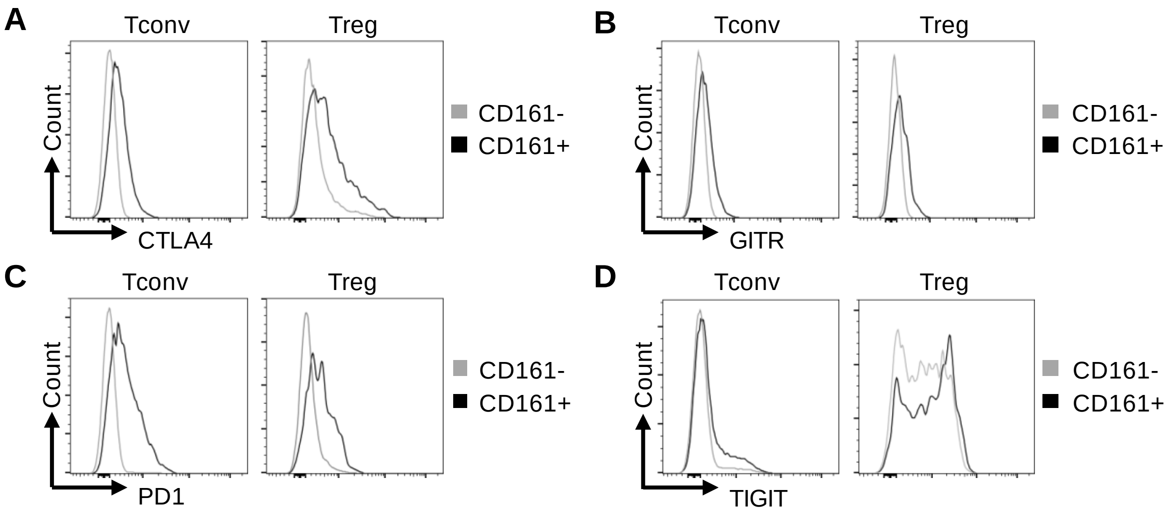

Figure S2: Expression of CTLA4, GITR, PD1 and TIGIT within CD161^{+} and CD161^{-} Tconv and Treg. (**A-D**) Representative histograms showing protein expression of CTLA4 (**A**), GITR (**B**), PD1 (**C**) and TIGIT (**D**) within CD161^{-} (gray line) and CD161^{+} (black line) Tconv and Treg from a healthy adult by flow cytometry.

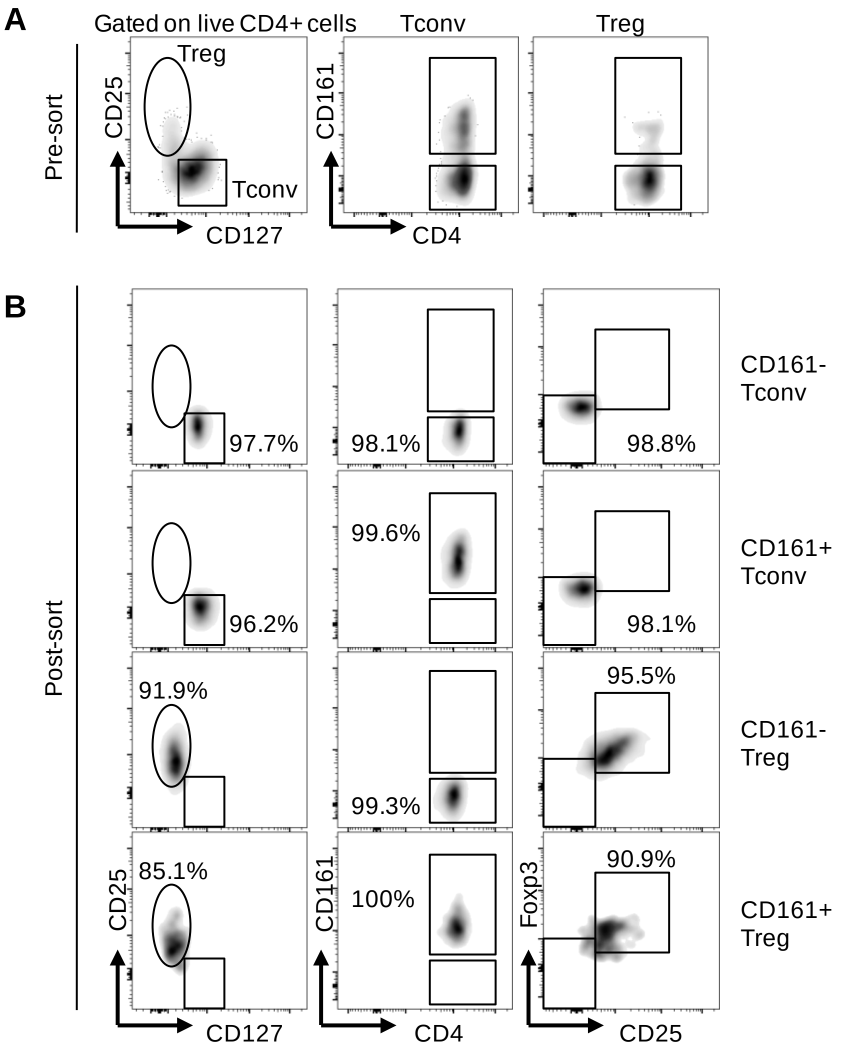

Figure S3: Sort purity of sorted CD161^{+} and CD161^{-} Tconv, and CD161^{+} and CD161^{-} Treg from a healthy adult control. Healthy adult PBMC were enriched for CD4^{+} cells and stained for CD4, CD127, CD25 and CD161 before sorting of live cells. Cells were sorted into 4 live cell populations: CD4^{+}CD127^{+}CD25^{-}CD161^{+} (CD161^{+} Tconv) or CD161^{-} (CD161^{-} Tconv) and CD4^{+}CD127^{low}CD25^{hi}CD161^{+} (CD161^{+} Treg) or CD161^{-} (CD161^{-} Treg). (**A**) Representative plots showing sorting strategy of CD161^{+} and CD161^{-} Tconv, and CD161^{+} and CD161^{-} Treg. (**B**) Representative plots showing post-sort purity for CD127 and CD25 (left), CD161 (middle) and CD25 and Foxp3 (right) expression.

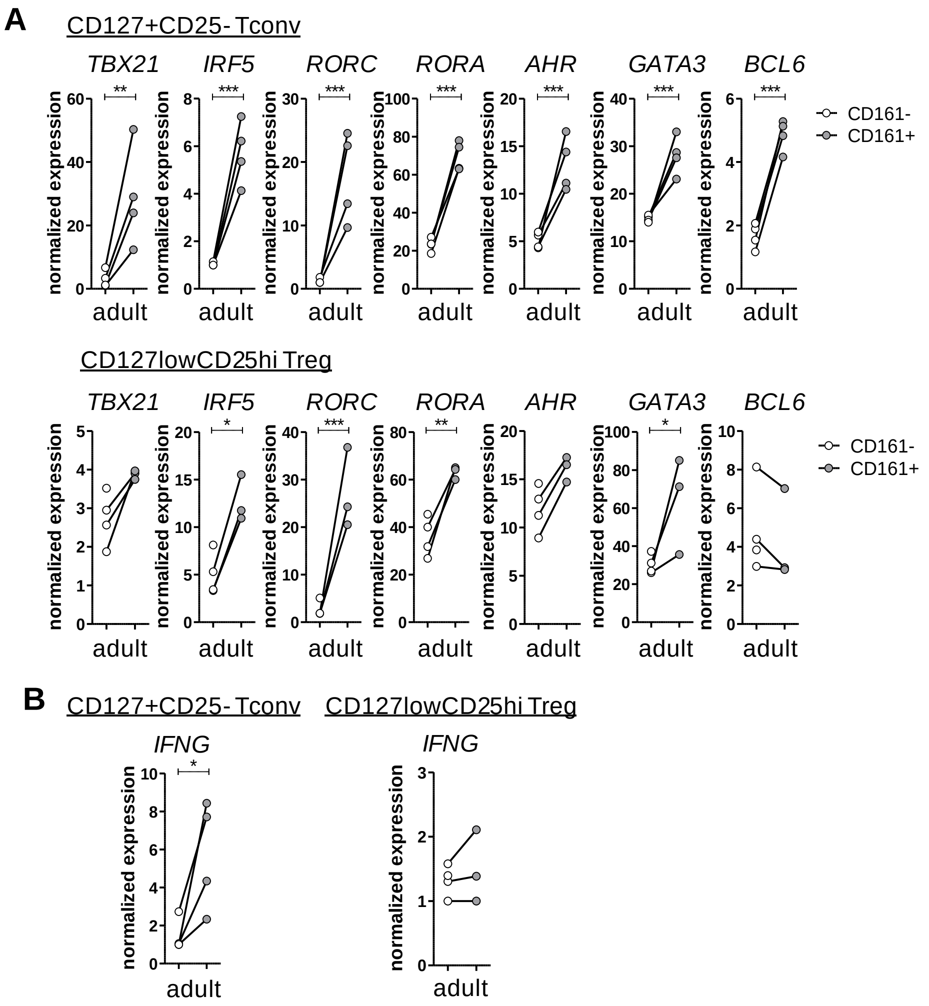

Figure S4: Expression of lineage-specific transcription factors and cytokines within $CD161^+$ and $CD161^-$ Tconv, and $CD161^+$ and $CD161^-$ Treg from RNAseq. (**A**) Normalized expression values from RNAseq for expression of *TBX21, IRF5, RORC, RORA, AHR, GATA3* and *BCL6* within $CD161^-$ (○) and $CD161^+$ (●) $CD127^+CD25^-$ Tconv and $CD127^{low}CD25^{hi}$ Treg from healthy adult controls (n=3-4). (**B**) Normalized expression values from RNAseq for expression of *IFNG* within $CD161^-$ (○) and $CD161^+$ (●) $CD127^+CD25^-$ Tconv and $CD127^{low}CD25^{hi}$ Treg from healthy adult controls (n=3-4). Statistical significance: * $P<0.05$, ** $P<0.01$, *** $P<0.001$

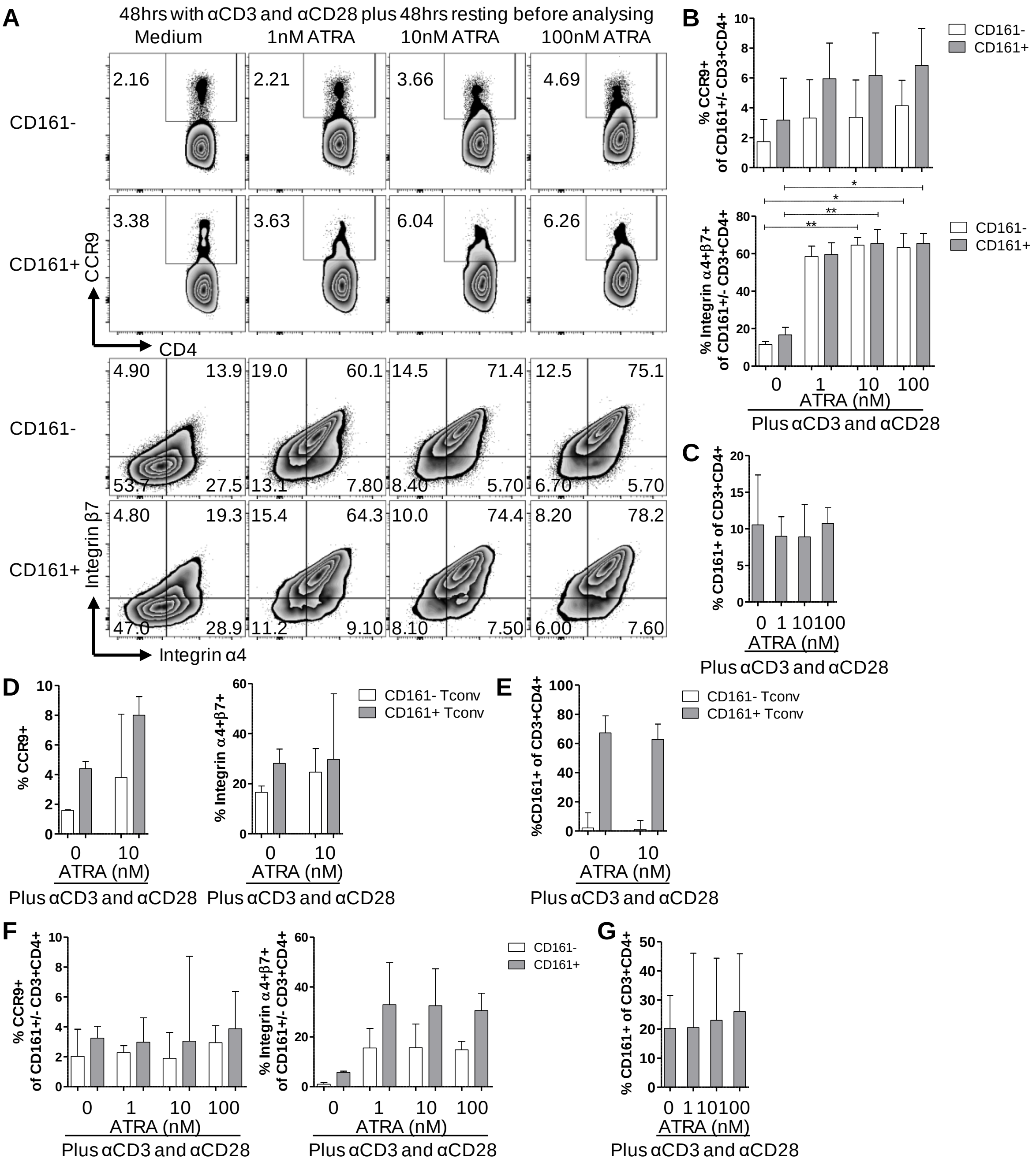

Figure S5: Response of CD161$^+$ and CD161$^-$ T cells to ATRA in presence of TCR stimulation. PBMC from healthy adults (n=6) (**A-C**), sorted CD161$^+$ and CD161$^-$ Tconv from healthy adults (**D** and **E**) (n=3), or SFMC from JIA patients (**F** and **G**) (n=3) were cultured in presence of 1µg/ml αCD3 and 5µg/ml αCD28, and ATRA for 48 hours and then rested for 48 hours. Cells were analysed for expression of CCR9, integrin α4β7 and CD161 by flow cytometry at culture conditions indicated. (**A-B**) Representative plots (**A**) and summary graphs (**B**) showing CCR9$^+$ and integrin α4$^+$β7$^+$ cells within CD161$^-$ (○) and CD161$^+$ (●) CD3$^+$CD4$^+$ T cells. (**C**) Percentage CD161$^+$ cells within CD3$^+$CD4$^+$ cells at end of culture. (**D**) Summary graphs depicting percentage CCR9$^+$ and integrin α4$^+$β7$^+$ within sorted CD161$^-$ (○) and CD161$^+$ (●) Tconv. (**E**) Percentage CD161$^+$ cells within CD3$^+$CD4$^+$ cells. (**F**) Summary graphs showing percentage CCR9$^+$ and integrin α4$^+$β7$^+$ cells within CD161$^-$ (○) and CD161$^+$ (●) CD3$^+$CD4$^+$ cells. (**G**) Percentage CD161$^+$ cells within CD3$^+$CD4$^+$ cells at end of culture. Statistical significance: * P<0.05, ** P<0.01

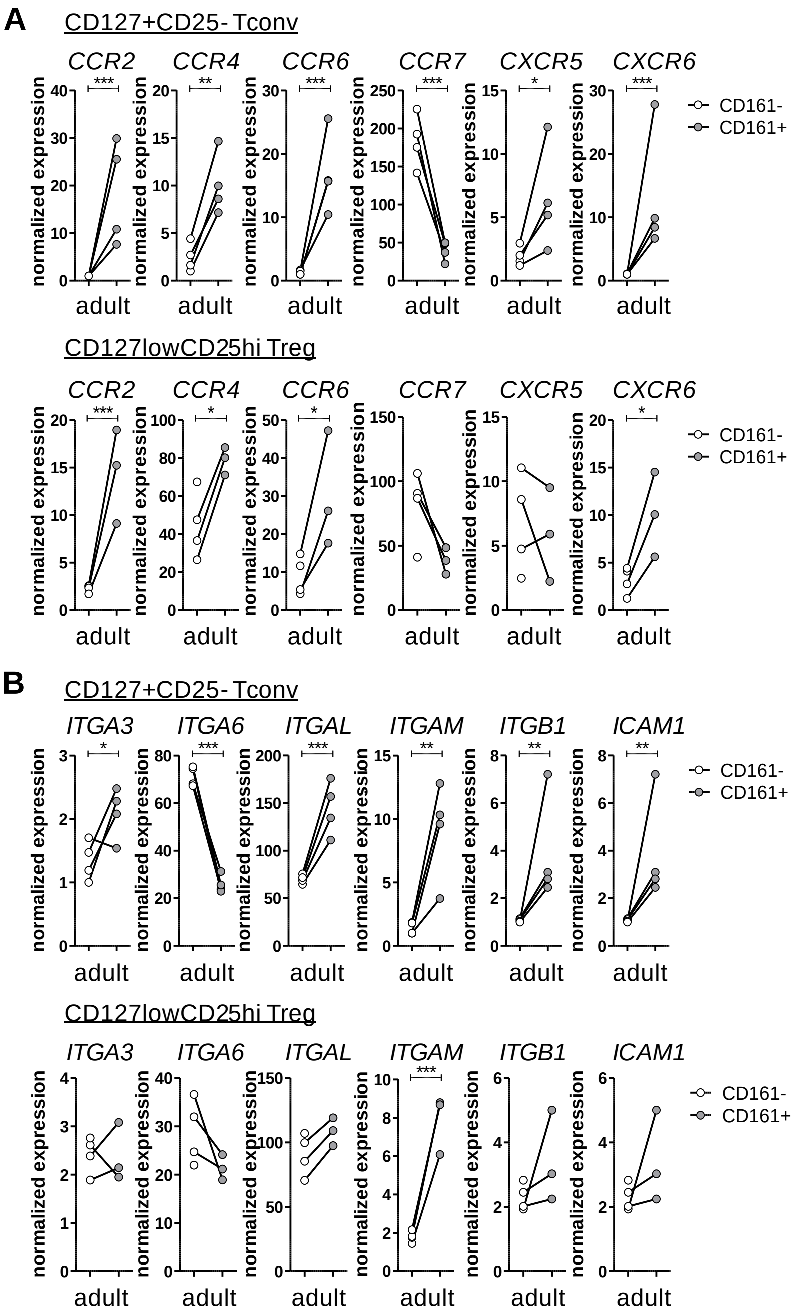

Figure S6: Expression of chemokine receptors and integrins within CD161$^+$ and CD161$^-$ Tconv, and CD161$^+$ and CD161$^-$ Treg from RNAseq. (**A**) Normalized expression values from RNAseq for expression of *CCR2, CCR4, CCR6, CCR7, CXCR5* and *CXCR6* within CD161$^-$ (○) and CD161$^+$ (●) CD127$^+$CD25$^-$ Tconv and CD127^{low}CD25^{hi} Treg from healthy adult controls (n=3-4). (**B**) Normalized expression values from RNAseq for expression of *ITGA3*, *ITGA6*, *ITGAL*, *ITGAM*, *ITGB1* and *ICAM* within CD161$^-$ (○) and CD161$^+$ (●) CD127$^+$CD25$^-$ Tconv and CD127^{low}CD25^{hi} Treg from healthy adult controls (n=3-4). Statistical significance: * P<0.05, ** P<0.01, *** P<0.001

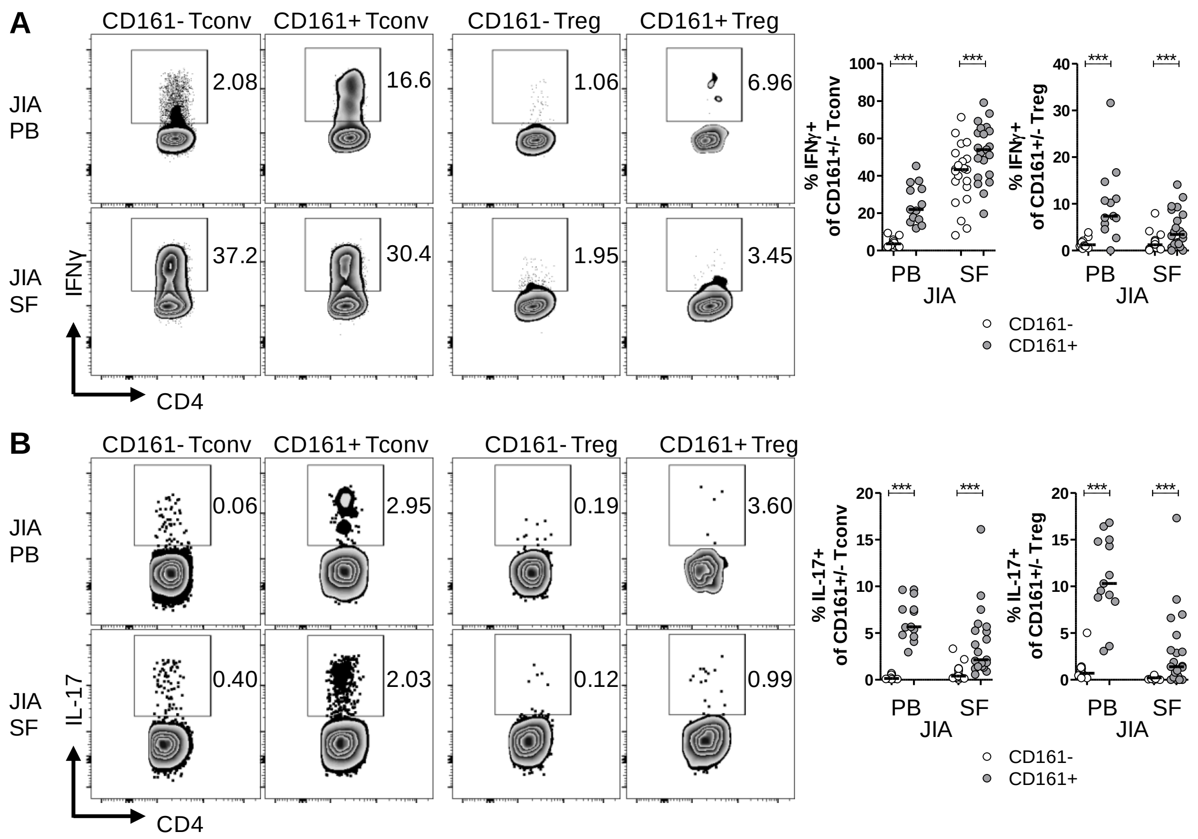

Figure S7: CD161$^+$ Tconv and CD161$^+$ Treg from JIA patients also produce pro-inflammatory cytokines. Representative plots showing IFNγ$^+$ (**A**) and IL-17$^+$ (**B**) cells within CD161$^+$ and CD161$^-$ Tconv, and CD161$^+$ and CD161$^-$ Treg cell populations in a paired JIA PB and JIA SF sample. Summary graphs showing percentage IFNγ$^+$ (**A**) and IL-17$^+$ (**B**) cells within CD161$^-$ (○) and CD161$^+$ (●) Tconv and Treg in JIA PB (n=13) and JIA SF (n=21). Statistical significance: *** *P*<0.001
